# Shotgun Kinetic Target-Guided Synthesis Approach Enables the Discovery of Small-Molecule Inhibitors against Pathogenic Free-Living Amoeba Glucokinases

**DOI:** 10.1021/acsinfecdis.3c00284

**Published:** 2023-10-11

**Authors:** Mintesinot Kassu, Prakash T. Parvatkar, Jillian Milanes, Neil P. Monaghan, Chungsik Kim, Matthew Dowgiallo, Yingzhao Zhao, Ami H. Asakawa, Lili Huang, Alicia Wagner, Brandon Miller, Karissa Carter, Kayleigh F. Barrett, Logan M. Tillery, Lynn K. Barrett, Isabelle Q. Phan, Sandhya Subramanian, Peter J. Myler, Wesley C. Van Voorhis, James W. Leahy, Christopher A. Rice, Dennis E. Kyle, James Morris, Roman Manetsch

**Affiliations:** † Department of Chemistry and Chemical Biology, Northeastern University, Boston, Massachusetts 02115, United States; ‡ Eukaryotic Pathogens Innovation Center, Department of Genetics and Biochemistry, Clemson University, Clemson, South Carolina 29634, United States; § Department of Pharmaceutical Sciences, Northeastern University, Boston, Massachusetts 02115, United States; ∥ Department of Comparative Pathobiology, College of Veterinary Medicine, Purdue University, West Lafayette, Indiana 47907, United States; ⊥ Purdue Institute for Drug Discovery (PIDD), Purdue University, West Lafayette, Indiana 47907, United States; # Purdue Institute of Inflammation, Immunology and Infectious Disease (PI4D), Purdue University, West Lafayette, Indiana 47907, United States; ¶ Department of Cellular Biology, University of Georgia, Athens, Georgia 30602, United States; ∇ Center for Drug Discovery, Northeastern University, Boston, Massachusetts 02115, United States; ○ Barnett Institute of Chemical and Biological Analysis, Northeastern University, Boston, Massachusetts 02115, United States; ⧫ Center for Emerging and Re-emerging Infectious Diseases (CERID), Division of Allergy and Infectious Diseases, Department of Medicine, University of Washington School of Medicine, Seattle, Washington 98109, United States; †† Center for Global Infectious Diseases Research, Seattle Children’s Research Center, Seattle, Washington 98109, United States; ‡‡ Department of Chemistry, University of South Florida, Tampa, Florida 33620, United States

**Keywords:** glucokinases, kinetic target-guided synthesis, multifragment screening, small molecule inhibitors, sulfo-click reaction

## Abstract

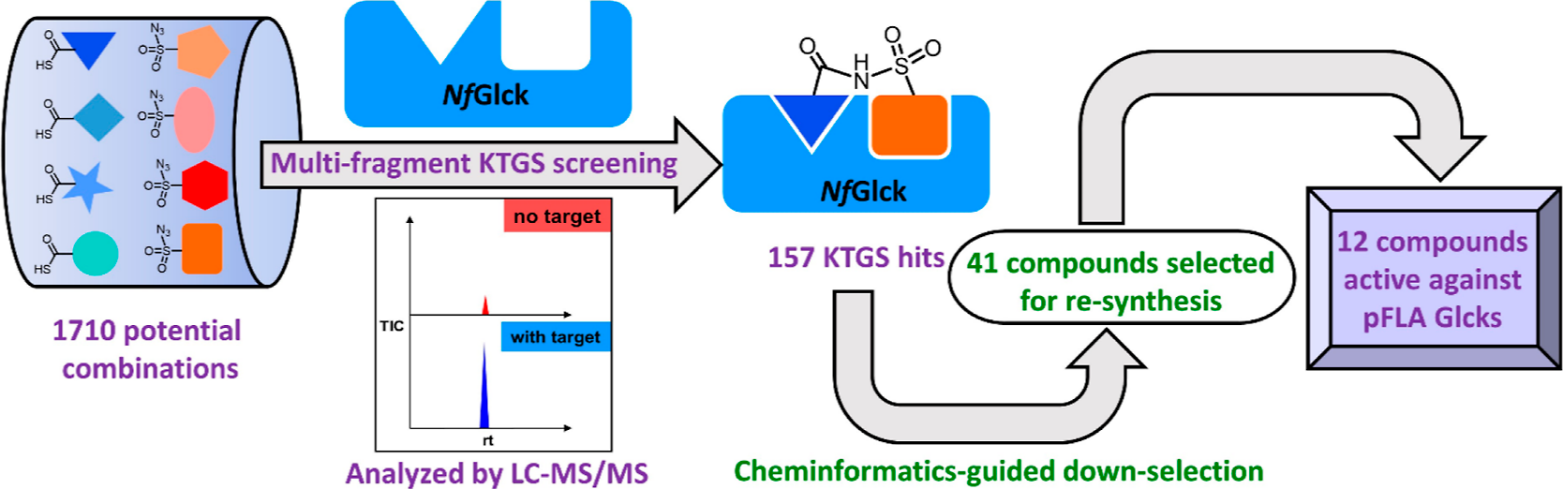

Pathogenic free-living amoebae (pFLA) can cause life-threatening central nervous system (CNS) infections and warrant the investigation of new chemical agents to combat the rise of infection from these pathogens. *Naegleria fowleri* glucokinase (*Nf*Glck), a key metabolic enzyme involved in generating glucose-6-phosphate, was previously identified as a potential target due to its limited sequence similarity with human Glck (*Hs*Glck). Herein, we used our previously demonstrated multifragment kinetic target-guided synthesis (KTGS) screening strategy to identify inhibitors against pFLA glucokinases. Unlike the majority of previous KTGS reports, our current study implements a “shotgun” approach, where fragments were not biased by predetermined binding potentials. The study resulted in the identification of 12 inhibitors against 3 pFLA glucokinase enzymes—*Nf*Glck, *Balamuthia mandrillaris* Glck (*Bm*Glck), and *Acanthamoeba castellanii* Glck (*Ac*Glck). This work demonstrates the utility of KTGS to identify small-molecule binders for biological targets where resolved X-ray crystal structures are not readily accessible.

Infections from pathogenic free-living amoebae (pFLA) are an emergent global concern for their implication in rare but life-threatening central nervous system (CNS) diseases. One pFLA, *Naegleria fowleri*, is a species of thermophilic amoebae typically found in warm freshwater and soil habitats. Commonly referred to as the “brain-eating amoeba,” *N. fowleri* causes the acute neurological disease known as primary amoebic meningoencephalitis (PAM). Infection is initiated when *N. fowleri*-contaminated water is introduced into the nasal passages, followed by penetration of the nasal epithelium and migration *via* the cribriform plate to the brain.^1,2^ Infected individuals will begin developing symptoms (headache, stiff neck, altered mental state, nausea, and vomiting) 1–9 days after exposure to *N. fowleri* and typically die within 18 days of exposure.^3^ From 1962 to 2021, only 4 of the 154 total people infected with PAM in the United States have survived the disease.^4,5^ Additionally, granulomatous amoebic encephalitis (GAE) and *Balamuthia* amoebic encephalitis (BAE) are rare CNS disorders resulting from infection by *Acanthamoeba* spp. or *Balamuthia mandrillaris*, respectively. These devastating CNS diseases disproportionately affect individuals who are immunocompromised or have an underlying health condition and they arise when amoebae are inhaled or enter the body through open wounds in the skin.^6^*B. mandrillaris*, discovered in 1986,^7^ primarily exists in soil and is the only *Balamuthia* species known to cause disease while several species of *Acanthamoeba* are known to cause disease, including *Acanthamoeba castellanii*, *Acanthamoeba polyphaga*, and *Acanthamoeba lugdunensis*.^8^ In addition to GAE, *Acanthamoeba* spp. can cause *Acanthamoeba* keratitis, a severe and extremely painful eye infection which can lead to blindness.^9^ Current treatments for these deadly CNS diseases rely on early detection and involve the administration of combination therapies, including trimethoprim, sulfadiazine, fluconazole, miltefosine, neomycin, azithromycin, and amphotericin B—typically to no avail. In spite of aggressive treatment, only a small number of patients (<2% in the United States) survive infection.^3^ Due to the striking fatality rate and limited number of treatment options available, novel therapeutics specifically designed to treat life-threatening CNS diseases caused by pFLA are urgently needed.

The discovery of potential therapeutic small molecules through fragment-based drug discovery methods has escalated in recent years. KTGS is one such method that, while the number of reported examples is still relatively low, is increasingly being applied for the discovery of novel inhibitors for a range of biological targets.^10^ Pioneered by Sharpless and co-workers,^11^ this approach begins with the incubation of a biological target with complementary reactive fragments. The fragments possessing the greatest binding potential will bind to available pockets, and those that bind with the proper proximity and geometrical configuration will react irreversibly to form a covalent bond (Figure 1). In KTGS, the biological target is directly involved in assembling its own multidentate ligand; it serves as a template that positions the molecules in an ideal spatial arrangement, thereby lowering the activation energy and accelerating the ligation reaction. Theoretically, the assembled product should have a higher affinity for the target than the individual fragments due to its capability to form an increased number of noncovalent interactions with the target. The primary appeal of KTGS is its ability to screen many candidate compounds without the laborious and time-intensive endeavor of synthesizing each individual molecule. Since its first application in 2001, KTGS has been utilized to discover small molecule binders for numerous biological targets including acetylcholine esterase,^11,12^ DNA,^13,14^ carbonic anhydrase II,^15,16^ HIV-1 protease,^17^ Bcl-X_L_,^18−20^*Serratia marcescens* chitinase,^21^ human sirtuin 1,^22^*Mycobacterium tuberculosis* EthR,^23^ acetylcholine binding protein,^24^ Bcr-Abl,^25^ NAD kinase,^26^ biotin protein ligase,^27^ 14-3-3 protein,^28,29^ insulin-degrading enzyme,^30^ bacterial ribosome,^31,32^ protein factor Xa,^33^ cyclooxygenase-2,^34^ STAT5,^35^ MDM2,^36^ endothiapepsin,^37^ and ERAP2.^38^ KTGS’s versatility is highlighted by reports describing some of the technique’s most unique applications such as the identification of protein–protein interaction inhibitors,^18,28,29,39,40^ discovery of suitable PET imaging probes for human carbonic anhydrase II,^41^ assembly of inhibitors on *c-MYC* G-quadruplex DNA embedded on the surface of gold-coated magnetic nanoparticles,^14^ implementation of KTGS in cells,^16,32^ and the development of multicomponent KTGS methods.^35,37,42^ The vast majority of published KTGS literature employs Huisgen 1,3-dipolar cycloaddition using alkynes and azides to form 1,2,3-triazoles. This, unsurprisingly, is due to the reaction’s remarkable simplicity, high efficiency, aqueous compatibility, and bio-orthogonality—features that are attractive for many organic and bio-organic pursuits, evidenced by the large body of literature that utilizes the click reaction in these fields.^43,44^ Nonetheless, other ligation reactions besides the Huisgen cycloaddition have been successfully applied in KTGS including the thiol-Michael addition,^12^ nucleophilic substitution of alkyl halides with thiols,^45^ epoxide-opening with thiols,^28^ formation of *N*-acylsulfonamides from sulfonyl azides and thio acids *via* sulfo-click,^18,19^ amidation of activated esters,^33^ Mannich ligation,^46^ and Ugi reaction.^37^

**Figure 1:**
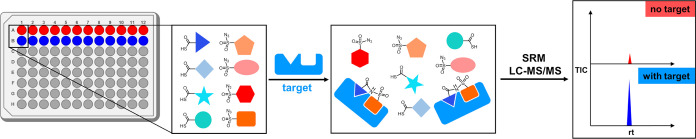
Schematic representation of the multifragment screening using KTGS. First, the complementary reactive fragments are incubated in a 96-well plate in the absence (red) and presence (blue) of the target. Fragments that bind to the target and lie in close proximity to one another irreversibly react to form a multidentate ligand. All incubations are then subjected to selected reaction monitoring (SRM) LC–MS/MS analysis, where ligated products are deemed as hits if their concentrations are amplified in the presence of the target when compared to the absence of the target.

In nearly all the reported KTGS examples, initial anchor molecule(s) are identified *a priori* through docking-based virtual screening methods, where candidate molecules are selected based on binding energies at the target’s active site, geometrical arrangement, and total occupied space. This strategy improves the likelihood of identifying hits in the subsequent screening process as it confirms the binding potential of at least one of the coupling partners and ensures the compound binds with the proper conformation and positioning. In fact, it has even been stated that coupling partners cannot be chosen at random and must be selected based on hits originating from docking studies and/or high-throughput screens.^25^ Here, we describe the first shotgun KTGS approach performed on *N. fowleri* glucokinase (*Nf*Glck) for the identification of pFLA Glck inhibitors using our previously demonstrated^47^ multifragment sulfo-click strategy with LC–MS/MS detection. We use the term “shotgun” to highlight that the selection of coupling partners was made at random and was not guided by computational methods or high-resolution X-ray crystal structures. By proceeding in this unbiased fashion, the potential of KTGS can be explored in drug discovery projects where limited structural information is available. We identified a total of 157 hits from the protein-templated experiments from which, following cheminformatics analysis, 41 were selected for resynthesis and follow-up testing. Upon inspection of initial bioactivity data, 11-cluster specific analogues were added to this set for synthesis and testing. Of the 52 total compounds synthesized, 12 were identified as bioactive, exhibiting low μM affinity against *Nf*Glck (2 compounds), *A. castellanii* glucokinase (*Ac*Glck) (9 compounds), and *B. mandrillaris* glucokinase (*Bm*Glck) (9 compounds). Several lacked notable activities against the human orthologue Glck, suggesting suitable specificity.

## Results and Discussion

Glucokinases are hexose phosphotransferases involved in carbohydrate metabolism, where they convert glucose to glucose-6-phosphate. This enzymatic transformation is the first step in glycolysis, glycogen synthesis, and the pentose phosphate pathway. The pFLA harbors a single glucokinase, with limited biochemical and sequence similarity to the human counterpart.^48^ We previously demonstrated the importance of glucose to *N. fowleri*, reporting a decrease in cellular growth and an increase in defective cyst formation in media that lacked glucose or that contained the known glycolytic inhibitor 3-bromopyruvate.^49^ Additionally, we identified several potent *Nf*Glck inhibitors, with the two most potent, **MMV688271** and **SID856002** (ebselen), exhibiting submicromolar IC_50_ values. Unfortunately, these two compounds lacked significant selectivity over the human variant (*Hs*Glck) and **MMV688271** was not active against the related Glcks from *Ac*Glck or *Bm*Glck.^48^ However, through structure–activity relationship (SAR) analysis, additional compounds displaying selective activity against the 3 pFLA Glcks (when compared to activity against *Hs*Glck) were discovered, although these did not impact amoebae viability *in vitro*. Promising results of the compounds demonstrating selectivity inspired continued investigation toward the identification and development of the next-generation inhibitors.

### Multifragment KTGS Screen against *Nf*Glck

The fragments used in the KTGS experiment were a random selection of 38 sulfonyl azides (**SZ1–SZ38**) and 45 thio acids (**TA1–TA45**) from our in-house fragment library developed from our previous work.^47^ (Figure S1) The thio acids were prepared as fluorenylmethyl thioesters from corresponding carboxylic acids, then subsequently deprotected with 5% DBU *in situ* prior to carrying out the templation experiments; as previously described.^19^ Carboxylic acids and sulfonyl azides used in this study were either commercially available or synthesized as previously reported.^47^ The investigation of *Nf*Glck-templated *N*-acylsulfonamide (**SZTA**) formation commenced with the incubation of fragments in a multicombinatorial fashion in the presence and absence of the parasitic enzyme. Sets of 5 thio acids were incubated with all 38 sulfonyl azides, giving rise to 190 possible distinct **SZTA** products per well and 1710 combinations in total. After 12 h, the template-mediated formation of corresponding **SZTA**s was assessed using selected reaction monitoring (SRM) liquid chromatography–mass spectroscopy (LC–MS/MS). The advantage of this highly sensitive analytical technique is the unambiguous determination of the assembled products as a result of multiple layers of ion filtering. Subsequent analysis of the reaction mixtures revealed 157 instances of protein-templated **SZTA** formation. This was identified by comparison of the concentrations of the 1710 possible assembled products between the control incubations that lacked the *Nf*Glck enzyme and those containing *Nf*Glck. Products that exhibited a concentration amplified by a factor of 5 or greater in the presence of protein were considered hits (Figure S2). We selected an amplification coefficient of 5 as the cutoff in order to encompass a reasonable amount of chemical space for the down-selection process (see Supporting Information—S6). Typically, the experiment is repeated with an endogenous substrate as a competitor to the fragments, adding control but reducing the hit count. Here, due to a lack of suitable *Nf*Glck inhibitors for the competition experiment, we settled on an amplification factor of 5. While this choice could lead to a higher hit rate than a more stringent cutoff (10×, *etc.*), it diminishes the risk of overlooking potent hit compounds.

### Down-selection of the Hit Compounds Using Cheminformatics

**Figure 2:**
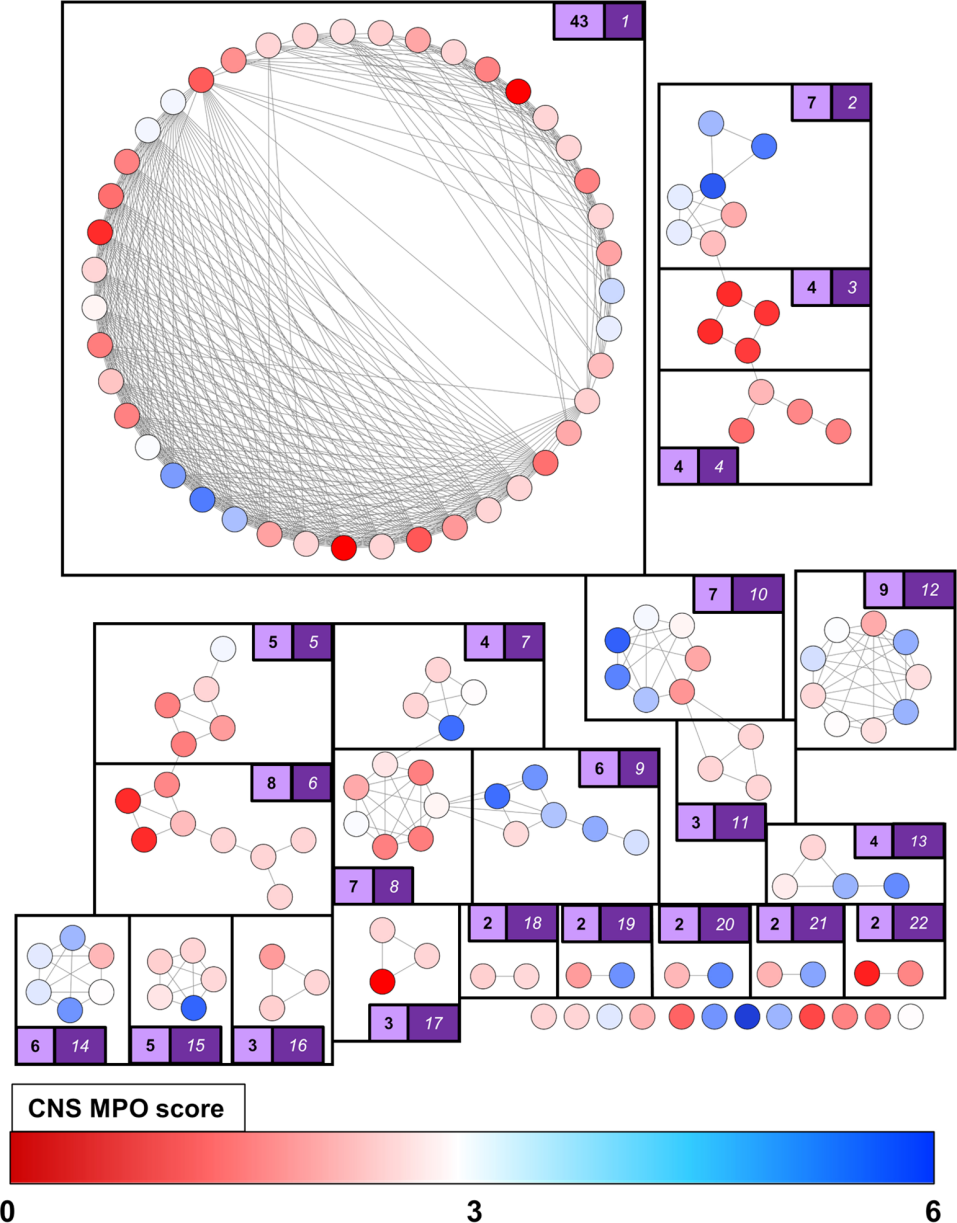
Cluster map of *N*-acylsulfonamide (**SZTA**) hits from the KTGS screen against *Nf*Glck. Compounds were clustered using Murcko frameworks and ECFP4 fingerprints with a Tanimoto similarity cutoff of 0.9. The white numbers in dark purple boxes correspond to the cluster number, while black numbers in light-purple boxes correspond to the number of cluster members. Compounds are colored according to CNS MPO scores, which range from 0 to 6, with red compounds indicating a lower CNS MPO score and blue compounds possessing a higher score.

Our next task was to explore the activity of hit compounds in enzyme and cell-based assays. In order to narrow the list of compounds for in-house synthesis, we carried out extended-connectivity fingerprint (4 bonds, ECFP4) clustering using Murcko frameworks generated from each compound with a Tanimoto similarity cutoff of 0.9.^50,51^ From this process, 22 clusters and 12 singletons were identified. To further guide our selection process, we calculated the CNS multiparameter optimization (MPO) scores for the hit compounds. The CNS MPO scores were developed by Pfizer as a metric to predict the propensity of compounds to penetrate the blood–brain barrier (BBB), which is crucial for CNS drugs in order to access their biological targets.^52^ This score considers six physicochemical properties and ranges from 0 to 6, with 6 indicating the optimal BBB-penetration potential and 0 indicating a lack thereof. The cluster map was then updated to reflect the CNS MPO scores of each compound, and a color gradient was applied accordingly (Figure 2).

First, since diversity was our primary criterion for compound selection, we chose compounds from as many clusters as possible. Second, we prioritized compounds within each cluster by the MPO score. Lastly, we selected compounds that were identified as hits for separate KTGS targets, which we screened in parallel with *Nf*Glck (data unpublished), in order to hasten synthetic efforts. Based on the aforementioned criteria, we selected 33 hit compounds for synthesis.

### Synthesis of *N*-Acylsulfonamides

**Scheme 1:**
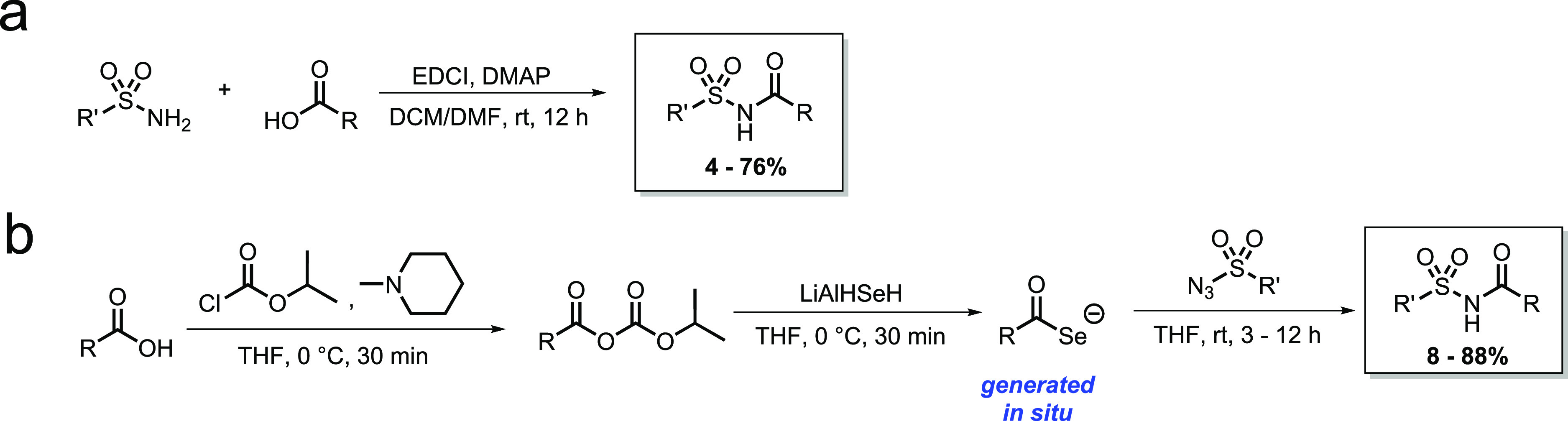
Synthesis of the *N*-Acylsulfonamides by (a) EDCI-Promoted Coupling; (b) Reaction of Selenocarboxylates, Generated from Carboxylic Acids, with Sulfonyl Azides

Construction of *N*-acylsulfonamides proceeded *via* 1-ethyl-3-(3-dimethylaminopropyl)carbodiimide (EDCI)-promoted amide coupling between corresponding sulfonamides and carboxylic acids or by the previously described method^53^ of converting carboxylic acids into selenocarboxylates *in situ* using the selenating reagent, LiAlSeH, then addition of the respective sulfonyl azide in order to yield the *N*-acylsulfonamide (Scheme 1).

### Evaluation of the Selected Hits for Activity against Amoeba Glucokinases

Synthesized compounds were tested for their activity against *Nf*Glck, *Ac*Glck, *Bm*Glck, and *Hs*Glck. Half-maximal inhibitory concentrations (IC_50_) were pursued for compounds exhibiting >40% inhibition at a concentration of 10 μM against the respective target. Our initial set of results (14 compounds evaluated) revealed only 2 active compounds belonging to cluster 6 in our similarity map. **SZ33TA45** was active against *Nf*Glck (IC_50_ = 10.9 ± 2.0 μM) and, unfortunately, exhibited a similar activity against *Hs*Glck (IC_50_ = 10.6 ± 0.45 μM). **SZ35TA30**, on the other hand, displayed potent activity against all 3 amoeba glucokinases (*Nf*Glck IC_50_ = 1.9 ± 1.1 μM, *Ac*Glck IC_50_ = 0.21 ± 0.04 μM, and *Bm*Glck IC_50_ = 0.33 ± 0.05 μM) while possessing weaker binding affinity toward the human variant (*Hs*Glck IC_50_ = 8.5 ± 0.37 μM). However, the follow-up kinetic analysis (data not shown) revealed **SZ35TA30** to be a mixed inhibitor with respect to ATP, suggesting an allosteric engagement of the compound with the target. Interestingly, the 3 pFLA Glcks share highly conserved active sites that differ structurally from *Hs*Glck, raising the possibility that **SZ35TA30** inhibits the pFLA Glcks by binding near the conserved substrate binding sites.^48^

**Figure 3:**
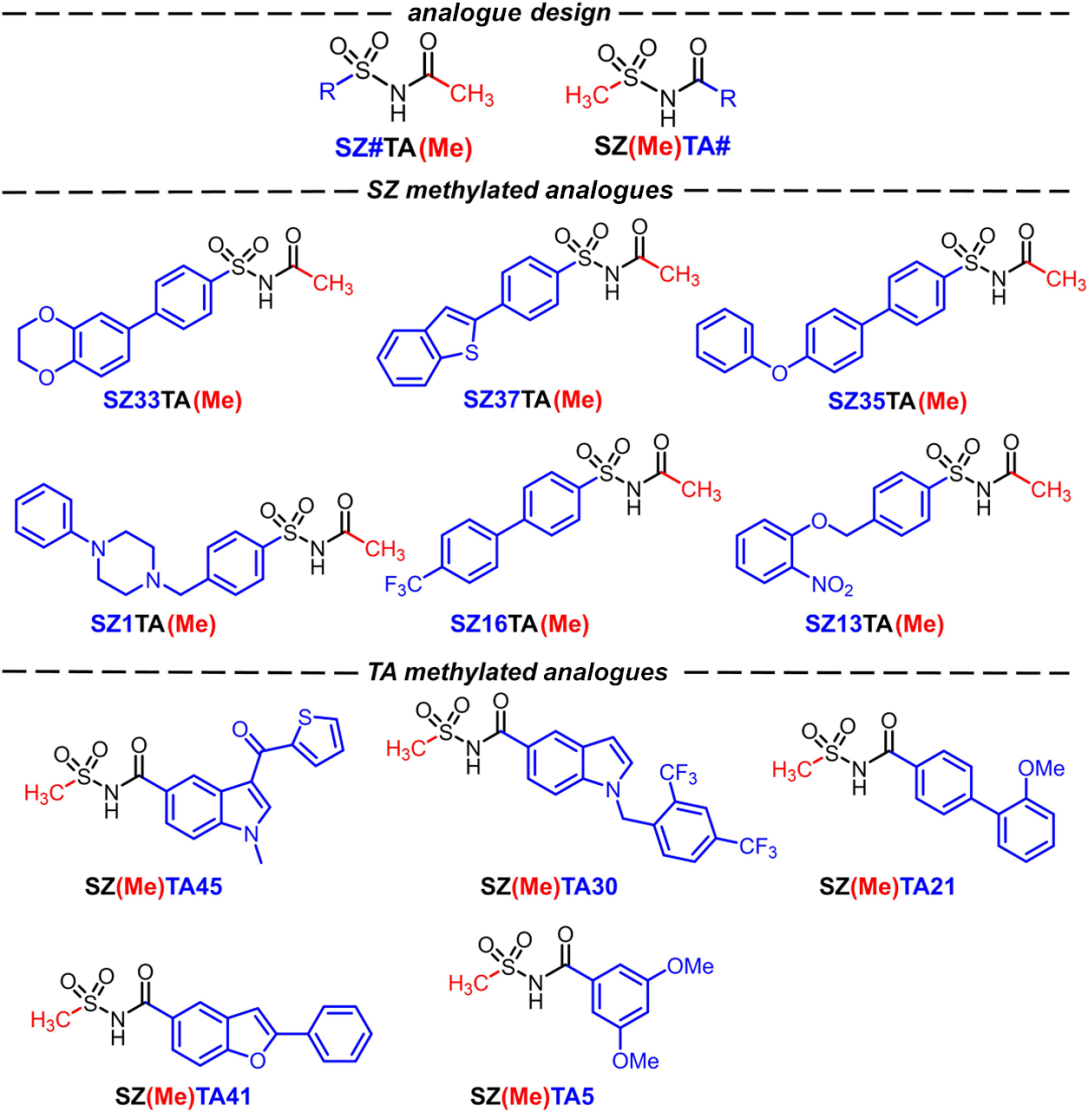
Structures of the synthesized methylated analogues. **SZ** and **TA** methylated analogues retain sulfonyl azide and thio acid functionalization, respectively, while the opposite portion of the *N*-acylsulfonamide core is substituted with a methyl group.

Inspection of **SZ33TA45** and **SZ35TA30** revealed structural similarities, particularly the presence of a biphenyl-indole *N*-acylsulfonamide core. Intrigued by these results, we decided to synthesize the remainder of the hits belonging to cluster 6 along with the members of neighboring cluster 5, adding 9 additional compounds to our initial set of 33. Of the 41 synthesized compounds tested, 12 displayed activities against the amoeba glucokinases (Table 1). To our disappointment, only **SZ33TA45** and **SZ35TA30** exhibited activity against *Nf*Glck with only **SZ35TA30** displaying slight selectivity. On the other hand, 9 compounds displayed potent activity against *Ac*Glck, 6 of which exhibited IC_50_ < 5 μM with **SZ35TA30** possessing the greatest potency (*Ac*Glck IC_50_ = 0.21 ± 0.04 μM) followed by **SZ35TA41**, (*Ac*Glck IC_50_ = 1.6 ± 0.1 μM). Nine compounds also displayed activity against *Bm*Glck, 7 of which are the same hits with activity against *Ac*Glck. **SZ35TA30** was once again the most active hit displaying submicromolar affinity toward *Bm*Glck (IC_50_ = 0.33 ± 0.05 μM), followed by **SZ35TA41** (*Bm*Glck IC_50_ = 1.19 ± 0.29 μM) and **SZ33TA30**, (*Bm*Glck IC_50_ = 1.48 ± 0.13 μM). Five of the *Bm*Glck hits exhibited IC_50_ < 2 μM and all 9 exhibited IC_50_ < 10 μM. Additionally, we were delighted to observe that all *Ac*Glck and *Bm*Glck hits, aside from **SZ35TA30**, were inactive against *Hs*Glck. These data importantly suggest that amoebae-selective Glck inhibitors can indeed be developed. Unfortunately, none of the 12 compounds displayed significant activity against amoeba cultured *in vitro*. We postulate that the lack of activity against the free-living amoeba was due to the high MW and lipophilicity of the hit series, which may preclude cell-membrane penetration, thus preventing the compounds from accessing their respective targets. The origin of the fragments used in this study may help to explain these findings. Our library was initially curated to target Bcl-X_L_ and thus was designed to bind the large, hydrophobic interfaces found in protein–protein interactions.^18,19,39^ Expansion of this library to target another PPI, Mcl-1, has unsurprisingly led to a larger, more hydrophobic fragment library than would be expected for targeting enzymes.^47^ This is exemplified by average *c* Log *P* and MW values of 6.3 and 606 Da, respectively, for the 12 compounds. Further examination of shared substructural features among the hits seems to explain this phenomenon—particularly the high density of aromatic groups with limited incorporation of heteroatoms. Eight of the 12 compounds possess a substituted biphenyl moiety, and 11 compounds contain an indole, benzofuran, or benzothiophene group. We hypothesize that reducing the size of the compounds would lower the lipophilicity and mitigate the penetration issue. This approach would also enable us to identify which structural features are important for target binding. With this in mind, we synthesized a series of methylated analogues where one end of the *N*-acylsulfonamide core was substituted with a methyl group, leaving only one fragment “fingerprint”. The **SZ** and **TA** fragments used to construct the enzymatically active *N*-acylsulfonamides were incorporated into the design of the methylated analogues, leading to the synthesis of 6 **SZ** and 5 **TA** methylated analogues (Figure 3). This new series possesses more favorable *c* Log *P* and MW values, with averages of 2.2 and 346 Da, respectively. However, none of the methylated analogues exhibited activity *in vitro* nor against the individual glucokinases. We believe that this result can be attributed to one of the two reasons: (1) the reduction in size was too severe, resulting in compounds lacking the necessary pharmacophoric features needed for establishing favorable interactions with the target, or (2) the agents may still fail to reach the target in the cell. Nonetheless, our shotgun KTGS approach enabled the discovery of 12 potent amoeba glucokinase inhibitors.

**Table 1:**
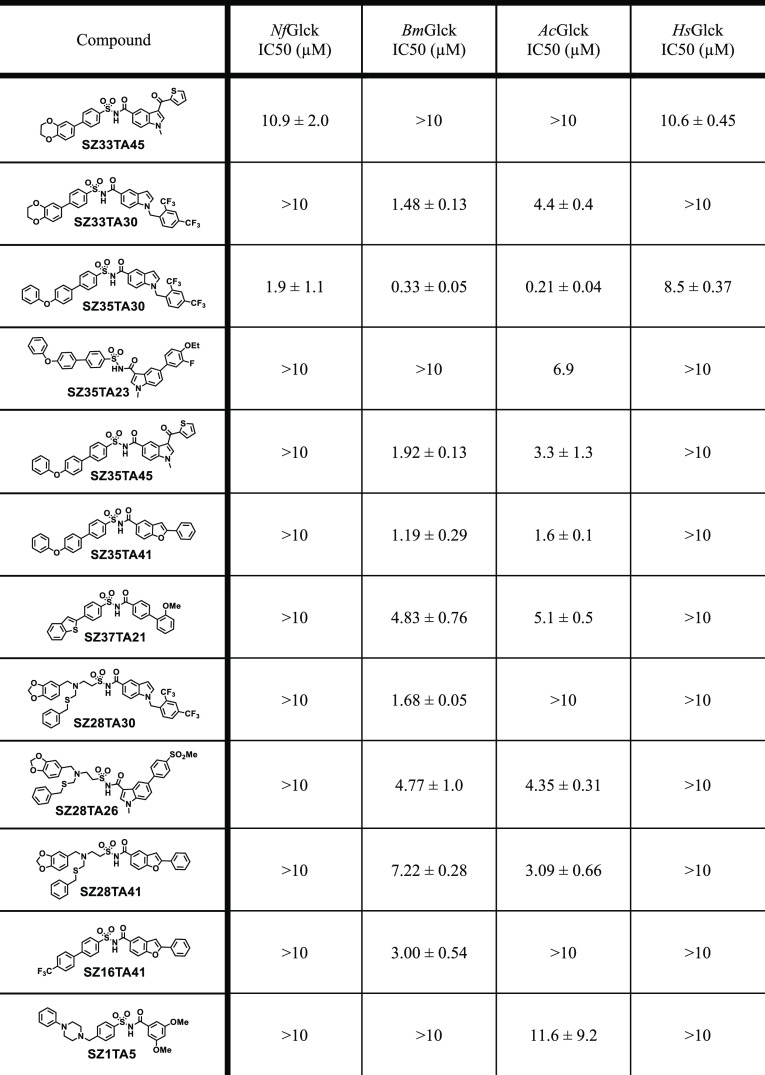
Inhibitory Activity of the Selected Hit Compounds against the pFLA Glucokinases

**Figure 4:**
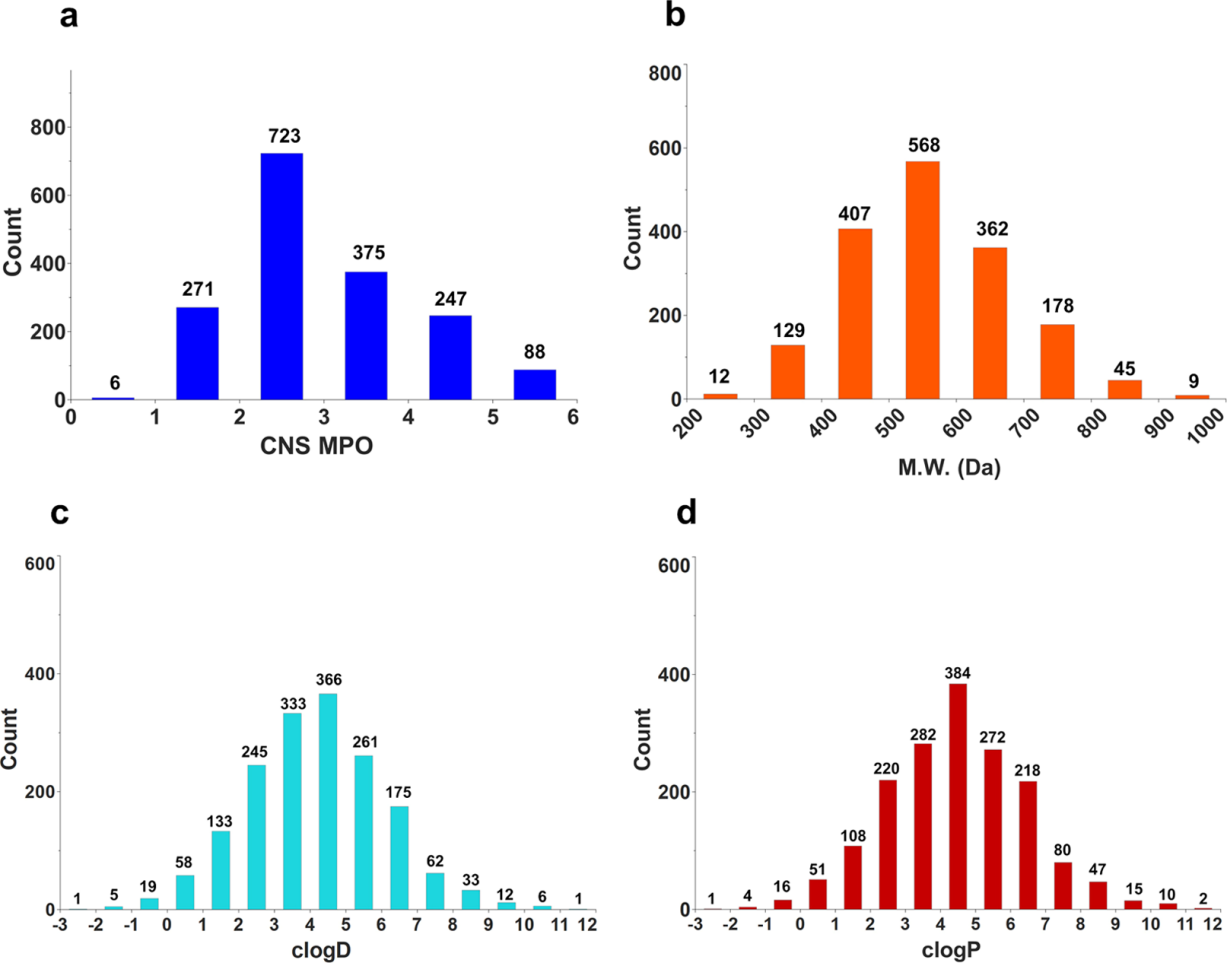
Histogram charts depicting the (a) central nervous system multiparameter optimization (CNS MPO) scores, (b) molecular weight (MW), (c) calculated octanol/water partition coefficient at pH 7 (*c* Log *D*), and (d) calculated octanol/water partition coefficient (*c* Log *P*) profile of the enumerated set of 1710 *N*-acylsulfonamides (**SZTA**s). All physicochemical values were calculated using Jchem for Excel (Chemaxon), and histograms were generated with StarDrop (Optibrium).

**Figure 5:**
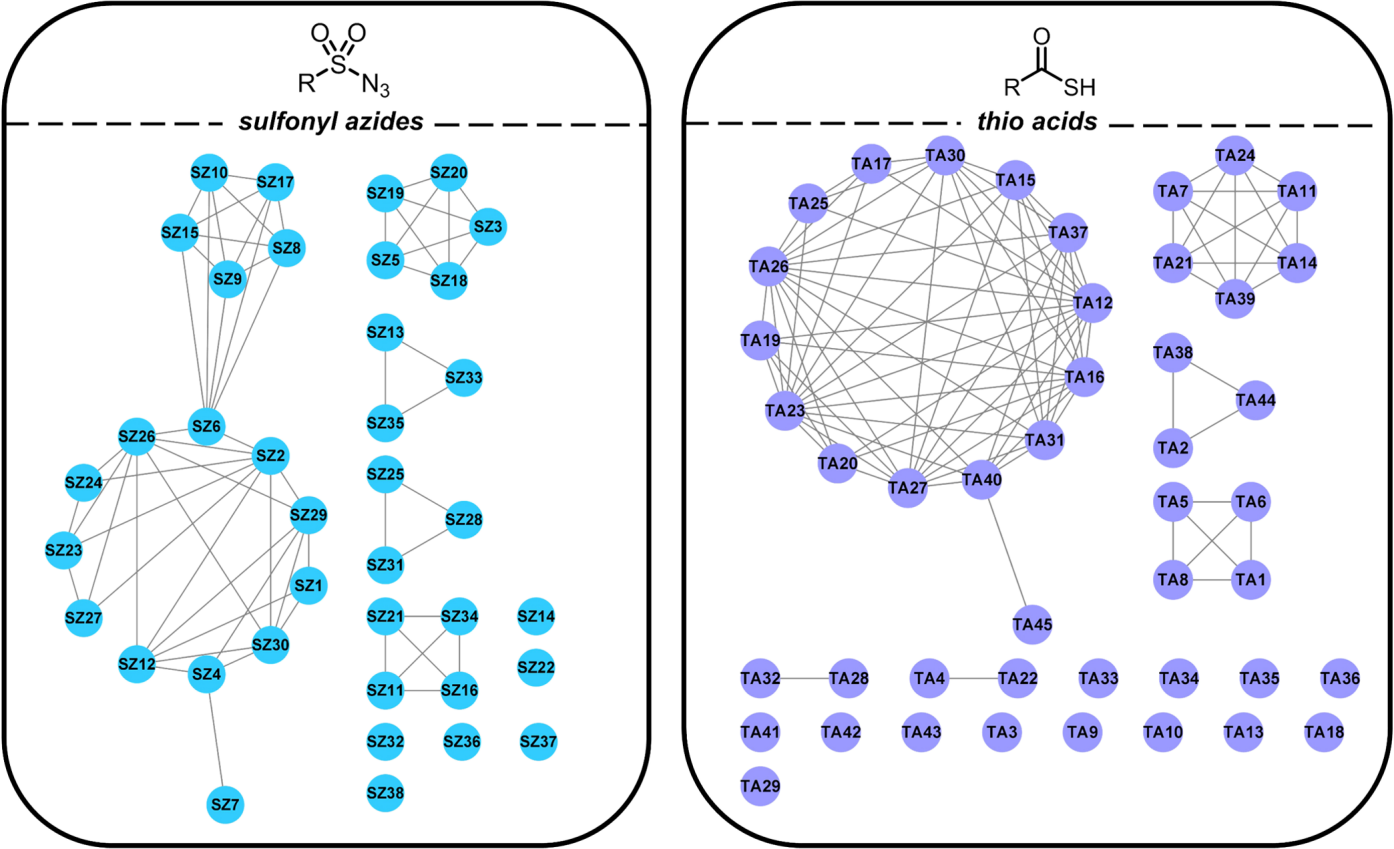
Cluster maps of sulfonyl azide (**SZ**) and thio acid (**TA**) fragment libraries. The libraries were clustered using Murcko frameworks and ECFP4 fingerprints with a Tanimoto similarity threshold of 0.8.

Our approach has demonstrated the feasibility of KTGS using a random selection of fragments with unknown binding potential toward the protein target. The consequence of this approach was the generation of fragments with less-than-ideal physicochemical traits. Future shotgun KTGS workflows would benefit from a ligand-based virtual screening step to enrich the fragment library with more “drug-like” compounds. A confirmatory step in this process could include a virtual enumeration of all possible **SZTA** combinations with the proposed fragments followed by subsequent physicochemical property analysis of the resulting data set *a priori* to fragment selection. We enumerated our current fragment library and generated 1710 **SZTA** combinations *in silico* that were subsequently assessed for BBB-penetrance potential (CNS MPO score), size (MW), and hydrophobicity (*c* Log *P*, *c* Log *D*) (Figure 4). The average CNS MPO score of the data set is 3.0 with 1000 compounds (58%) scoring below 3.0. Importantly, 10 of the 12 enzyme-active compounds are found in clusters 5 and 6 and score poorly, with an average CNS MPO score of 2.6. Furthermore, of the 1710 possible **SZTA** combinations, 68% possess a MW > 500 Da, 54% have *c* Log *P* values >4, and 60% have *c* Log *D* values >4. Given the numerous opportunities for hydrogen bonding within the Glck active site, coupled with kinetic analysis data supporting allosteric binding of our top inhibitor, one could surmise that the current fragment library is suboptimal from a medicinal chemistry perspective and confirms our initial suspicions. In addition, we suspect that higher diversity among our fragments may improve our chances of success. We clustered the **SZ** and **TA** libraries separately based on their Murcko frameworks using a Tanimoto similarity value of 0.8 and the resulting clusters are shown in Figure 5. These cluster maps confirm that our fragment libraries are limited in diversity—as evidenced by both **SZ** and **TA** libraries being organized into six dense clusters.

## Conclusions

In this study, we successfully applied the first shotgun KTGS approach for the discovery of *N*-acylsulfonamide inhibitors against *Nf*Glck, *Ac*Glck, and *Bm*Glck. The fragments used in this screen harbored no predefined binding potential to the respective targets and were selected based on commercial availability and synthetic tractability. The screen led to the identification of 157 hit compounds, 7 of which were flagged as PAINS and removed. Of the remaining 150 hit compounds, 41 were synthesized and an additional 11 methylated analogues were prepared. Twelve compounds in total displayed potent enzymatic activity against pathogenic amoebae glucokinases. Our current efforts have demonstrated that KTGS can be applied against targets with undefined structures using unoptimized fragments and still yield a bioactive hit. Furthermore, we have identified a new series of antiglucokinase compounds that may provide insights for future structure–activity relationship studies. Such studies could contribute to elucidating the role Glck plays in the survival of the pFLA. Future endeavors will focus on refining our current fragment library, aiming to enrich compounds with greater drug-like physicochemical properties, and broaden the chemical diversity of the two sets.

## Methods

### General Information

All reagents and solvents were obtained from Fisher Scientific, Sigma-Aldrich, or TCI America and used without further purification. Tetrahydrofuran (THF) was distilled from benzophenone and sodium metal under positive pressure in an argon atmosphere immediately before use. All other anhydrous solvents were purchased from VWR. Analytical thin layer chromatography (TLC) was performed on silica gel 60 F_254_ precoated plates (0.25 mm) from EMD Millipore Corp. and components were visualized by ultraviolet light (254 nm) and TLC staining solutions [phosphomolybdic acid (PMA), KMnO_4_, or a solution of Ce(SO_4_)_2_/ammonium phosphomolybdate/10% H_2_SO_4_ followed by heating]. Reported *R*_f_ values were determined for TLC. EMD silica gel 60 (particle size 40–63 μm) 230–400 mesh was used for column chromatography. ^1^H NMR spectra were recorded at ambient temperature on a 400 MHz Varian or 500 MHz Bruker NMR spectrometer in the indicated solvent. All ^1^H NMR experiments are reported in δ units, parts per million (ppm) downfield of TMS and were measured relative to the signals for chloroform (7.26 ppm), methanol (3.31 ppm), acetone (2.06 ppm), and dimethyl sulfoxide (2.50 ppm). Data for ^1^H NMR are reported as follows: chemical shift (δ ppm), multiplicity (s = singlet, d = doublet, t = triplet, q = quartet, m = multiplet), integration and coupling constant (Hz), whereas ^13^C NMR analyses are reported in terms of chemical shift. NMR data were analyzed by using MestReNova software ver. 12.0.3-21384. The purity of the final compounds was determined to be ≥95% by high-performance liquid chromatography (HPLC) using an Agilent 1260 Infinity LC instrument coupled to an Agilent 6120 single quadrupole mass spectrometer with electrospray ionization. High-resolution mass spectra were performed on an LTQ Orbitrap XL *via* loop injection with an RSLC nano pump.

### Recombinant pFLA Glck Purification

The open reading frames of *Nf*Glck, *Ac*Glck, and *Bm*Glck were cloned into pQE30, transformed into M15(pREP) *Escherichia coli* (Qiagen, Valencia, CA), and expressed as previously described.^48,54^ Briefly, *E. coli* M15(pREP4) harboring pQE30 constructs were inoculated and grown to an optical density at 600 nm (OD_600_) of ∼0.6 prior to induction with 500 μM isopropyl β-d-1-thiogalactopyranoside (IPTG). Protein from bacterial lysate was purified by nickel column chromatography (HisTrap FF; GE Healthcare Life Sciences, Pittsburgh, PA) followed by ion-exchange chromatography (HiTrap Q XL; GE Healthcare Life Sciences, Pittsburgh, PA). Protein was typically stored in 25 mM HEPES–Na (pH 7.4), 150 mM NaCl, and 50% glycerol after protein concentrations were determined by using a Bradford assay with bovine serum albumin (BSA) as a standard.

### Glucokinase Assay

Enzyme activities were measured in triplicate using a coupled reaction described previously.^54^ In a clear 96-well plate, enzyme (*Hs*Glck, *Nf*Glck, *Ac*Glck, or *Bm*Glck, all at 10 nM) was incubated with potential inhibitors (in DMSO) in their optimized assay buffer for 15 min at room temperature. For *Hs*Glck and *Nf*Glck, the assay buffer contained 50 mM TEA and 3.3 mM MgCl_2_, pH 7.4. For *Ac*Glck and *Bm*Glck, the assay buffer contained 50 mM Tris-HCl and 3.3 mM MgCl_2_, pH 8. Substrate buffer (425 μM glucose, 2.5 mM ATP, 1 U/mL G6PDH, and 0.75 mM NADP^+^) was added to each well to start the reaction for a total reaction volume of 200 μL. Immediately after addition, absorbance was measured at 340 nm at 15 s intervals for 2 min by using a BioTek Synergy H1 microplate reader (BioTek, Winooski, VT). Dose–response analysis and IC_50_ values were performed by using Prism 9.0 (GraphPad software, San Diego, CA). As a control, enzyme activity in inhibitor solvent (DMSO) was included in every assay. To solve the mode of inhibition, *Nf*Glck activity was measured with increasing substrate concentrations (0–1.25 mM) of glucose in the presence or absence of different inhibitor concentrations (0–10 μM) in technical triplicates. Data were then analyzed, and statistical analyses were performed by extra sum-of-squares *F* test to compare two models of inhibition. These results were confirmed by using the Akaike’s Information Criterion (AICc) comparison method. All statistical analyses were performed using Prism 9 software.

### pFLA Phenotypic Assays

Antiamoebic activity against *Nf*Glck, *Ac*Glck, and *Bm*Glck and cytotoxicity were determined as previously reported.^55^

### General Protocol for Multifragment KTGS with *Nf*Glck

The KTGS incubations of the entire library of 45 thio acid and 38 sulfonyl azide building blocks were accomplished by dividing the 1710 possible building block combinations into 9 individual incubation mixtures. Each of these 9 incubation mixtures was composed of 5 thio acids and 38 sulfonyl azides.

#### Preparation of the Stock Solutions Containing 38 Sulfonyl Azide Fragments

Each sulfonyl azide was first prepared as a 2 mM solution in methanol. Next, equal amounts (100 μL) of these 2 mM methanolic solutions were combined in a single vial. The solvent was completely evaporated off, and the residue was subsequently solubilized with 100 μL of fresh methanol providing a stock solution of a mixture of 38 sulfonyl azides (each sulfonyl azide at 2 mM concentration).

#### Preparation of the Stock Solutions Containing 5 Thio Acid Fragments

The stock solutions containing mixtures of 5 thio acids were prepared in a two-step process. In the first step, fluorenylmethyl thioesters were deprotected individually to yield the corresponding thio acids. Approximately 500 μg of a single thioester was weighed out in a 1.5 mL Eppendorf tube and mixed with a freshly prepared deprotection solution consisting of 5% piperidine in anhydrous DMF. The exact amount of deprotecting solution was calculated according to a previously reported protocol^56^—1.0 μL deprotecting solution (5% piperidine in anhydrous DMF) was used for 4.7 μmol of thioester. Each reaction mixture was kept at room temperature for approximately 5 min to complete the deprotection reaction and generate the corresponding thio acid. Subsequently, without further purification, each reaction mixture was diluted in the Eppendorf tube with methanol, yielding a methanolic 20 mM thio acid solution. Finally, equal amounts of methanolic 20 mM thio acid stock solutions were mixed and further diluted with methanol to obtain a stock solution of a mixture of 5 thio acids (2 mM each) which was further used for the preparation of the KTGS incubation solution.

#### Preparation of the Phosphate Buffer and Protein Stock Solutions

A phosphate buffer (pH = 7.4; 58 mM Na_2_HPO_4_, 17 mM NaH_2_PO_4_, 68 mM NaCl, and 1 mM NaN_3_), and a 10 μM Mcl-1 stock solution in phosphate buffer (pH = 7.4; 58 mM Na_2_HPO_4_, 17 mM NaH_2_PO_4_, 68 mM NaCl, and 1 mM NaN_3_) were prepared to conduct all KTGS incubation reactions.

#### KTGS Incubation Reactions

The incubation reactions were prepared in a 96-well plate by adding 1 μL of the stock solution containing 5 thio acids (2 mM of each thio acid) and 1 μL of the stock solution containing 38 sulfonyl azides (2 mM of each sulfonyl azide) to 98 μL of the target protein solution (10 μM *Nf*Glck in phosphate buffer solution). For the control incubations in the absence of the protein target, 1 μL of the stock solution containing 5 thio acids (2 mM of each thio acid) and 1 μL of the stock solution containing 38 sulfonyl azides (2 mM of each sulfonyl azide) were added to 98 μL of the phosphate buffer solution alone (protein target missing). The 96-well plate was sealed and incubated at 37 °C for 10–12 h. The incubation mixtures were then subjected to LC–MS/MS [triple quadrupole mass spectrometry detector in the multiple reaction monitoring mode (MRM following 190 parent ions in Q1 and the corresponding 5 acylium ions in Q3)], utilizing a Kinetex PFP column [2.6 μm, 100 Å (4.6 mm × 50 mm)] preceded by a Phenomenex SecurityGuard C18 cartridge. Ten μL of the incubation samples were directly injected and eluted at 37 °C using a gradient.

## Supporting Information Available

Cheminformatics, synthesis of small molecule fragments and *N*-acylsulfonamides, NMR data, and HPLC traces (PDF)
Compound clustering results (XLS)

## Abbreviations

pFLA: pathogenic free-living amoebae
CNS: central nervous system
PAM: primary amoebic meningoencephalitis
GAE: granulomatous amoebic encephalitis
KTGS: kinetic target-guided synthesis
PPP: pentose phosphate pathway
MRM: multiple reaction monitoring
LC–MS/MS: liquid chromatography–mass spectroscopy
MPO: multiparameter optimization
BBB: blood–brain barrier
*Nf*Glck: *N. fowleri* glucokinase
*Ac*Glck: *A. castellanii* glucokinase
*Bm*Glck: *B. mandrillaris* glucokinase
*Hs*Glck: *Homo sapiens* glucokinase
TA: thio acid
SZ: sulfonyl azide
SAR: structure–activity relationship
