# Supplementary material for: Shotgun Kinetic Target-Guided Synthesis Approach Enables the Discovery of Small-Molecule Inhibitors against Pathogenic Free-Living Amoeba Glucokinases
Source: ACS Infect Dis. 2023 Oct 11;9(11):2190–201. doi: 10.1021/acsinfecdis.3c00284 (PMC10644346; doi:10.1021/acsinfecdis.3c00284)
Supplement: Supplementary file 1 — id3c00284_si_001.pdf [file id3c00284_si_001.pdf]

Supplementary Information

Shotgun Kinetic Target-Guided Synthesis Approach Enables the Discovery of Small Molecule Inhibitors against Pathogenic Free-Living Amoeba Glucokinases

Mintesinot Kassu,[a] Prakash T. Parvatkar,[a] Jillian Milanes,[b] Neil P. Monaghan,[b] Chungsik Kim,[a] Matthew Dowgiallo,[a] Yingzhao Zhao,[a] Ami H. Asakawa,[c] Lili Huang,[a] Alicia Wagner,[a] Brandon Miller,[a] Karissa Carter,[a] Kayleigh F. Barrett[j], Logan M. Tillery[j], Lynn K. Barrett[j], Isabelle Q. Phan[k], Sandhya Subramanian[k], Peter J. Myler,[k] Wesley C. Van Voorhis,[j] James W. Leahy,[l] Christopher A. Rice,[d-g] Dennis E. Kyle,[g] James Morris,[b] Roman Manetsch[a,c,h,i]*

[a]Department of Chemistry and Chemical Biology, Northeastern University, Boston, MA 02115, USA.

[b]Eukaryotic Pathogens Innovation Center, Department of Genetics and Biochemistry, Clemson University, Clemson, SC 29634, USA.

[c]Department of Pharmaceutical Sciences, Northeastern University, Boston, MA 02115, USA.

[d]Department of Comparative Pathobiology, College of Veterinary Medicine, Purdue University, West Lafayette, Indiana, 47907, USA

[e]Purdue Institute for Drug Discovery (PIDD), Purdue University, West Lafayette, Indiana, 47907, USA

[f]Purdue Institute of Inflammation, Immunology and Infectious Disease (PI4D), Purdue University, West Lafayette, Indiana, 47907, USA.

[g]Department of Cellular Biology, University of Georgia, Athens, GA 30602, USA.

[h]Center for Drug Discovery, Northeastern University, Boston, MA 02115, USA.

[i]Barnett Institute of Chemical and Biological Analysis, Northeastern University, Boston, MA 02115, USA.

[j]Center for Emerging and Re-emerging Infectious Diseases (CERID), Division of Allergy and Infectious Diseases, Department of Medicine, University of Washington School of Medicine, Seattle, WA, 98109, USA

[k]Center for Global Infectious Diseases Research, Seattle Children's Research Center, Seattle, WA 98109

[l]Department of Chemistry, University of South Florida, Tampa, FL 33620

*Corresponding author

e-mail: r.manetsch@northeastern.edu

Table of Contents

Cheminformatics S3

Figure S1 – Chemical Structures of KTGS Fragments S4

Figure S2 - *NfG*lck KTGS Hit Matrix S5

ABT-737 Serum Albumin Control Experiment S6

HPLC Traces of Select Hit Compounds S7

Synthetic Procedures and Compound Characterization S12

References S94

Cheminformatics

All hits were screened for undesirable pan assay interference compounds (PAINs).[1] Compounds (7 in total) containing amino napthalenes, nitro groups, or Michael acceptors were removed from the list and were not considered for further analysis. Murcko frameworks were generated for all remaining compounds using a workflow in KNIME. Clustering was then performed in Cytoscape using a Tanimoto similarity threshold of 0.9 and 2-dimensional ECFP4 fingerprints were generated from each compound's respective Murcko framework. A total of 22 clusters containing 2 – 43 members were generated. To guide compound selection for follow-up bioactivity studies, central nervous system multiparameter optimization (CNS MPO) scores were calculated for each compound. Pioneered by Pfizer, CNS MPO scores consider six physicochemical properties (total polar surface area, pKa of most basic site, molecular weight, cLogP, cLogD$_{7.4}$, and number of hydrogen bond donors) to predict a compound's ability to penetrate the blood-brain barrier. In addition, compounds with higher CNS MPO scores possess favorable drug-like attributes (permeability, metabolic stability, etc.).[2] Physicochemical properties were calculated using JChem for Excel (Chemaxon). Guided by CNS MPO scores, one or more compounds from nearly every cluster were selected for re-synthesis.

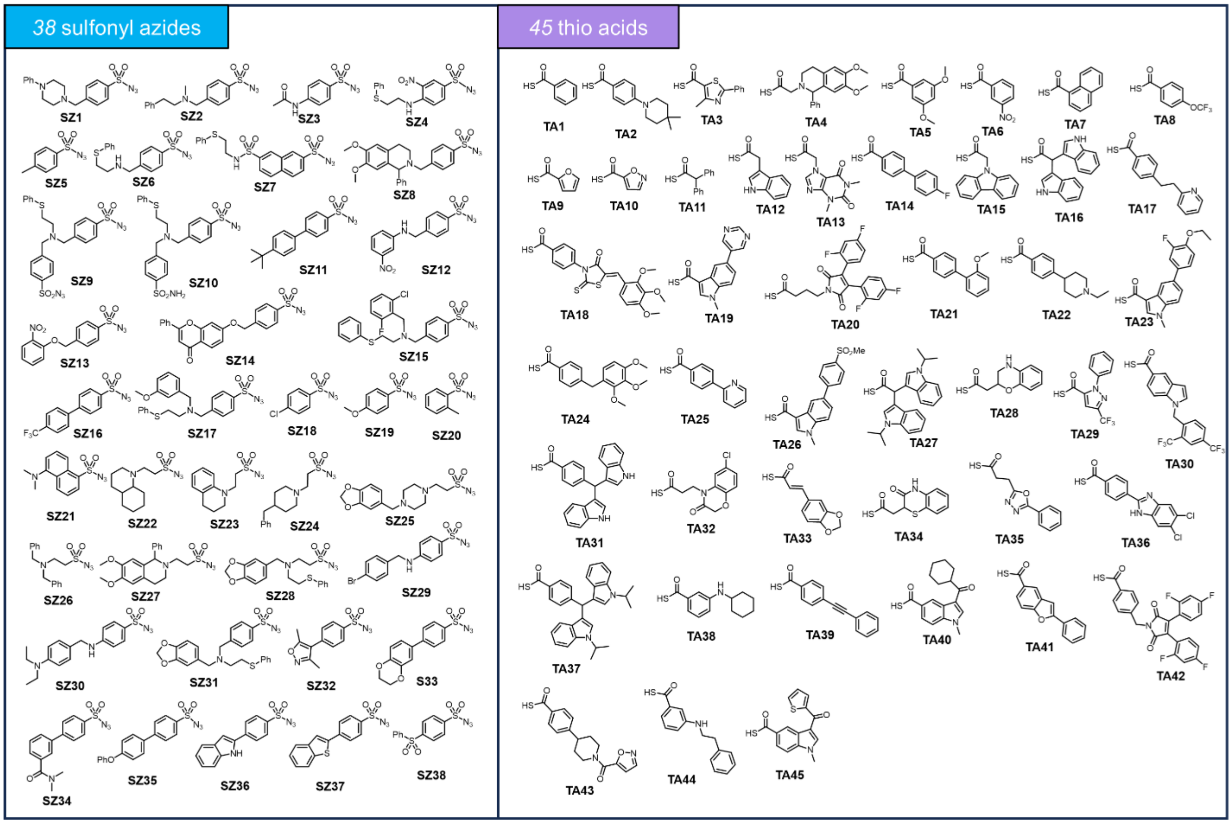

Figure S1. Chemical structures of the 38 sulfonyl azides (**SZ**s) and 45 thio acids (**TA**s) used in the kinetic target-guided synthesis (KTGS) approach aimed at targeting *N. fowleri* glucokinase (*Nf*Glck).

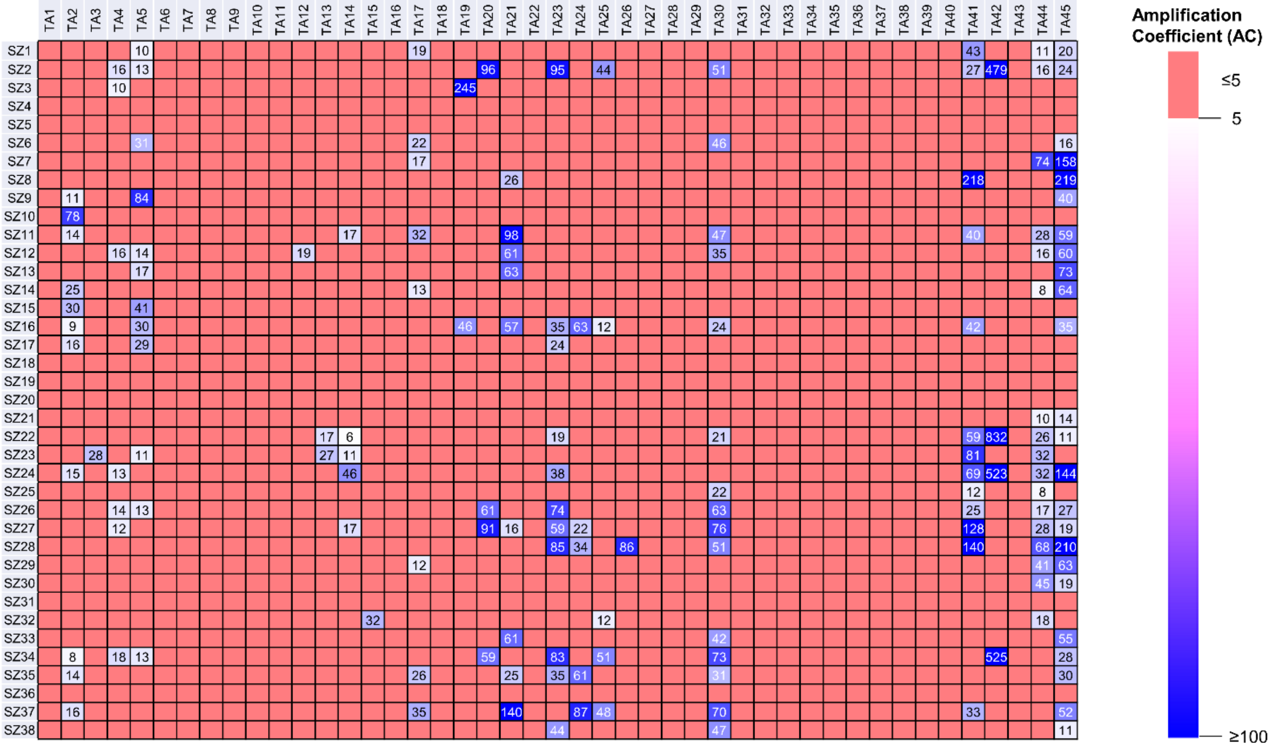

Figure S2. Hit results from *Nf*Glck template-mediated assembly of *N*-acylsulfonamides. The first column represents each of the 38 sulfonyl azides (**SZ**s) while the top row represents all 45 thioacids (**TA**s). At the intersection of any two **SZ**/**TA** combination lies the KTGS result of the corresponding *N*-acylsulfonamides (**SZTA**s). For all 1710 possible SZTA products, amplification coefficients $\left(AC = \frac{\text{peak area (protein incubation)}}{\text{peak area (non−protein incubation)}}\right)$, were measured where compounds with AC > 5 were designated as hits.

ABT-737 Serum Albumin Control Experiment

It is not uncommon for a KTGS control experiment to be performed with serum albumin in order to tease out non-specific binders. This control screen was not applied in this instance as a result of an unusually high affinity that our compounds exhibit towards serum albumin. The fragment library used in this study evolved from an initial library aimed at targeting Bcl-X_L and was derived from ABT-737 analogs, designed by Abbot. Similarly, ABT-737 analogs exhibited high affinity for serum albumin and SAR efforts were subsequently applied to reduce binding to this protein.[3]

HPLC Traces of Select Hit Compounds

HPLC of SZ33TA45 (purity = 99%)

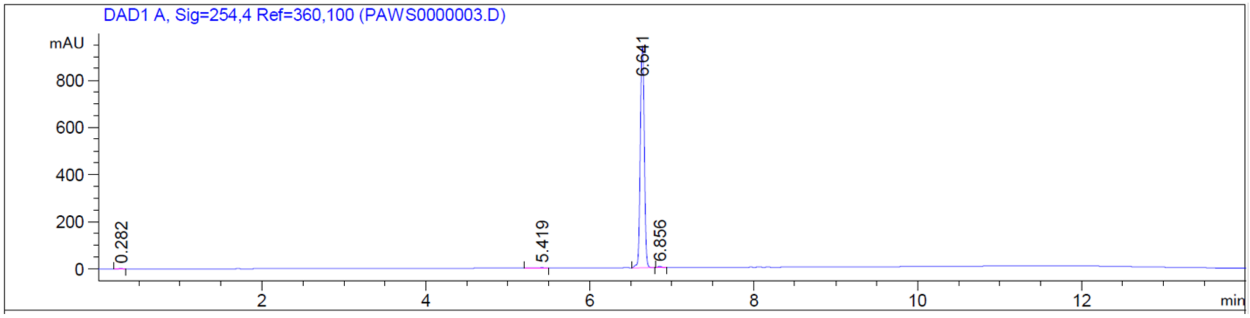

Signal 1: DAD1 A, Sig=254,4 Ref=360,100

Peak #	RetTime [min]	Type	Width [min]	Area [mAU*s]	Height [mAU]	Area %
1	0.282	BB	0.0504	5.80744	1.76562	0.1900
2	5.419	BB	0.0554	6.64300	1.87294	0.2173
3	6.641	BB	0.0513	3029.61328	946.95087	99.1152
4	6.856	BB	0.0467	14.59401	4.91331	0.4774

HPLC of SZ35TA30 (purity = 95%)

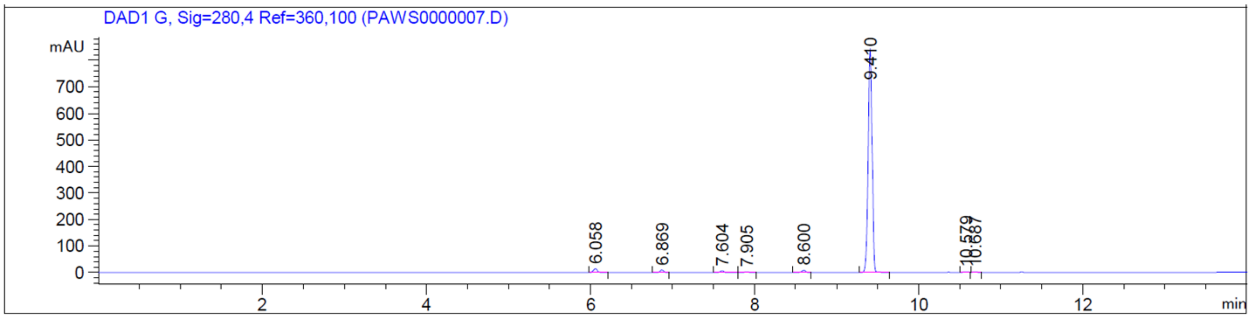

Signal 4: DAD1 G, Sig=280,4 Ref=360,100

Peak #	RetTime [min]	Type	Width [min]	Area [mAU*s]	Height [mAU]	Area %
1	6.058	BB	0.0507	43.86594	13.94948	1.5102
2	6.869	BB	0.0479	28.07447	9.12430	0.9665
3	7.604	BB	0.0503	18.77004	6.03969	0.6462
4	7.905	BB	0.0520	7.62767	2.34394	0.2626
5	8.600	BB	0.0499	27.39368	8.44220	0.9431
6	9.410	BB	0.0522	2765.34180	844.84882	95.2018
7	10.579	BB	0.0500	7.09264	2.17545	0.2442
8	10.687	BB	0.0483	6.55071	2.10774	0.2255

HPLC of SZ35TA23 (purity = 97%)

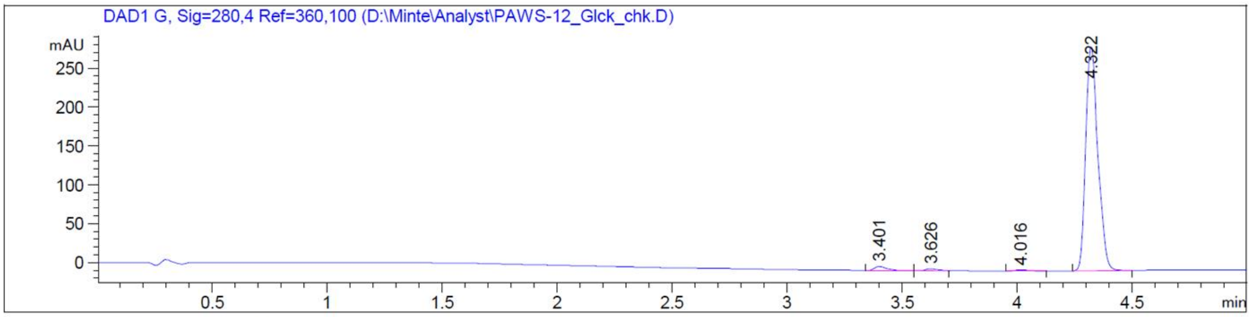

Signal 4: DAD1 G, Sig=280,4 Ref=360,100

Peak #	RetTime [min]	Type	Width [min]	Area [mAU*s]	Height [mAU]	Area %
1	3.401	BB	0.0568	20.17227	5.24916	1.8370
2	3.626	BB	0.0544	8.74925	2.40740	0.7967
3	4.016	BB	0.0593	6.50180	1.67576	0.5921
4	4.322	BB	0.0567	1062.69666	290.02609	96.7742

HPLC of SZ37TA21 (purity = 97%)

PAWS-26

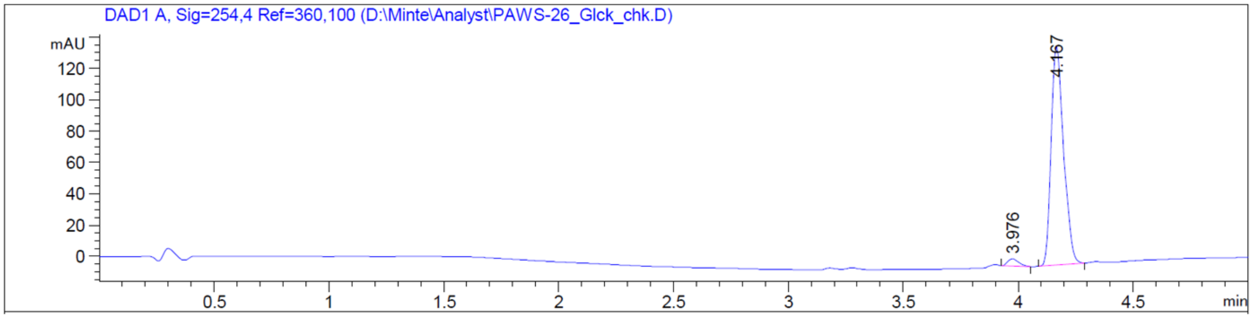

Signal 1: DAD1 A, Sig=254,4 Ref=360,100

Peak #	RetTime [min]	Type	Width [min]	Area [mAU*s]	Height [mAU]	Area %
1	3.976	BB	0.0518	14.77637	4.56456	2.7488
2	4.167	BB	0.0555	522.77197	140.09796	97.2512

HPLC of SZ35TA45 (purity = 98%)

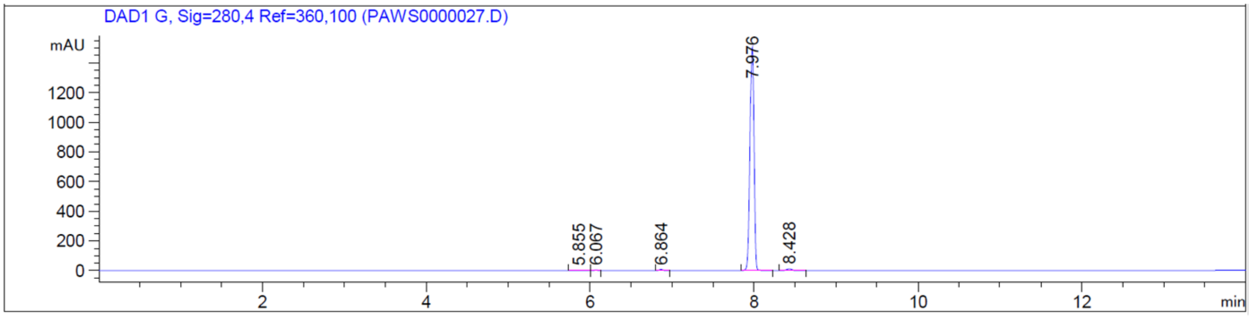

Signal 4: DAD1 G, Sig=280,4 Ref=360,100

Peak #	RetTime [min]	Type	Width [min]	Area [mAU*s]	Height [mAU]	Area %
1	5.855	BB	0.0674	5.20046	1.09503	0.1026
2	6.067	BB	0.0471	8.29198	2.75484	0.1635
3	6.864	BB	0.0481	21.63667	6.99383	0.4267
4	7.976	BB	0.0524	4990.13428	1516.70154	98.4092
5	8.428	BB	0.0713	45.53463	10.36227	0.8980

HPLC of SZ33TA30 (purity = 99%)

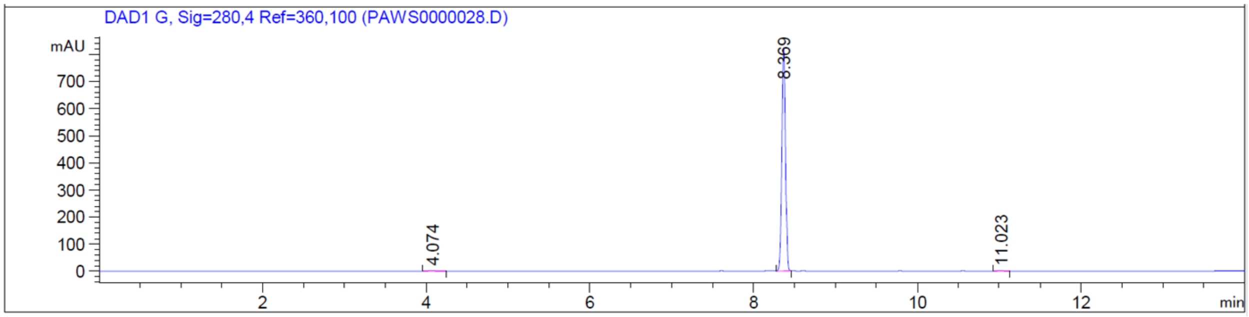

Signal 4: DAD1 G, Sig=280,4 Ref=360,100

Peak #	RetTime [min]	Type	Width [min]	Area [mAU*s]	Height [mAU]	Area %
1	4.074	BB	0.0653	9.91033	2.25182	0.3811
2	8.369	BB	0.0483	2583.38892	831.23187	99.3555
3	11.023	BB	0.0606	6.84661	1.71397	0.2633

HPLC of SZ16TA41 (purity = 97%)

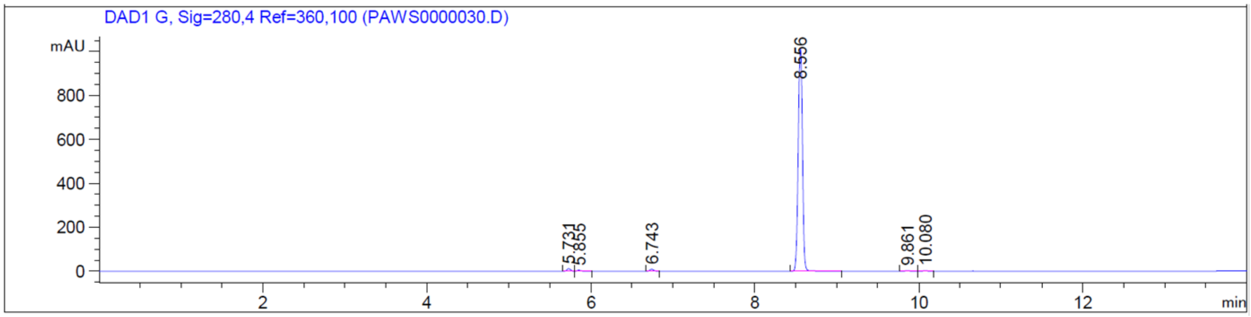

Signal 4: DAD1 G, Sig=280,4 Ref=360,100

Peak #	RetTime [min]	Type	Width [min]	Area [mAU*s]	Height [mAU]	Area %
1	5.731	BB	0.0493	36.31225	12.00513	1.0244
2	5.855	BB	0.0493	13.68375	4.27799	0.3860
3	6.743	BB	0.0483	28.73951	9.24498	0.8108
4	8.556	BB	0.0535	3451.88818	1020.26611	97.3816
5	9.861	BB	0.0524	6.71156	1.93841	0.1893
6	10.080	BB	0.0514	7.36924	2.18225	0.2079

HPLC of SZ35TA41 (purity = 97%)

PAWS-34

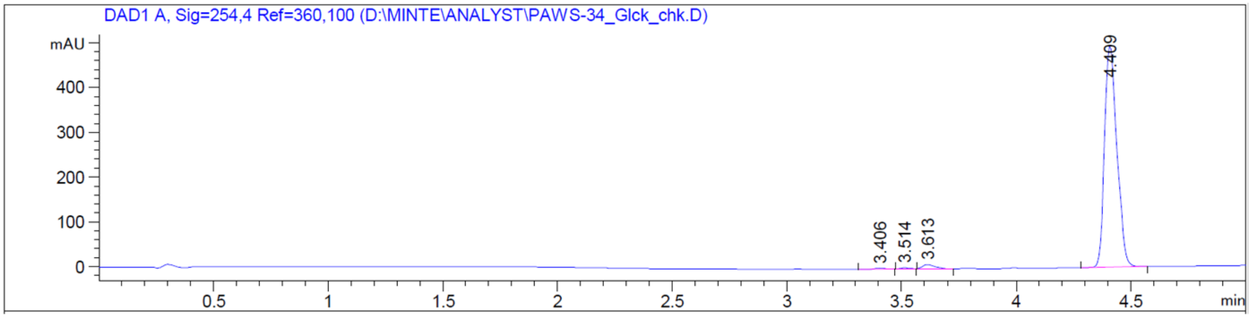

Signal 1: DAD1 A, Sig=254,4 Ref=360,100

Peak #	RetTime [min]	Type	Width [min]	Area [mAU*s]	Height [mAU]	Area %
1	3.406	BB	0.0488	7.35052	2.33222	0.3888
2	3.514	BB	0.0435	5.81752	2.15205	0.3077
3	3.613	BB	0.0550	34.24053	9.28186	1.8112
4	4.409	BB	0.0574	1843.12866	495.96814	97.4923

HPLC of SZ28TA26 (purity = 97%)

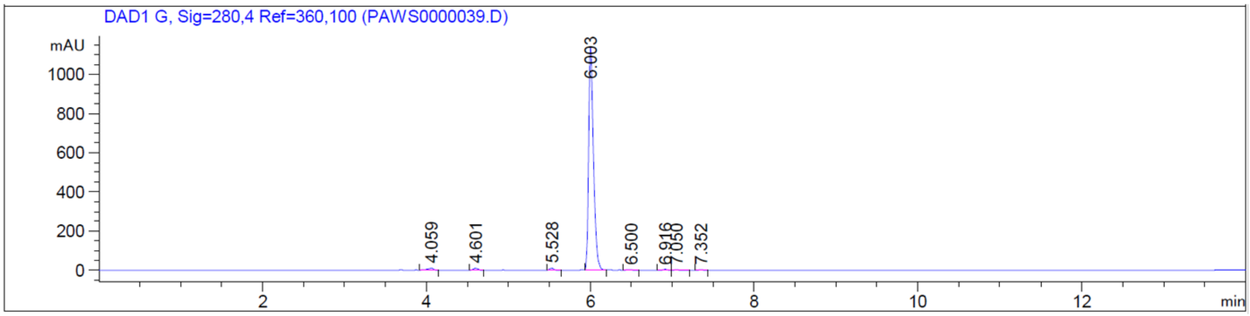

Signal 4: DAD1 G, Sig=280,4 Ref=360,100

Peak #	RetTime [min]	Type	Width [min]	Area [mAU*s]	Height [mAU]	Area %
1	4.059	BB	0.0672	48.27876	10.58660	0.9832
2	4.601	BB	0.0570	38.21587	10.36442	0.7783
3	5.528	BB	0.0495	31.47762	10.34584	0.6411
4	6.003	BB	0.0628	4759.83203	1138.10474	96.9357
5	6.500	BB	0.0674	5.09378	1.07264	0.1037
6	6.916	BB	0.0513	12.76530	3.99003	0.2600
7	7.050	BB	0.0566	7.98537	2.08875	0.1626
8	7.352	BB	0.0503	6.64890	2.02387	0.1354

HPLC of SZ28TA41 (purity = 100%)

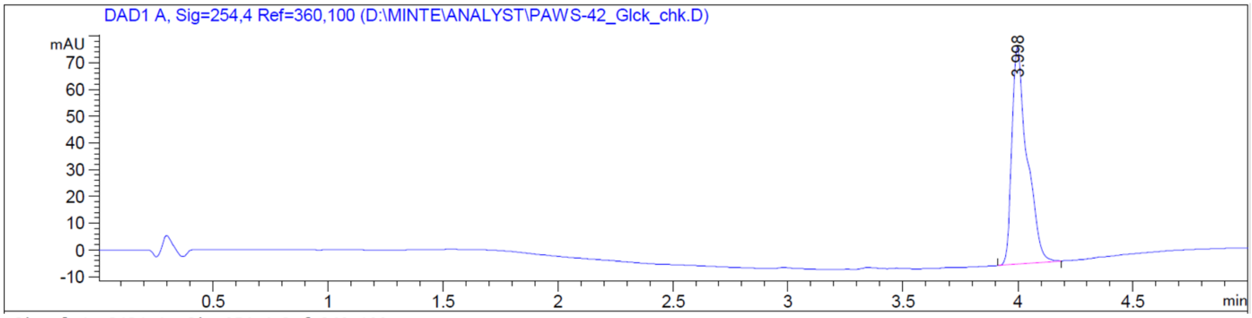

Signal 1: DAD1 A, Sig=254,4 Ref=360,100

Peak #	RetTime [min]	Type	Width [min]	Area [mAU*s]	Height [mAU]	Area %
1	3.998	BB	0.0651	371.42767	81.58107	100.0000

Synthetic Procedures and Compound Characterization

General Information. All reagents and solvents were obtained from Fisher Scientific**,** Sigma-Aldrich, or TCI America and used without further purification. Tetrahydrofuran (THF) was distilled from benzophenone and sodium metal under a positive pressure argon atmosphere immediately before use. All other anhydrous solvents were purchased from VWR Analytical thin layer chromatography (TLC) was performed on silica gel 60 F_{254} pre-coated plates (0.25 mm) from EMD Millipore Corp. and components were visualized by ultraviolet light (254 nm) and TLC staining solutions (phosphomolybdic acid (PMA), $KMnO_4$, or a solution of $Ce(SO_4)_2$/ammonium phosphomolybdate/10% H_2SO_4 followed by heating). Reported R_f values were determined for TLC. EMD silica gel 60 (particle size 40-63 μm) 230 – 400 mesh was used for column chromatography. ^{1}H-NMR spectra were recorded at ambient temperature on a 400 MHz Varian or 500 MHz Bruker NMR spectrometer in the indicated solvent. All ^{1}H NMR experiments are reported in δ units, parts per million (ppm) downfield of TMS and were measured relative to the signals for chloroform (7.26 ppm), methanol (3.31 ppm), acetone (2.06 ppm), and dimethylsulfoxide (2.50 ppm). Data for ^{1}H NMR are reported as follows: chemical shift (δ ppm), multiplicity (s = singlet, d = doublet, t = triplet, q = quartet, m = multiplet), integration and coupling constant (Hz), whereas ^{13}C NMR analyses are reported in terms of chemical shift. NMR data was analyzed by using MestReNova Software ver. 12.0.3-21384. The purity of the final compounds was determined to be ≥ 95% by high-performance liquid chromatography (HPLC) using an Agilent 1260 Infinity LC instrument coupled to an Agilent 6120 single quadrupole mass spectrometer with electrospray ionization. High-resolution mass spectra were performed on a LTQ Orbitrap XL via loop injection with an RSLC nano pump.

Synthesis of fragments

All sulfonyl azide and thioester fragments were synthesized following the same protocols found in our last report.[4]

Sulfonyl azides: **SZ1, SZ2, SZ3, SZ4, SZ5, SZ6, SZ7, SZ8, SZ9, SZ10, SZ11, SZ12, SZ13, SZ14, SZ15, SZ16, SZ17, SZ18, SZ19, SZ20, SZ21, SZ22, SZ23, SZ24, SZ25, SZ26, SZ27, SZ28, SZ29, SZ30, SZ31, SZ32, SZ33, SZ34, SZ35, SZ36, SZ37, SZ38**.

Thioesters: **TE1, TE2, TE3, TE4, TE5, TE6, TE7, TE8, TE9, TE10, TE11, TE12, TE13, TE14, TE15, TE16, TE17, TE18, TE19, TE20, TE21, TE22, TE23, TE24, TE25, TE26, TE27, TE28, TE29, TE30, TE31, TE32, TE33, TE34, TE35, TE36, TE37, TE38, TE39, TE40, TE41, TE42, TE43, TE44, TE45**.

Synthesis of *N*-acylsulfonamides: All *N*-acylsulfonamides were synthesized according to general procedure A or general procedure B. Details and modifications of reaction conditions are outlined alongside each of the corresponding compounds.

General procedure for the synthesis of *N*-acylsulfonamides (A): Carboxylic acid **CA** (1 eq) was dissolved in anhydrous DCM, DMF, or THF then EDCI (2 eq), 4-dimethylaminopyridine (1.2 eq), and sulfonamide **SA** (1 eq) were added and the reaction mixture was left to stir overnight under an argon atmosphere. The mixture was then diluted with water and extracted with ethyl acetate. The combined organic phases were dried over anhydrous Na_2SO_4 then concentrated *in vacuo*. The crude material was purified by flash chromatography to yield the corresponding *N*-acylsulfonamide.

General procedure for the synthesis of *N*-acylsulfonamides (B): The synthesis was carried out through the reaction of selenocarboxylate – generated *in situ* from carboxylic acid **CA** and LiAlHSeH – with sulfonyl azide **SZ** as previously described.[5] Briefly, to a suspension of $LiAlH_4$ (1 eq) in anhydrous THF (0.11 M) was added selenium powder (1 eq) at 0 ºC and stirred for 20 minutes under argon. Separately, the carboxylic acid was dissolved in anhydrous THF (0.11 M), cooled to 0 ºC, then 1 M isopropyl chloroformate in toluene (1 eq) and *N*-methyl piperidine (1 eq) were added and the reaction mixture was stirred for 20 minutes at 0 ºC under argon. The anhydride solution was then added dropwise to the LiAlHSeH solution and left to stir for an additional 30 min at 0 ºC under argon. A solution of sulfonyl azide (1 eq) in anhydrous THF (1 M) was added dropwise to the reaction mixture which was then stirred at room temperature or higher under an argon atmosphere. The reaction mixture was then filtered through a Celite pad which was rinsed with EtOAc. The combined organic phases were washed with 5% $NaHCO_3$, water, then brine. Subsequent drying over anhydrous Na_2SO_4 and removal of the solvent *in vacuo* afforded the crude product. The crude material was purified by flash chromatography yielding the corresponding *N*-acylsulfonamide.

Synthesis of N-((4-(1H-indol-2-yl)phenyl)sulfonyl)benzamide (SZ36TA1)

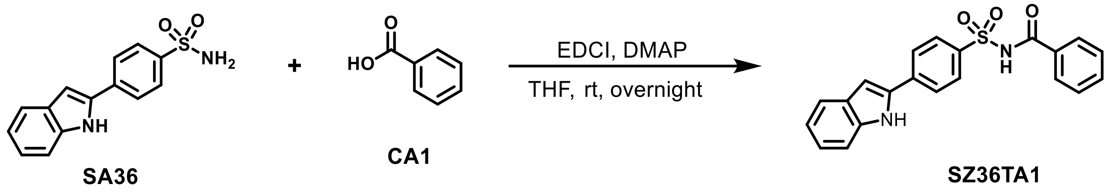

The synthesis was carried out according to general procedure **(A)** using carboxylic acid **CA1** and sulfonamide **SA36**. The reaction solvent was anhydrous THF (0.20 M), and the mixture was stirred overnight at room temperature under an argon atmosphere. The crude material was purified by column chromatography (DCM/EtOAc = 7/1 followed by DCM/MeOH = 99/1 to 95/5) to yield pure **SZ36TA1** (47%). R_f = 0.25 in 5% MeOH in DCM.

^{1}H NMR (400 MHz, CD_3OD) δ 8.12 (d, *J* = 8.4 Hz, 2H), 7.97 (d, *J* = 8.3 Hz, 2H), 7.82 (d, *J* = 7.9 Hz, 2H), 7.62 – 7.53 (m, 2H), 7.49 – 7.37 (m, 3H), 7.14 (d, *J* = 8.0 Hz, 1H), 7.05 – 6.97 (m, 2H).

HRMS-ESI (*m*/*z*): $[M+H]^+$ calculated for $C_{21}H_{17}N_2O_3S$, 377.0961 Da; found 377.0960 Da.

Synthesis of 1-methyl-N-((2-(octahydroquinolin-1(2H)-yl)ethyl)sulfonyl)-3-(thiophene-2-carbonyl)-1H-indole-5-carboxamide (SZ22TA45)

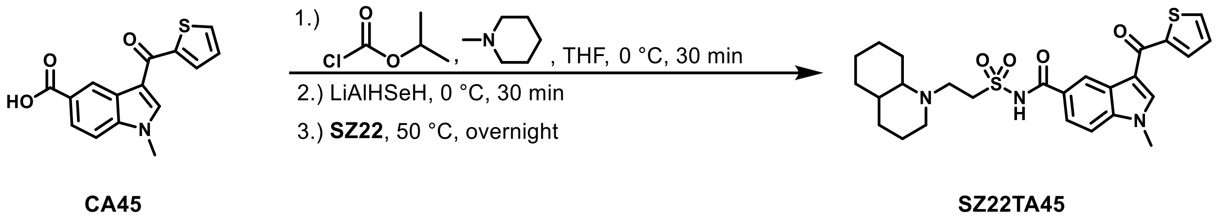

The synthesis was carried out according to general procedure **(B)** using carboxylic acid **CA45** and sulfonyl azide **SZ22**. The final reaction mixture was stirred overnight at 50 ºC. The crude material was purified by column chromatography (DCM/MeOH = 97/3 to 93/7) to yield pure **SZ22TA45** (54%). R_f = 0.14 in 5% MeOH in DCM.

^{1}H NMR (400 MHz, $CDCl_3$) δ 8.98 (s, 1H), 8.11 (d, *J* = 8.7 Hz, 1H), 7.78 (s, 1H), 7.69 (d, *J* = 3.4 Hz, 1H), 7.54 (d, *J* = 4.9 Hz, 1H), 7.21 (d, *J* = 8.7 Hz, 1H), 7.13 – 7.06 (m, 1H), 4.09 – 3.73 (m, 4H), 3.72 – 3.54 (m, 2H), 3.54 – 3.36 (m, 1H), 2.97 – 2.84 (m, 1H), 2.73 – 2.59 (m, 1H), 2.30 – 2.02 (m, 2H), 1.98 – 1.45 (m, 8H), 1.36 – 1.05 (m, 3H), 1.02 – 0.81 (m, 1H).

HRMS-ESI (*m*/*z*): $[M+H]^+$ calculated for $C_{26}H_{32}N_3O_4S_2$, 514.1835 Da; found 514.1830 Da.

Synthesis of N-((4-(2,3-dihydrobenzo[b][1,4]dioxin-6-yl)phenyl)sulfonyl)-1-methyl-3-(thiophene-2-carbonyl)-1H-indole-5-carboxamide (SZ33TA45)

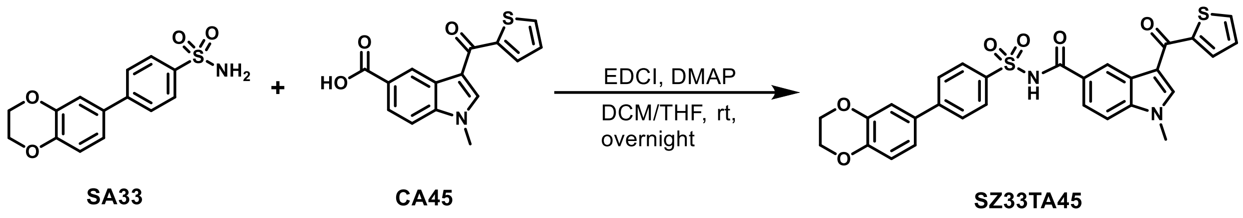

The synthesis was carried out according to general procedure **(A)** using carboxylic acid **CA45** and sulfonamide **SA33**. The reaction solvent was anhydrous THF (0.20 M) and anhydrous DCM (0.20 M), and the mixture was stirred overnight at room temperature under an argon atmosphere. The crude material was purified by column chromatography (DCM/EtOAc = 7/1 followed by DCM/MeOH = 99/1 to 97/3) to yield pure **SZ33TA45** (38%). R_f = 0.25 in 3% MeOH in DCM.

^{1}H NMR (400 MHz, $(CD_3)_2SO$) δ 12.66 (s, 1NH), 8.85 (d, *J* = 1.1, 1H), 8.56 (s, 1H), 8.08 – 7.98 (m, 4H), 7.87 (d, *J* = 8.5 Hz, 2H), 7.82 (dd, *J* = 8.7, 1.4 Hz, 1H), 7.68 (d, *J* = 8.8 Hz, 1H), 7.34 – 7.25 (m, 2H), 7.24 (dd, *J* = 8.4, 2.1 Hz, 1H), 6.98 (d, *J* = 8.3 Hz, 1H), 4.29 (s, 4H), 3.94 (s, 3H).

HRMS-ESI (*m/z*): $[M+H]^+$ calculated for $C_{29}H_{23}N_2O_6S_2$, 559.0998 Da; found 559.1000 Da.

Synthesis of 2-((2-(3,4-dihydroisoquinolin-2(1H)-yl)ethyl)sulfonyl)-1-(3,5-dimethoxyphenyl)ethan-1-one (SZ23TA5)

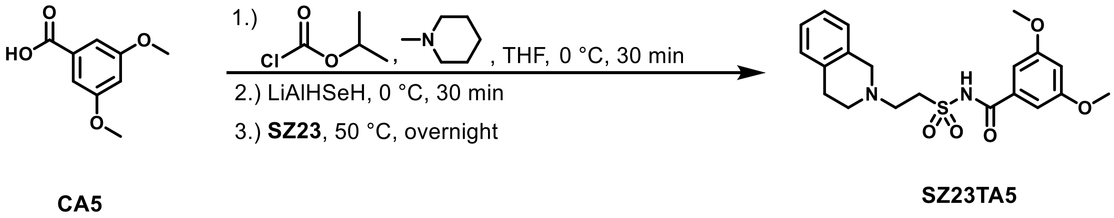

The synthesis was carried out according to general procedure **(B)** using carboxylic acid **CA5** and sulfonyl azide **SZ23**. The final reaction mixture was stirred overnight at 50 ºC. The crude material was purified by flash chromatography (DCM/MeOH) to yield pure **SZ23TA5** (37%). R_f = 0.3 in 4% MeOH/DCM.

^{1}H NMR (500 MHz, $(CD_3)_2SO$) δ 7.26 – 7.16 (m, 3H), 7.14 (d, *J* = 6.6 Hz, 1H), 7.08 (d, *J* = 2.3 Hz, 2H), 6.57 (t, *J* = 2.4 Hz, 1H), 4.26 (s, 2H), 3.74 (s, 6H), 3.68 (t, *J* = 7.4 Hz, 2H), 3.40 – 3.35 (m, 4H), 2.99 (t, *J* = 6.2 Hz, 2H).

HRMS-ESI (*m/z*): $[M+H]^+$ calculated for $C_{20}H_{25}N_2O_5S$, 405.1485 Da; found 405.1484 Da.

Synthesis of N-((2-(dibenzylamino)ethyl)sulfonyl)-1-methyl-3-(thiophene-2-carbonyl)-1H-indole-5-carboxamide (SZ26TA45)

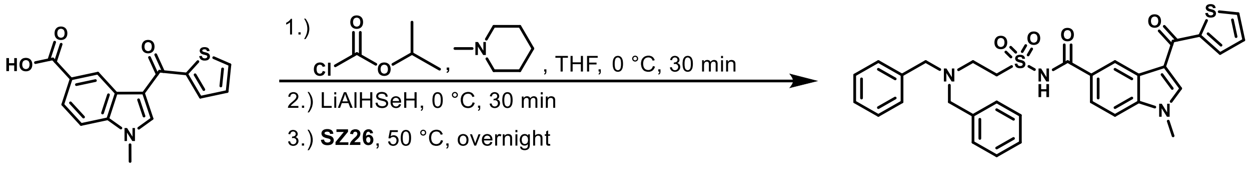

CA45 **SZ26TA45**

The synthesis was carried out according to general procedure **(B)** using carboxylic acid **CA45** and sulfonyl azide **SZ26**. The final reaction mixture was stirred overnight at 50 ºC. The crude material was purified by flash chromatography (DCM/MeOH) to yield pure **SZ26TA45** (39%). R_f = 0.3 in 2% MeOH/DCM.

^{1}H NMR (500 MHz, $CDCl_3$) δ 8.76 (d, *J* = 1.9 Hz, 1H), 7.97 – 7.86 (m, 2H), 7.77 (s, 1H), 7.64 (d, *J* = 4.8 Hz, 1H), 7.43 (d, *J* = 8.5 Hz, 1H), 7.30 (d, *J* = 7.5 Hz, 4H), 7.22 (t, *J* = 7.6 Hz, 4H), 7.19 – 7.11 (m, 3H), 3.93 (t, *J* = 2.9 Hz, 3H), 3.74 (t, *J* = 7.1 Hz, 2H), 3.65 (s, 4H), 3.08 (t, *J* = 7.1 Hz, 2H).

HRMS-ESI (*m*/*z*): $[M+H]^+$ calculated for $C_{31}H_{30}N_3O_4S_2$, 572.1678 Da; found 572.1678 Da.

Synthesis of 1-methyl-5-(pyrimidin-5-yl)-N-((4'-(trifluoromethyl)-[1,1'-biphenyl]-4-yl)sulfonyl)-1H-indole-3-carboxamide (SZ16TA19)

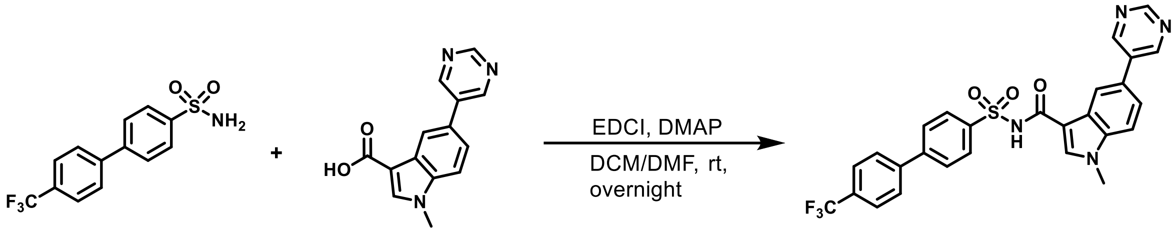

SA16 **CA19** **SZ16TA19**

The synthesis was carried out according to general procedure **(A)** using carboxylic acid **CA19** and sulfonamide **SA16**. The reaction solvent was anhydrous DCM (0.05 M) and anhydrous DMF (0.12 M) and the mixture was stirred overnight at room temperature under an argon atmosphere. The crude material was purified by flash chromatography (DCM/MeOH) to yield pure **SZ16TA19** (30%). R_f = 0.26 in 5% MeOH/DCM.

^{1}H NMR (500 MHz, $(CD_3)_2SO$) δ 12.16 (s, 1H), 9.15 (s, 1H), 9.08 (s, 2H), 8.47 (s, 1H), 8.30 (s, 1H), 8.14 (d, *J* = 8.2 Hz, 2H), 8.00 (d, *J* = 8.1 Hz, 2H), 7.96 (d, *J* = 8.1 Hz, 2H), 7.86 (d, *J* = 8.1 Hz, 2H), 7.73 (d, *J* = 8.6 Hz, 1H), 7.68 (d, *J* = 8.4 Hz, 1H), 3.92 (s, 3H).

HRMS-ESI (*m*/*z*): $[M+H]^+$ calculated for $C_{27}H_{20}F_3N_4O_3S$, 537.1209 Da; found 537.1205 Da.

Synthesis of 1-(3,5-bis(trifluoromethyl)benzyl)-N-((4'-phenoxy-[1,1'-biphenyl]-4-yl)sulfonyl)-1H-indole-5-carboxamide (SZ35TA30)

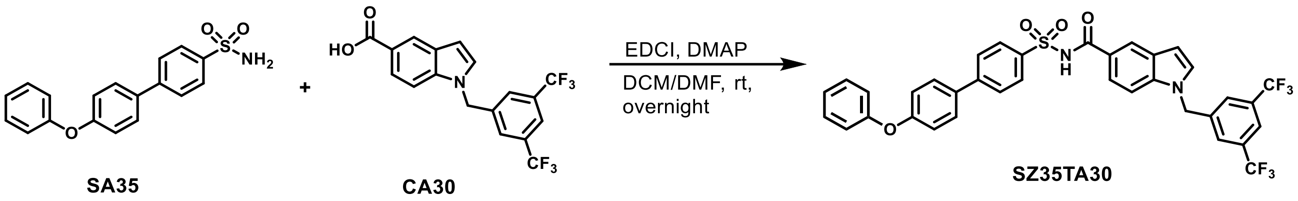

The synthesis was carried out according to general procedure **(A)** using carboxylic acid **CA30** and sulfonamide **SA35**. The reaction solvent was anhydrous DCM (0.05 M) and anhydrous DMF (0.12 M) and the mixture was stirred overnight at room temperature under an argon atmosphere. The crude material was purified by flash chromatography (DCM/MeOH) to yield pure **SZ35TA30** (17%). R_f = 0.3 in 3% MeOH/DCM.

^{1}H NMR (500 MHz, $(CD_3)_2SO$) δ 12.40 (s, 1H), 8.33 (s, 1H), 8.11 (s, 1H), 8.08 (d, *J* = 8.2 Hz, 2H), 7.97 – 7.88 (m, 3H), 7.78 (d, *J* = 8.5 Hz, 2H), 7.66 (dd, *J* = 8.8, 1.8 Hz, 1H), 7.63 (d, *J* = 3.2 Hz, 1H), 7.44 (t, *J* = 7.8 Hz, 2H), 7.36 (d, *J* = 8.8 Hz, 1H), 7.20 (t, *J* = 7.4 Hz, 1H), 7.11 (t, *J* = 7.9 Hz, 4H), 6.78 (d, *J* = 3.2 Hz, 1H), 6.58 (d, *J* = 8.2 Hz, 1H), 5.80 (s, 2H).

HRMS-ESI (*m*/*z*): $[M+H]^+$ calculated for $C_{36}H_{25}F_6N_2O_4S$, 695.1440 Da; found 695.1438 Da.

Synthesis of 3,5-dimethoxy-N-((4-((4-phenylpiperazin-1-yl)methyl)phenyl)sulfonyl)benzamide (SZ1TA5)

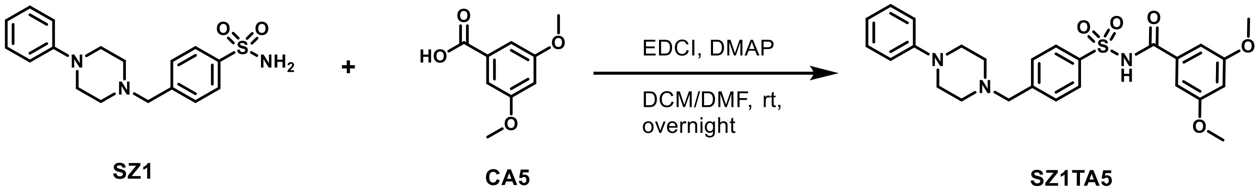

The synthesis was carried out according to general procedure **(A)** using carboxylic acid **TA5** and sulfonamide **SZ1**. The reaction solvent was anhydrous DCM (0.05 M) and anhydrous THF (0.12 M) and the mixture was stirred overnight at room temperature under an argon atmosphere. The crude material was purified by flash chromatography (DCM/MeOH) to yield pure **SZ1TA5** (27%). R_f = 0.24 in 5% MeOH/DCM.

^{1}H NMR (500 MHz, $(CD_3)_2SO$) δ 7.95 (d, *J* = 8.2 Hz, 2H), 7.57 (d, *J* = 8.0 Hz, 2H), 7.22 (t, *J* = 7.9 Hz, 2H), 7.06 (d, *J* = 2.3 Hz, 2H), 6.93 (d, *J* = 8.2 Hz, 2H), 6.80 (t, *J* = 7.3 Hz, 1H), 6.61 (s, 1H), 3.97 (s, 2H), 3.75 (s, 6H), 3.24 (s, 4H), 2.87 (s, 4H).

HRMS-ESI (*m*/*z*): $[M+H]^+$ calculated for $C_{26}H_{30}N_3O_5S$, 496.1907 Da; found 496.1904 Da.

Synthesis of 4-methyl-N-((4-((methyl(phenethyl)amino)methyl)phenyl)sulfonyl)-2-phenylthiazole-5-carboxamide (SZ2TA3)

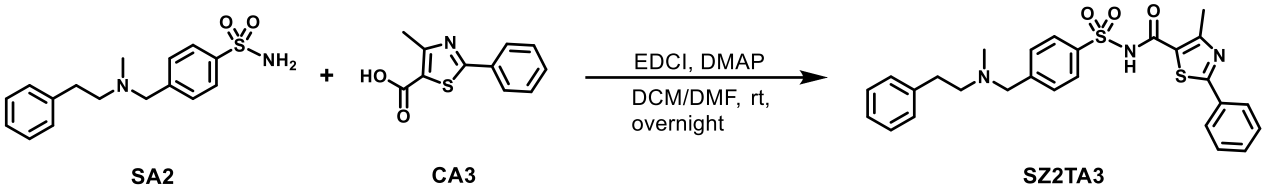

The synthesis was carried out according to general procedure **(A)** using carboxylic acid **CA3** and sulfonamide **SA2**. The reaction solvent was anhydrous DCM (0.05 M) and anhydrous DMF (0.12 M) and the mixture was stirred overnight at room temperature under an argon atmosphere. The crude material was purified by flash chromatography (5% MeOH/DCM with 0.1% NEt_3) to yield pure **SZ2TA3** (43%). R_f = 0.43 in 10% MeOH/DCM with 0.1% NEt_3.

^{1}H NMR (500 MHz, $(CD_3)_2SO$) δ 9.74 (s, 1H), 8.00 – 7.91 (m, 4H), 7.64 (d, *J* = 7.9 Hz, 2H), 7.55 – 7.48 (m, 3H), 7.38 (t, *J* = 7.6 Hz, 2H), 7.33 – 7.27 (m, 3H), 4.60 – 4.53 (m, 1H), 4.34 (dd, *J* = 12.9, 6.4 Hz, 1H), 3.36 – 3.24 (m, 2H), 3.12 (td, *J* = 12.4, 5.2 Hz, 1H), 3.03 (td, *J* = 12.3, 5.2 Hz, 1H), 2.81 (d, *J* = 4.6 Hz, 3H), 2.60 (s, 3H).

HRMS-ESI (*m*/*z*): $[M+H]^+$ calculated for $C_{27}H_{28}N_3O_3S_2$, 506.1573 Da; found 506.1571 Da.

Synthesis of 3,5-dimethoxy-N-((4-((methyl(phenethyl)amino)methyl)phenyl)sulfonyl)benzamide (SZ2TA5)

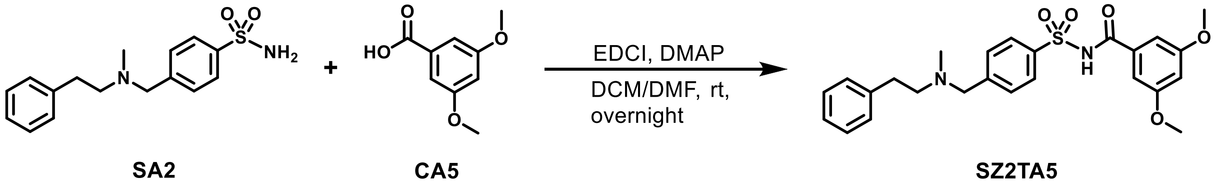

The synthesis was carried out according to general procedure **(A)** using carboxylic acid **CA5** and sulfonamide **SA2**. The reaction solvent was anhydrous DCM (0.05 M) and anhydrous DMF (0.12 M) and the mixture was stirred overnight at room temperature under an argon atmosphere. The crude material was purified by 1 M HCl wash to yield pure **SZ2TA5** (76%). R_f = 0.48 in 10% MeOH/DCM with 0.1% NEt_3.

^{1}H NMR (500 MHz, $(CD_3)_2SO$) δ 7.91 (d, J = 7.9 Hz, 2H), 7.54 (d, J = 7.8 Hz, 2H), 7.33 (t, J = 7.5 Hz, 2H), 7.25 (d, J = 7.4 Hz, 3H), 7.07 (s, 2H), 6.54 (s, 1H), 4.23 (s, 2H), 3.74 (s, 6H), 3.16 (bs, 2H), 2.98 (t, J = 8.3 Hz, 2H), 2.65 (s, 3H).

HRMS-ESI (m/z): $[M+H]^+$ calculated for $C_{25}H_{29}N_2O_5S$, 469.1800 Da; found 469.1796 Da.

Synthesis of 3,5-dimethoxy-N-((4'-phenoxy-[1,1'-biphenyl]-4-yl)sulfonyl)benzamide (SZ35TA5)

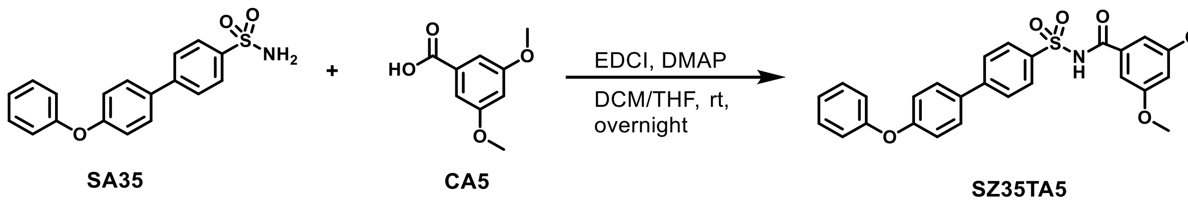

The synthesis was carried out according to general procedure **(A)** using carboxylic acid **CA5** and sulfonamide **SA35**. The reaction solvent was anhydrous DCM (0.04 M) and anhydrous THF (0.06 M) and the mixture was stirred overnight at room temperature under an argon atmosphere. The crude material was purified by preparative HPLC to yield pure **SZ35TA5** (7%). R_f = 0.33 in 5% MeOH/DCM.

^{1}H NMR (500 MHz, $(CD_3)_2CO$) δ 8.21 (d, J = 8.6 Hz, 2H), 7.93 (d, J = 8.6 Hz, 2H), 7.81 (d, J = 8.8 Hz, 2H), 7.47 (t, J = 8.0 Hz, 2H), 7.23 (t, J = 7.4 Hz, 2H), 7.17 – 7.13 (m, 5H), 6.73 (t, J = 2.3 Hz, 1H), 3.85 (s, 6H).

HRMS-ESI (m/z): $[M+H]^+$ calculated for $C_{27}H_{24}NO_6S$, 490.1325 Da; found 490.1324 Da.

Synthesis of 5-(4-ethoxy-3-fluorophenyl)-1-methyl-N-((4'-phenoxy-[1,1'-biphenyl]-4-yl)sulfonyl)-1H-indole-3-carboxamide (SZ35TA23)

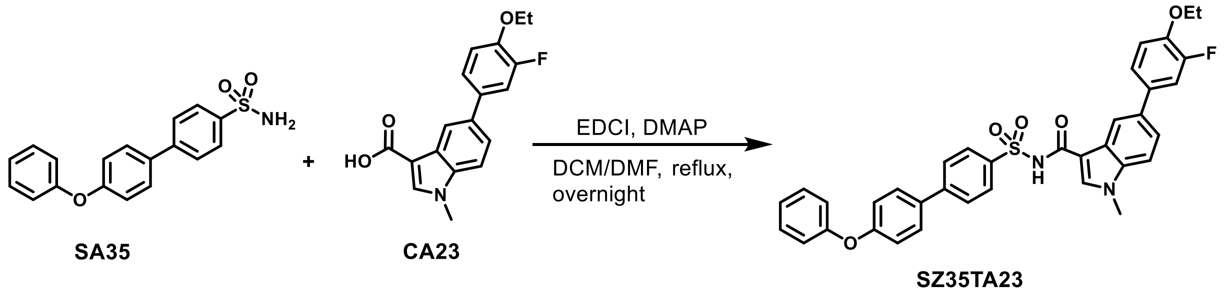

The synthesis was carried out according to general procedure **(A)** using carboxylic acid **CA23** and sulfonamide **SA35**. The reaction solvent was anhydrous DCM (0.05 M) and anhydrous DMF (0.05 M) and

the mixture was stirred overnight at room temperature under an argon atmosphere. The crude material was purified by flash chromatography to yield pure **SZ35TA23** (14%). R_f = 0.36 in 5% MeOH/DCM.

^{1}H NMR (500 MHz, $(CD_3)_2SO$) δ 12.01 (s, 1H), 8.39 (s, 1H), 8.16 (s, 1H), 8.06 (d, *J* = 7.2 Hz, 2H), 7.89 (d, *J* = 8.3 Hz, 2H), 7.75 (d, *J* = 7.1 Hz, 2H), 7.58 (d, *J* = 8.8 Hz, 1H), 7.51 (d, *J* = 8.8 Hz, 1H), 7.48 – 7.33 (m, 4H), 7.23 – 7.12 (m, 2H), 7.12 – 6.96 (m, 4H), 4.11 (q, *J* = 7.6 Hz, 2H), 3.86 (s, 3H), 1.43 – 1.24 (m, 3H).

HRMS-ESI (*m*/*z*): $[M+H]^+$ calculated for $C_{36}H_{30}FN_2O_5S$, 621.1860 Da; found 621.1866 Da.

Synthesis of N-((2-(6,7-dimethoxy-1-phenyl-3,4-dihydroisoquinolin-2(1H)-yl)ethyl)sulfonyl)-4-(4,4-dimethylpiperidin-1-yl)benzamide (SZ27TA2)

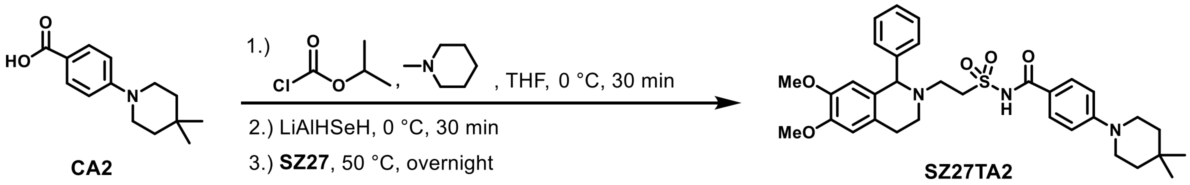

The synthesis was carried out according to general procedure **(B)** using carboxylic acid **CA2** and sulfonyl azide **SZ27**. The final reaction mixture was stirred overnight at 50 ºC. After work-up, the crude material was purified by preparative HPLC to afford the pure product **SZ27TA2** (36%). R_f = 0.27 in 4% MeOH/DCM.

^{1}H NMR (500 MHz, $CDCl_3$) δ 7.58 (d, *J* = 8.7 Hz, 2H), 7.30 – 7.15 (m, 5H), 6.73 (d, *J* = 9.0 Hz, 2H), 6.55 (s, 1H), 6.18 (s, 1H), 4.77 (s, 1H), 3.84 (s, 3H), 3.80 – 3.64 (m, 2H), 3.61 (s, 3H), 3.32 (t, *J* = 5.8 Hz, 4H), 3.23 – 3.03 (m, 3H), 2.95 – 2.72 (m, 3H), 1.49 (t, *J* = 5.8 Hz, 4H), 1.01 (s, 6H).

HRMS-ESI (*m*/*z*): $[M+H]^+$ calculated for $C_{33}H_{42}N_3O_5S$, 592.2846 Da; found 592.2852 Da.

Synthesis of N-((4-chlorophenyl)sulfonyl)-5-(4-ethoxy-3-fluorophenyl)-1-methyl-1H-indole-3-carboxamide (SZ18TA23)

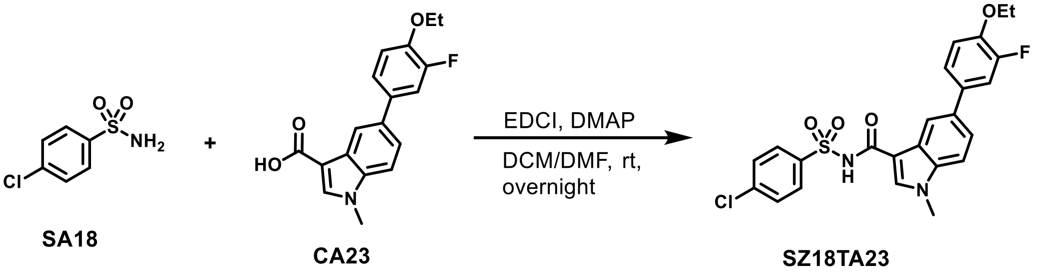

The synthesis was carried out according to general procedure **(A)** using carboxylic acid **CA23** and sulfonamide **SA18**. The reaction solvent was anhydrous DCM (0.05 M) and anhydrous DMF (0.05 M) and the mixture was stirred overnight at room temperature under an argon atmosphere. The crude material was purified by flash chromatography to yield pure **SZ18TA23** (22%). R_f = 0.35 in 5% MeOH/DCM.

^{1}H NMR (500 MHz, $(CD_3)_2SO$) δ 12.08 (s, 1H), 8.38 (s, 1H), 8.16 (d, *J* = 1.8 Hz, 1H), 8.02 (d, *J* = 8.7 Hz, 2H), 7.72 (d, *J* = 8.7 Hz, 2H), 7.60 (d, *J* = 8.6 Hz, 1H), 7.53 (dd, *J* = 8.6, 1.9 Hz, 1H), 7.46 (dd, *J* = 12.9, 2.2 Hz, 1H), 7.38 (d, *J* = 8.6 Hz, 1H), 7.20 (t, *J* = 8.8 Hz, 1H), 4.13 (q, *J* = 7.0 Hz, 2H), 3.87 (s, 3H), 1.36 (t, *J* = 7.0 Hz, 3H).

HRMS-ESI (*m*/*z*): $[M+H]^+$ calculated for $C_{24}H_{21}ClFN_2O_4S$, 487.0895 Da; found 487.0897 Da.

Synthesis of N-((2-(3,4-dihydroquinolin-1(2H)-yl)ethyl)sulfonyl)-4'-fluoro-[1,1'-biphenyl]-4-carboxamide (SZ23TA14)

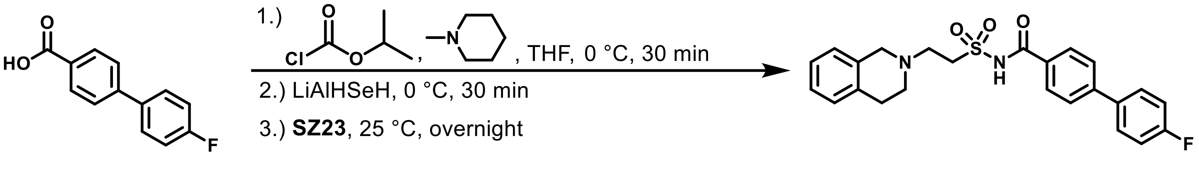

CA14 **SZ23TA14**

The synthesis was carried out according to general procedure **(B)** using carboxylic acid **CA14** and sulfonyl azide **SZ23**. The final reaction mixture was stirred overnight at room temperature. The crude product was purified by silica gel chromatography with 3% MeOH/DCM to yield **SZ23TA14** (61%). R_f = 0.49 in 8% MeOH/DCM.

^{1}H NMR (500 MHz, $(CD_3)_2SO$) δ 7.98 (d, *J* = 8.3 Hz, 2H), 7.75 (dd, *J* = 8.7, 5.6 Hz, 2H), 7.63 (d, *J* = 8.4 Hz, 2H), 7.31 (t, *J* = 8.8 Hz, 2H), 7.27 – 7.21 (m, 2H), 7.21 – 7.12 (m, 2H), 4.30 (s, 2H), 3.70 (t, *J* = 7.2 Hz, 2H), 3.43 – 3.37 (m, 4H), 3.00 (t, *J* = 6.3 Hz, 2H).

HRMS-ESI (m/z): $[M+H]^+$ calculated for $C_{24}H_{24}FN_2O_3S$, 439.1492 Da; found 439.1477 Da.

Synthesis of 4'-(N-(3,5-dimethoxybenzoyl)sulfamoyl)-N,N-dimethyl-[1,1'-biphenyl]-3-carboxamide(SZ34TA5)

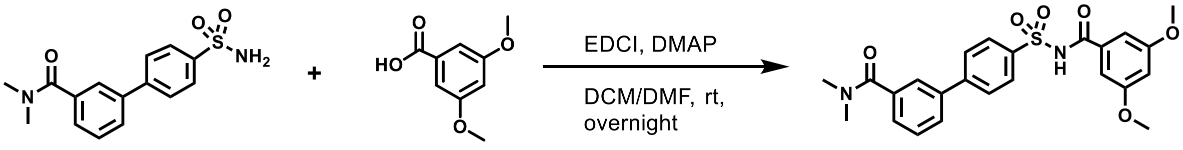

SZ34 **CA5** **SZ34TA5**

The synthesis was carried out according to general procedure **(A)** using carboxylic acid **CA5** and sulfonamide **SZ34**. The reaction solvent was anhydrous DCM (0.05 M) and anhydrous THF (0.12 M) and the mixture was stirred overnight at room temperature under an argon atmosphere. The crude material was purified by flash chromatography (DCM/MeOH) to yield pure **SZ34TA5** (4%). R_f = 0.20 in 5% MeOH/DCM.

^{1}H NMR (500 MHz, $CDCl_3$) δ 8.16 (d, *J* = 8.0 Hz, 2H), 7.69 (d, *J* = 5.7 Hz, 2H), 7.64 (s, 1H), 7.62 (d, *J* = 7.8 Hz, 1H), 7.49 (t, *J* = 7.7 Hz, 1H), 7.42 (d, *J* = 7.5 Hz, 1H), 7.03 – 6.92 (m, 2H), 6.59 (s, 1H), 3.76 (d, *J* = 3.6 Hz, 6H), 3.17 (s, 3H), 3.02 (s, 3H).

HRMS-ESI (*m*/*z*): $[M+H]^+$ calculated for $C_{24}H_{25}N_2O_6S$, 469.1434 Da; found 469.1432 Da.

Synthesis of 3,5-dimethoxy-N-((4-((4-phenylpiperazin-1-yl)methyl)phenyl)sulfonyl)benzamide (SZ25TA44)

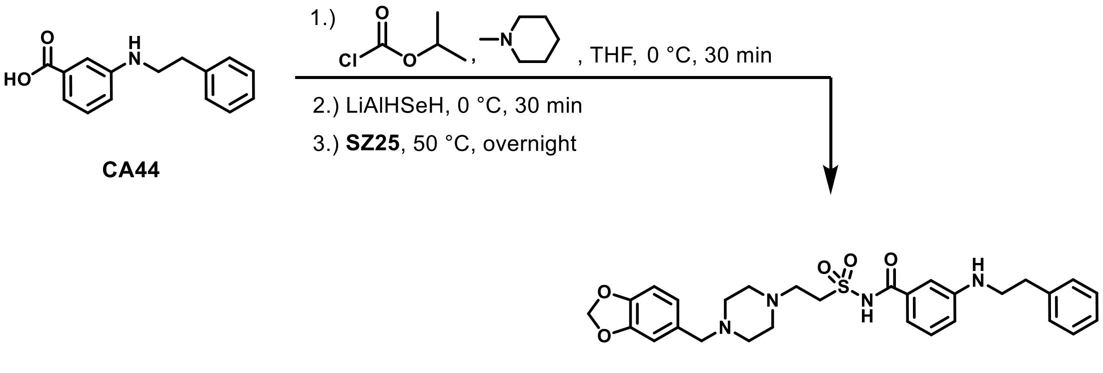

CA44

SZ25TA44

The synthesis was carried out according to general procedure **(B)** using carboxylic acid **CA44** and sulfonyl azide **SZ25**. The final reaction mixture was stirred overnight at 50 ºC. After work-up, the crude material was purified by preparative HPLC to afford the pure product **SZ25TA44** (30%). R_f = 0.41 in 10% MeOH/DCM.

^{1}H NMR (500 MHz, $CDCl_3$) δ 7.36 (t, *J* = 2.0 Hz, 1H), 7.34 – 7.27 (m, 3H), 7.25 – 7.15 (m, 4H), 6.75 – 6.69 (m, 2H), 6.67 (d, *J* = 7.9 Hz, 1H), 6.59 (d, *J* = 7.9 Hz, 1H), 5.90 (s, 2H), 5.63 (s, 2H), 3.68 (t, *J* = 7.3 Hz, 2H), 3.42 – 3.31 (m, 6H), 3.00 (s, 3H), 2.88 (t, *J* = 7.0 Hz, 2H), 2.62 (s, 3H).

HRMS-ESI (*m*/*z*): $[M+H]^+$ calculated for $C_{29}H_{35}N_4O_5S$, 551.2329 Da; found 551.2329 Da.

Synthesis of 4'-fluoro-N-((2-(octahydroquinolin-1(2H)-yl)ethyl)sulfonyl)-[1,1'-biphenyl]-4-carboxamide (SZ22TA14)

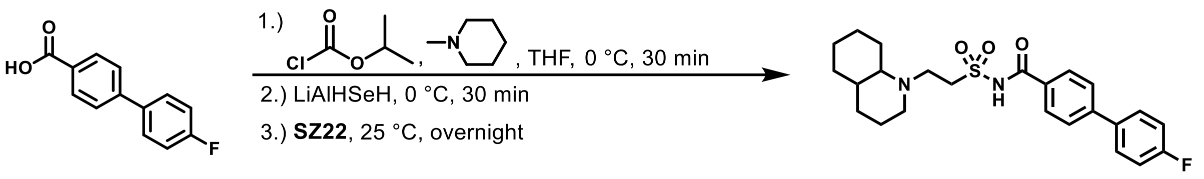

The synthesis was carried out according to general procedure **(B)** using carboxylic acid **CA14** and sulfonyl azide **SZ22**. The final reaction mixture was stirred overnight at room temperature. The crude product was purified by silica gel chromatography with 3% MeOH/DCM 0.1% NEt_3 to yield **SZ22TA14** (9%).
R_f = 0.56 in 10% MeOH/DCM, 0.1% NEt_3.
^{1}H NMR (500 MHz, $CDCl_3$) δ 8.05 (d, *J* = 7.9 Hz, 2H), 7.35 – 7.27 (m, 4H), 6.96 (t, *J* = 8.4 Hz, 2H), 3.99 (bs, 1H), 3.45 – 3.36 (m, 2H), 3.32 – 3.26 (m, 1H), 3.19 – 3.10 (m, 1H), 2.91 – 2.83 (m, 1H), 2.18 (m, 1H), 2.01 – 1.95 (m, 1H), 1.80 – 1.71 (m, 1H), 1.49 – 1.36 (m, 5H), 1.27 – 1.24 (m, 1H), 1.14 – 1.09 (m, 1H), 1.04 – 1.00 (m, 2H), 0.85 – 0.75 (m, 2H).
HRMS-ESI (*m*/*z*): $[M+H]^+$ calculated for $C_{24}H_{30}FN_2O_3S$, 445.1962 Da; found 445.1958 Da.

Synthesis of tert-butyl (3-(((4-acetamidophenyl)sulfonyl)carbamoyl)phenyl)(phenethyl)carbamate (SZ3TA44)

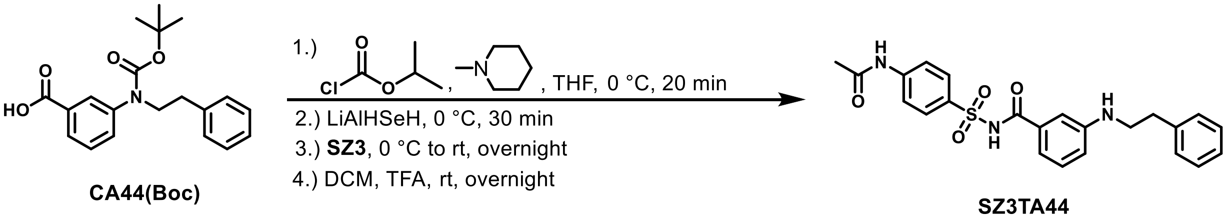

The synthesis was carried out according to general procedure **(B)** using Boc-protected carboxylic acid **CA44** and sulfonyl azide **SZ3**. The final reaction mixture was stirred overnight at 0 ºC to room temperature. The crude material was purified by flash chromatography (DCM/MeOH) to yield pure **SZ3TA44(Boc)** (42%). R_f = 0.31 in 3% MeOH/DCM.
^{1}H NMR (500 MHz, $CDCl_3$) δ 8.36 (s, 1H), 7.89 (d, *J* = 8.5 Hz, 2H), 7.59 – 7.53 (m, 3H), 7.47 (s, 1H), 7.33 – 7.07 (m, 6H), 3.84 (t, *J* = 7.4 Hz, 2H), 2.83 (t, *J* = 7.4 Hz, 2H), 2.14 (s, 3H), 1.45 (s, 9H).
SZ3TA44(Boc) was dissolved in DCM (0.10 M) then TFA (40 eq) was added and the reaction mixture was left to stir overnight at room temperature. The mixture was then diluted with saturated $NaHCO_3$ and extracted with EtOAc. The combined organic phases were dried over anhydrous Na_2SO_4 then concentrated *in vacuo*. The crude material was purified by flash chromatography (DCM/MeOH) to yield pure **SZ3TA44** (87%). R_f = 0.35 in 4% MeOH/DCM.

^{1}H NMR (500 MHz, CD_3OD) δ 8.01 (d, *J* = 9.0 Hz, 1H), 7.78 (d, *J* = 9.3 Hz, 2H), 7.37 (d, *J* = 4.7 Hz, 2H), 7.35 – 7.31 (m, 1H), 7.31 – 7.14 (m, 5H), 3.46 (t, *J* = 7.5 Hz, 2H), 2.96 – 2.89 (m, 2H), 2.15 (s, 1H).

HRMS-ESI (*m*/*z*): $[M+H]^+$ calculated for $C_{23}H_{24}N_3O_4S$ 438.1488 Da; found 438.1486 Da.

Synthesis of tert-butyl (3-(((2-(octahydroquinolin-1(2H)-yl)ethyl)sulfonyl)carbamoyl)phenyl)(phenethyl)carbamate (SZ22TA44)

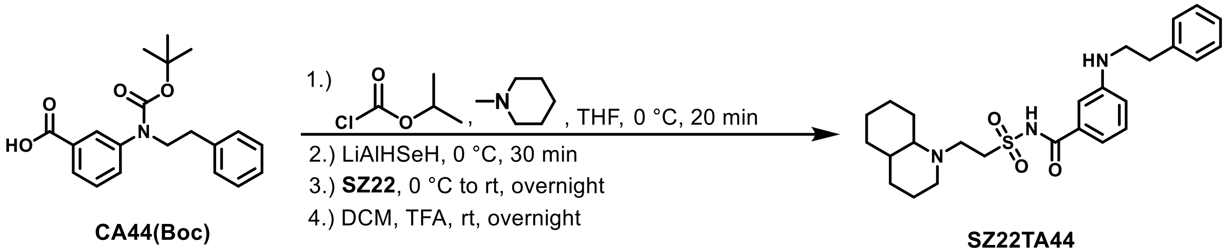

CA44(Boc) **SZ22TA44**

The synthesis was carried out according to general procedure **(B)** using Boc-protected carboxylic acid **CA44** and sulfonyl azide **SZ22**. The final reaction mixture was stirred overnight at 0 ºC to room temperature overnight under an argon atmosphere. The crude material was purified by flash chromatography (DCM/MeOH) to yield pure **SZ22TA44(Boc)** (44%). R_f = 0.28 in 2% MeOH/DCM.

^{1}H NMR (500 MHz, $CDCl_3$) δ 8.00 (d, *J* = 8.4 Hz, 2H), 7.33 – 7.13 (m, 7H), 3.97 – 3.61 (m, 6H), 3.42 – 3.33 (m, 1H), 2.90 – 2.77 (m, 3H), 2.63 (t, *J* = 10.7 Hz, 1H), 2.21 (d, *J* = 12.0 Hz, 1H), 2.06 – 1.59 (m, 8H), 1.41 (s, 9H), 1.31 – 1.04 (m, 4H).

SZ22TA44(Boc) was dissolved in DCM (0.10 M) then TFA (40 eq) was added and the reaction mixture was left to stir overnight at room temperature. The mixture was then diluted with saturated $NaHCO_3$ and extracted with EtOAc. The combined organic phases were dried over anhydrous Na_2SO_4 then concentrated *in vacuo*. The crude material was purified by flash chromatography (DCM/MeOH) to yield pure **SZ22TA44** (80%). R_f = 0.27 in 2% MeOH/DCM.

^{1}H NMR (500 MHz, CD_3OD) δ 7.36 – 7.09 (m, 8H), 6.78 – 6.73 (m, 1H), 3.77 – 3.59 (m, 4H), 3.46 – 3.38 (m, 1H), 3.35 (t, *J* = 7.4 Hz, 2H), 3.02 (td, *J* = 12.2, 4.4 Hz, 1H), 2.91 – 2.80 (m, 3H), 2.28 – 2.19 (m, 1H), 1.89 – 1.78 (m, 3H), 1.74 – 1.60 (m, 3H), 1.52 (q, *J* = 14.9, 11.5, 3.5 Hz, 1H), 1.41 – 1.18 (m, 4H), 1.15 – 1.04 (m, 1H).

HRMS-ESI (*m*/*z*): $[M+H]^+$ calculated for $C_{26}H_{36}N_3O_3S$, 470.2478 Da; found 470.2477 Da.

Synthesis of N-((2-(dibenzylamino)ethyl)sulfonyl)-3-(phenethylamino)benzamide (SZ26TA44)

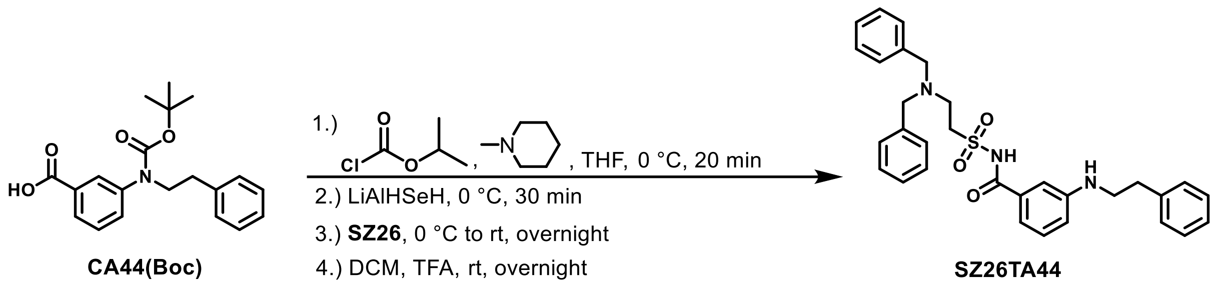

The synthesis was carried out according to general procedure **(B)** using Boc-protected carboxylic acid **CA44(Boc)** and sulfonyl azide **SZ26**. The final reaction mixture was stirred overnight at 0 ºC to room temperature overnight under an argon atmosphere. The crude material was purified by flash chromatography (DCM/MeOH) to yield pure **SZ26TA44(Boc)** (35%). R_f = 0.25 in 2% MeOH/DCM.

^{1}H NMR (500 MHz, $CDCl_3$): δ 7.39 – 7.33 (m, 1H), 7.18 – 6.82 (m, 18H), 3.68 – 3.56 (m, 2H), 3.50 – 3.34 (m, 6H), 2.89 – 2.79 (m, 2H), 2.67 – 2.58 (m, 2H), 1.19 (s, 9H).

SZ26TA44(Boc) was dissolved in DCM (0.10 M) then TFA (40 eq) was added and the reaction mixture was left to stir overnight at room temperature. The mixture was then diluted with saturated $NaHCO_3$ and extracted with EtOAc. The combined organic phases were dried over anhydrous Na_2SO_4 then concentrated *in vacuo*. The crude material was purified by flash chromatography (DCM/MeOH) to yield pure **SZ26TA44** (97%). R_f = 0.28 in 3% MeOH/DCM.

^{1}H NMR (500 MHz, CD_3OD) δ 7.38 – 7.33 (m, 4H), 7.31 – 7.17 (m, 12H), 7.08 (dd, *J* = 7.3, 1.4 Hz, 2H), 6.89 – 6.85 (m, 1H), 3.77 (s, 4H), 3.75 – 3.70 (m, 2H), 3.38 (t, *J* = 7.4 Hz, 2H), 3.08 (d, *J* = 7.6 Hz, 2H), 2.94 – 2.88 (m, 2H).

HRMS-ESI (*m/z*): $[M+H]^+$ calculated for $C_{31}H_{34}N_3O_3S$, 528.2322 Da; found 528.2325 Da.

Synthesis of N-((4-acetamidophenyl)sulfonyl)-2-(6,7-dimethoxy-1-phenyl-3,4-dihydroisoquinolin-2(1H)-yl)acetamide (SZ3TA4)

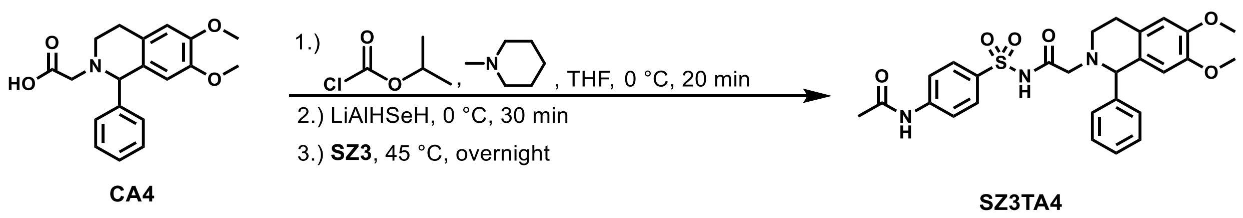

The synthesis was carried out according to general procedure **(B)** using carboxylic acid **CA4** and sulfonyl azide **SZ3**. The final reaction mixture was stirred overnight at 45 ºC under an argon atmosphere and the product was purified by flash chromatography (DCM/MeOH) yielding pure **SZ3TA4** (9%). R_f = 0.23 in 5% MeOH/DCM.

^{1}H NMR (500 MHz, $CDCl_3$) δ 7.88 (s, 1H), 7.86 (d, *J* = 8.9 Hz, 2H), 7.62 (d, *J* = 8.5 Hz, 2H), 7.37 – 7.27 (m, 3H), 7.23 – 7.15 (m, 2H), 6.64 (s, 1H), 6.09 (s, 1H), 3.87 (s, 3H), 3.58 (s, 3H), 3.18 (d, *J* = 17.0 Hz, 1H), 3.15 – 3.05 (m, 1H), 3.05 – 2.96 (m, 2H), 2.83 (dt, *J* = 16.3, 4.6 Hz, 1H), 2.78 – 2.69 (m, 1H), 2.19 (s, 3H).
HRMS-ESI (*m*/*z*): $[M+H]^+$ calculated for $C_{27}H_{30}N_3O_6S$, 524.1856 Da; found 524.1853 Da.

Synthesis of N-((2-(dibenzylamino)ethyl)sulfonyl)-3-(5-phenyl-1,3,4-oxadiazol-2-yl)propanamide (SZ26TA35)

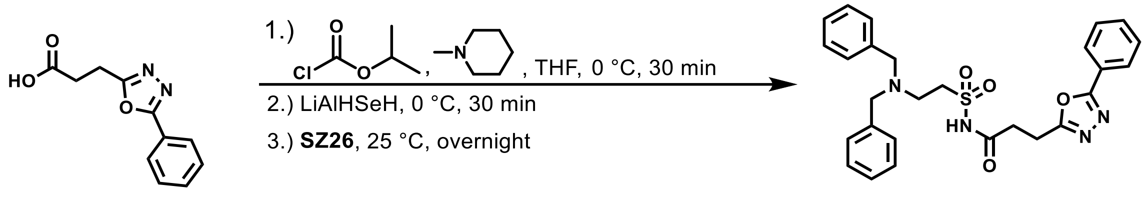

The synthesis was carried out according to general procedure **(B)** using carboxylic acid **CA35** and sulfonyl azide **SZ26**. The final reaction mixture was stirred overnight at room temperature. The crude product was purified by silica gel chromatography with 3% MeOH/DCM to yield **SZ26TA35** (88%).
R_f = 0.49 in 8% MeOH/DCM.
^{1}H NMR (500 MHz, $(CD_3)_2SO$) δ 7.97 (d, *J* = 6.9 Hz, 2H), 7.66 – 7.55 (m, 3H), 7.34 – 7.30 (m, 8H), 7.27 – 7.19 (m, 2H), 3.59 (t, *J* = 7.5 Hz, 2H), 3.54 (s, 4H), 3.12 (t, *J* = 7.0 Hz, 2H), 2.79 (t, *J* = 7.5 Hz, 2H), 2.74 (t, *J* = 7.0 Hz, 2H).
HRMS-ESI (*m*/*z*): $[M+H]^+$ calculated for $C_{27}H_{29}N_4O_4S$, 505.1910 Da; found 505.1908 Da.

Synthesis of N-((4-(2,3-dihydrobenzo[b][1,4]dioxin-6-yl)phenyl)sulfonyl)-2'-methoxy-[1,1'-biphenyl]-4-carboxamide (SZ33TA21)

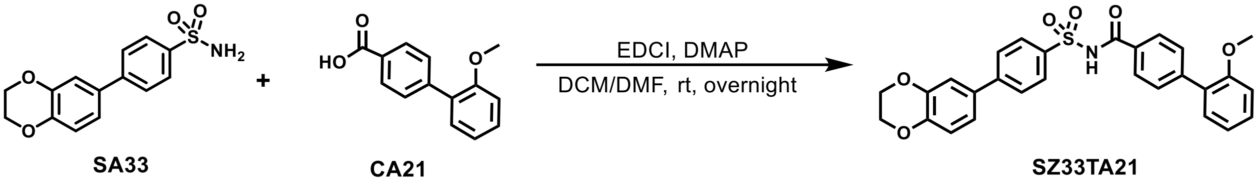

The synthesis was carried out according to general procedure **(A)** using carboxylic acid **CA21** and sulfonamide **SA33**. The reaction solvent was anhydrous DCM (0.05 M) and anhydrous DMF (0.12 M) and the mixture was stirred overnight at room temperature under an argon atmosphere. The crude material was

purified by flash chromatography (DCM/MeOH) to yield pure **SZ33TA21** (32%). R_f = 0.31 in 5% MeOH/DCM.

^{1}H NMR (500 MHz, $(CD_3)_2SO$) δ 12.55 (s, 1H), 8.01 (d, *J* = 8.1 Hz, 2H), 7.88 (dd, *J* = 17.2, 8.1 Hz, 4H), 7.58 (d, *J* = 8.0 Hz, 2H), 7.37 (t, *J* = 7.9 Hz, 1H), 7.34 – 7.19 (m, 3H), 7.12 (d, *J* = 8.3 Hz, 1H), 7.03 (t, *J* = 7.5 Hz, 1H), 6.97 (d, *J* = 8.4 Hz, 1H), 4.27 (s, 4H), 3.74 (s, 3H).

HRMS-ESI (*m*/*z*): $[M+H]^+$ calculated for $C_{28}H_{24}NO_6S$, 502.1325 Da; found 502.1328 Da.

Synthesis of N-((4-(benzo[b]thiophen-2-yl)phenyl)sulfonyl)-2'-methoxy-[1,1'-biphenyl]-4-carboxamide (SZ37TA21)

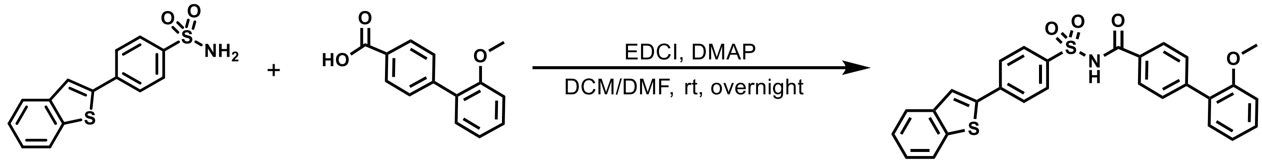

SA37 **CA21** **SZ37TA21**

The synthesis was carried out according to general procedure **(A)** using carboxylic acid **CA21** and sulfonamide **SA37**. The reaction solvent was anhydrous DCM (0.05 M) and anhydrous DMF (0.12 M) and the mixture was stirred overnight at room temperature under an argon atmosphere. The crude material was purified by flash chromatography (DCM/MeOH) to yield pure **SZ37TA21** (33%). R_f = 0.28 in 5% MeOH/DCM.

^{1}H NMR (500 MHz, $(CD_3)_2SO$) δ 8.09 – 7.96 (m, 6H), 7.90 (d, *J* = 7.7 Hz, 3H), 7.55 (d, *J* = 8.0 Hz, 2H), 7.45 – 7.33 (m, 3H), 7.29 (d, *J* = 7.5 Hz, 1H), 7.11 (d, *J* = 8.3 Hz, 1H), 7.02 (t, *J* = 7.5 Hz, 1H), 3.74 (s, 3H).

HRMS-ESI (*m*/*z*): $[M+H]^+$ calculated for $C_{28}H_{22}NO_4S_2$, 500.0991 Da; found 500.0994 Da.

Synthesis of N-((2-((benzo[d][1,3]dioxol-5-ylmethyl)((benzylthio)methyl)amino)ethyl)sulfonyl)-3-(cyclohexanecarbonyl)-1-methyl-1H-indole-6-carboxamide (SZ28TA40)

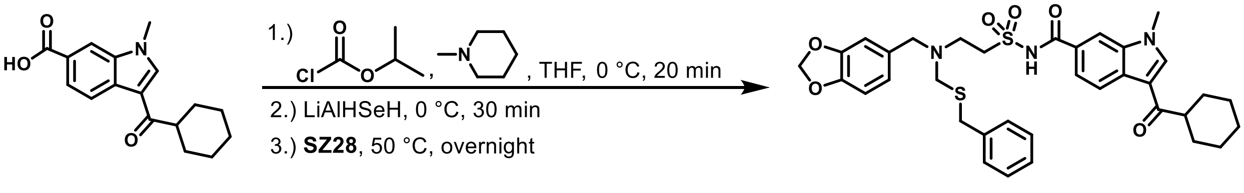

CA40 **SZ28TA40**

The synthesis was carried out according to general procedure **(B)** using carboxylic acid **CA40** and sulfonyl azide **SZ28**. The final reaction mixture was stirred overnight at 50 ºC under an argon atmosphere and the product was purified by flash chromatography (DCM/MeOH) yielding pure **SZ28TA40** (39%). R_f = 0.23 in 4% MeOH/DCM.

^{1}H NMR (500 MHz, $(CD_3)_2SO$) δ 8.86 (d, *J* = 1.8 Hz, 1H), 8.54 (s, 1H), 7.82 (dd, *J* = 8.7, 1.8 Hz, 1H), 7.62 (d, *J* = 8.7 Hz, 1H), 7.23 – 7.15 (m, 4H), 7.14 – 7.08 (m, 1H), 6.86 (s, 1H), 6.72 (s, 2H), 5.93 (s, 2H), 3.90 (s, 3H), 3.71 (t, *J* = 7.2 Hz, 2H), 3.57 (s, 2H), 3.20 – 3.11 (m, 1H), 3.03 (t, *J* = 7.3 Hz, 2H), 2.97 (t, *J* = 7.3 Hz, 2H), 2.67 (t, *J* = 7.4 Hz, 2H), 1.85 – 1.75 (m, 4H), 1.70 (d, *J* = 12.9 Hz, 1H), 1.51 – 1.35 (m, 4H), 1.18 (t, *J* = 7.1 Hz, 1H).

HRMS-ESI (*m*/*z*): $[M+H]^+$ calculated for $C_{35}H_{40}N_3O_6S_2$, 662.2359 Da; found 662.2354 Da.

Synthesis of 1-methyl-N-((4'-phenoxy-[1,1'-biphenyl]-4-yl)sulfonyl)-3-(thiophene-2-carbonyl)-1H-indole-5-carboxamide (SZ35TA45)

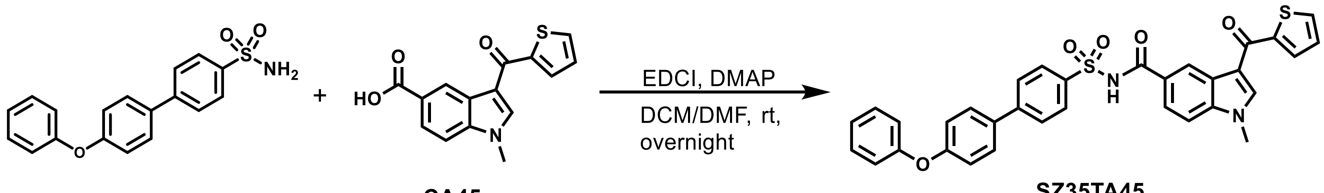

The synthesis was carried out according to general procedure **(A)** using carboxylic acid **CA45** and sulfonamide **SA35**. The reaction solvent was anhydrous DCM (0.05 M) and anhydrous DMF (0.12 M) and the mixture was stirred overnight at room temperature under an argon atmosphere. The crude material was purified by flash chromatography (DCM/MeOH) to yield pure **SZ35TA45** (9%). R_f = 0.28 in 4% MeOH/DCM.

^{1}H NMR (500 MHz, $(CD_3)_2CO$) δ 11.38 (s, 1H), 8.91 (s, 1H), 8.40 (s, 1H), 8.22 (d, *J* = 8.6 Hz, 2H), 7.99 – 7.94 (m, 1H), 7.94 – 7.88 (m, 3H), 7.88 (d, *J* = 5.0 Hz, 1H), 7.79 (d, *J* = 8.7 Hz, 2H), 7.64 (d, *J* = 8.7 Hz, 1H), 7.43 (t, *J* = 8.0 Hz, 2H), 7.29 – 7.22 (m, 1H), 7.19 (t, *J* = 7.4 Hz, 1H), 7.11 (dd, *J* = 11.6, 8.7 Hz, 4H), 4.04 (s, 3H).

HRMS-ESI (*m*/*z*): $[M+H]^+$ calculated for $C_{33}H_{25}N_2O_5S_2$, 593.1206 Da; found 593.1203 Da.

Synthesis of 1-(2,4-bis(trifluoromethyl)benzyl)-N-((4-(2,3-dihydrobenzo[b][1,4]dioxin-6-yl)phenyl)sulfonyl)-1H-indole-5-carboxamide (SZ33TA30)

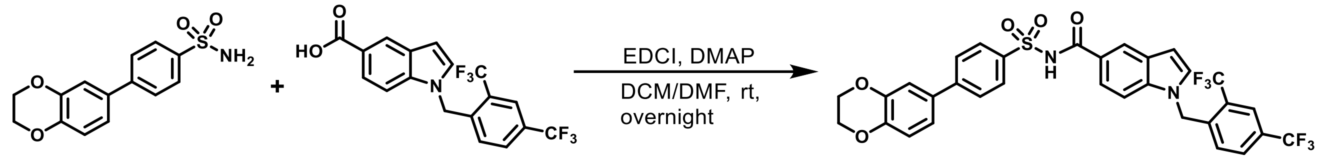

SA33 **CA30** **SZ33TA30**

The synthesis was carried out according to general procedure **(A)** using carboxylic acid **CA30** and sulfonamide **SA33**. The reaction solvent was anhydrous DCM (0.05 M) and anhydrous DMF (0.12 M) and the mixture was stirred overnight at room temperature under an argon atmosphere. The crude material was purified by flash chromatography (DCM/MeOH) to yield pure **SZ33TA30** (44%). R_f = 0.21 in 3% MeOH/DCM.

^{1}H NMR (500 MHz, $(CD_3)_2CO$) δ 8.38 (s, 1H), 8.16 (d, *J* = 8.6 Hz, 2H), 8.10 (s, 1H), 7.87 – 7.81 (m, 3H), 7.75 (dd, *J* = 8.8, 1.4 Hz, 1H), 7.58 (d, *J* = 3.2 Hz, 1H), 7.37 (d, *J* = 8.7 Hz, 1H), 7.26 – 7.17 (m, 2H), 6.95 (d, *J* = 9.0 Hz, 1H), 6.78 (d, *J* = 2.8 Hz, 1H), 6.72 (d, *J* = 8.2 Hz, 1H), 5.84 (s, 2H), 4.31 (s, 4H).

HRMS-ESI (*m/z*): $[M+H]^+$ calculated for $C_{32}H_{23}F_6N_2O_5S$, 661.1233 Da; found 661.1238 Da.

Synthesis of N-((2-(4-benzylpiperidin-1-yl)ethyl)sulfonyl)-2-(6,7-dimethoxy-1-phenyl-3,4-dihydroisoquinolin-2(1H)-yl)acetamide (SZ24TA4)

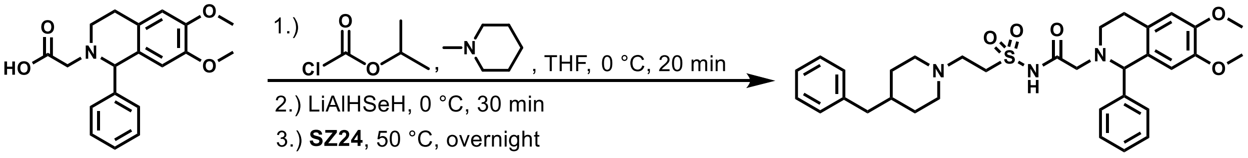

CA4 **SZ24TA4**

The synthesis was carried out according to general procedure **(B)** using carboxylic acid **CA4** and sulfonyl azide **SZ24**. The final reaction mixture was stirred overnight at 50 ºC under an argon atmosphere and the product was purified by flash chromatography (DCM/MeOH) yielding pure **SZ24TA4** (14%). R_f = 0.24 in 5% MeOH/DCM.

^{1}H NMR (500 MHz, $CDCl_3$) δ 7.38 – 7.22 (m, 7H), 7.19 (t, *J* = 7.3 Hz, 1H), 7.10 (d, *J* = 7.2 Hz, 2H), 6.62 (s, 1H), 6.15 (s, 1H), 3.86 (s, 3H), 3.60 (s, 3H), 3.57 – 3.44 (m, 2H), 3.30 (q, *J* = 16.6 Hz, 2H), 3.21 – 3.11 (m, 1H), 3.09 – 2.81 (m, 7H), 2.51 (d, *J* = 7.1 Hz, 2H), 2.15 (q, *J* = 11.0 Hz, 2H), 1.66 (d, *J* = 12.5 Hz, 2H), 1.61 – 1.52 (m, 1H), 1.43 – 1.28 (m, 2H), 1.25 (s, 1H).

HRMS-ESI (*m/z*): $[M+H]^+$ calculated for $C_{33}H_{42}N_3O_5S$, 592.2846 Da; found 592.2846 Da.

Synthesis of 2-phenyl-N-((4'-(trifluoromethyl)-[1,1'-biphenyl]-4-yl)sulfonyl)benzofuran-5-carboxamide (SZ16TA41)

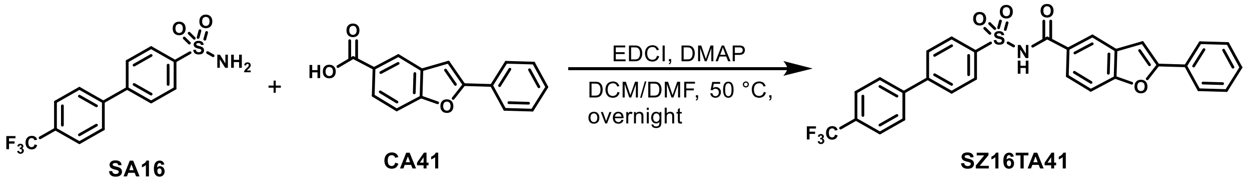

The synthesis was carried out according to general procedure **(A)** using carboxylic acid **CA41** and sulfonamide **SA16**. The reaction solvent was anhydrous DCM (0.05 M) and anhydrous DMF (0.13 M) and the mixture was stirred overnight at room temperature under an argon atmosphere. The crude material was suspended in methanol and the resulting precipitate was filtered off to afford pure **SZ16TA41** (15%). R_f = 0.28 in 5% MeOH/DCM.

^{1}H NMR (500 MHz, $(CD_3)_2SO$) δ 8.21 (s, 1H), 7.92 (m, 7H), 7.80 (d, *J* = 7.2 Hz, 2H), 7.74 (d, *J* = 6.8 Hz, 2H), 7.48 (m, 4H), 7.38 (t, *J* = 6.4 Hz, 1H).

HRMS-ESI (*m/z*): $[M+H]^+$ calculated for $C_{28}H_{19}F_3NO_4S$, 522.0988 Da; found 522.0989 Da.

Synthesis of N-((2-((benzo[d][1,3]dioxol-5-ylmethyl)((benzylthio)methyl)amino)ethyl)sulfonyl)-1-(2,4-bis(trifluoromethyl)benzyl)-1H-indole-5-carboxamide (SZ28TA30)

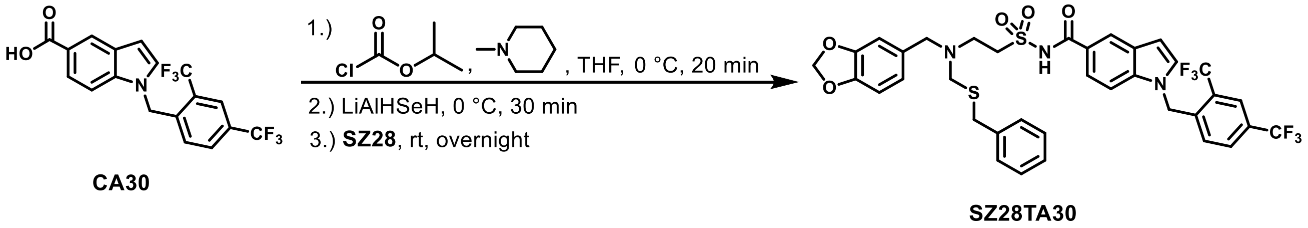

The synthesis was carried out according to general procedure **(B)** using carboxylic acid **CA30** and sulfonyl azide **SZ28**. The final reaction mixture was stirred overnight at room temperature under an argon atmosphere and the product was purified by flash chromatography (DCM/MeOH) yielding pure **SZ28TA30** (8%). R_f = 0.29 in 5% MeOH/DCM.

^{1}H NMR (500 MHz, CDCl3) δ 8.25 (s, 1H), 7.98 (s, 1H), 7.70 (d, *J* = 8.7 Hz, 1H), 7.65 – 7.54 (m, 1H), 7.22 (d, *J* = 3.1 Hz, 1H), 7.19 – 7.09 (m, 6H), 6.74 – 6.70 (m, 1H), 6.65 (s, 1H), 6.61 (d, *J* = 8.2 Hz, 1H), 6.52 (d, *J* = 7.8 Hz, 1H), 6.39 (d, *J* = 7.9 Hz, 1H), 5.82 (s, 2H), 5.61 (s, 2H), 3.74 – 3.69 (m, 2H), 3.49 (s, 2H), 3.07 (t, *J* = 6.3 Hz, 2H), 3.00 (t, *J* = 6.7 Hz, 2H), 2.67 (t, *J* = 6.7 Hz, 2H).

HRMS-ESI (*m/z*): $[M+H]^+$ calculated for $C_{36}H_{32}F_6N_3O_5S_2$, 764.1688 Da; found 764.1690 Da.

Synthesis of N-((2-(octahydroquinolin-1(2H)-yl)ethyl)sulfonyl)-2-phenylbenzofuran-5-carboxamide (SZ22TA41)

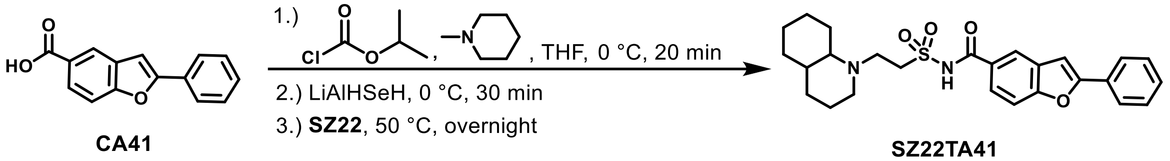

The synthesis was carried out according to general procedure **(B)** using carboxylic acid **CA41** and sulfonyl azide **SZ22**. The final reaction mixture was stirred overnight at 50 °C and the product was purified by flash chromatography (DCM/MeOH) yielding pure **SZ22TA41** (10%). R_f = 0.23 in 5% MeOH/DCM.

^{1}H NMR (500 MHz, $CDCl_3$) δ 8.44 (s, 1H), 8.13 (d, *J* = 8.6 Hz, 1H), 7.85 (d, *J* = 7.7 Hz, 2H), 7.51 – 7.40 (m, 3H), 7.35 (t, *J* = 7.4 Hz, 1H), 7.03 (s, 1H), 3.98 (d, *J* = 11.9 Hz, 1H), 3.88 – 3.75 (m, 1H), 3.73 (t, *J* = 5.7 Hz, 2H), 3.50 – 3.31 (m, 1H), 2.81 (t, *J* = 12.2 Hz, 1H), 2.59 (t, *J* = 10.0 Hz, 1H), 2.21 (d, *J* = 11.3 Hz, 1H), 2.05 (q, *J* = 14.0, 13.1 Hz, 1H), 1.92 (d, *J* = 12.3 Hz, 1H), 1.88 – 1.75 (m, 3H), 1.75 – 1.64 (m, 2H), 1.39 – 1.14 (m, 4H), 1.14 – 1.00 (m, 1H).

Synthesis of N-((4'-phenoxy-[1,1'-biphenyl]-4-yl)sulfonyl)-2-phenylbenzofuran-5-carboxamide (SZ35TA41)

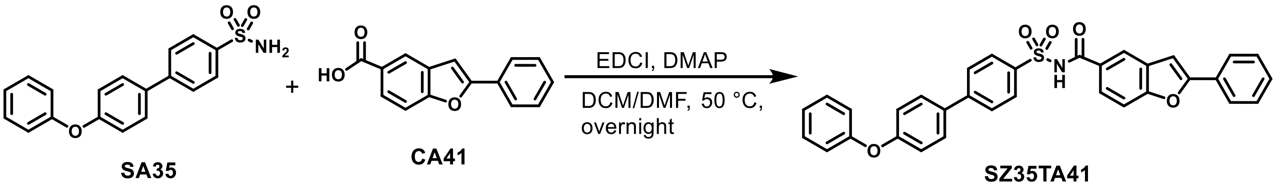

The synthesis was carried out according to general procedure **(A)** using carboxylic acid **CA41** and sulfonamide **SA35**. The reaction solvent was anhydrous DCM (0.05 M) and anhydrous DMF (0.13 M) and the mixture was stirred overnight at 50 °C under an argon atmosphere. The crude material was purified by flash chromatography (DCM/MeOH) to yield pure **SZ35TA41** (21%). R_f = 0.24 in 3% MeOH/DCM.

^{1}H NMR (500 MHz, $(CD_3)_2SO$) δ 12.60 (s, 1H), 8.28 (s, 1H), 8.08 (d, *J* = 8.0 Hz, 2H), 7.94 (dd, *J* = 15.1, 7.9 Hz, 4H), 7.85 (d, *J* = 8.7 Hz, 1H), 7.78 (d, *J* = 8.2 Hz, 2H), 7.73 (d, *J* = 8.6 Hz, 1H), 7.57 (s, 1H), 7.53 (t, *J* = 7.6 Hz, 2H), 7.44 (q, *J* = 7.5, 7.0 Hz, 3H), 7.19 (t, *J* = 7.5 Hz, 1H), 7.10 (t, *J* = 8.1 Hz, 4H).

HRMS-ESI (*m/z*): $[M+H]^+$ calculated for $C_{33}H_{24}NO_5S$, 546.1376 Da; found 546.1379 Da.

Synthesis of N-((4-methoxyphenyl)sulfonyl)-2-phenylbenzofuran-5-carboxamide (SZ19TA41)

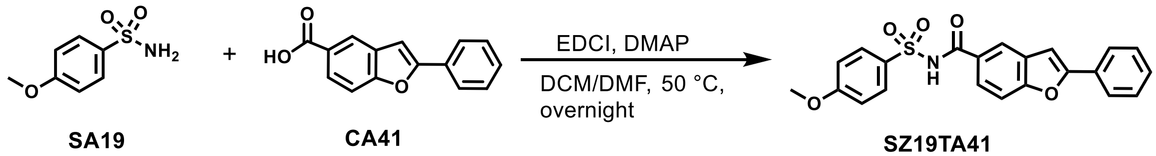

The synthesis was carried out according to general procedure **(A)** using carboxylic acid **CA41** and sulfonamide **SA19**. The reaction solvent was anhydrous DCM (0.05 M) and anhydrous DMF (0.13 M) and the mixture was stirred overnight at 50 ºC under an argon atmosphere. The crude material was purified by flash chromatography (DCM/MeOH) to yield pure **SZ19TA41** (40%). R_f = 0.3 in 4% MeOH/DCM

^{1}H NMR (500 MHz, $(CD_3)_2SO$) δ 12.42 (s, 1H), 8.24 (d, *J* = 1.9 Hz, 1H), 8.00 – 7.90 (m, 4H), 7.82 (dd, *J* = 8.7, 1.9 Hz, 1H), 7.72 (d, *J* = 8.7 Hz, 1H), 7.57 (s, 1H), 7.53 (dd, *J* = 8.5, 7.0 Hz, 2H), 7.48 – 7.42 (m, 1H), 7.21 – 7.12 (m, 2H), 3.86 (s, 3H).

HRMS-ESI (*m/z*): $[M+H]^+$ calculated for $C_{22}H_{18}NO_5S$, 408.0906 Da; found 408.0906 Da.

Synthesis of N-((4'-phenoxy-[1,1'-biphenyl]-4-yl)sulfonyl)-4-(2-(pyridin-2-yl)ethyl)benzamide (SZ35TA17)

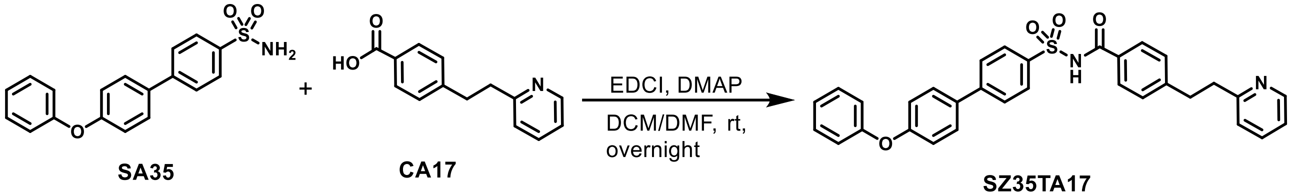

The synthesis was carried out according to general procedure **(A)** using carboxylic acid **CA17** and sulfonamide **SA35**. The reaction solvent was anhydrous DCM (0.05 M) and anhydrous DMF (0.13 M) and the mixture was stirred overnight at room temperature under an argon atmosphere. The crude material was purified by flash chromatography (DCM/MeOH) to yield pure **SZ35TA17** (57%). R_f = 0.28 in 5% MeOH/DCM.

^{1}H NMR (500 MHz, $CDCl_3$) δ 8.53 (d, *J* = 4.9 Hz, 1H), 8.19 (d, *J* = 8.2 Hz, 2H), 7.71 (dd, *J* = 13.3, 8.2 Hz, 4H), 7.54 (dd, *J* = 8.4, 3.9 Hz, 3H), 7.37 (t, *J* = 7.9 Hz, 2H), 7.21 – 7.09 (m, 4H), 7.07 (dd, *J* = 8.2, 5.4 Hz, 4H), 7.01 (d, *J* = 8.0 Hz, 1H), 3.11 – 2.97 (m, 4H).

HRMS-ESI (*m/z*): $[M+H]^+$ calculated for $C_{32}H_{27}N_2O_4S$, 535.1692 Da; found 535.1687 Da.

Synthesis of 1-methyl-N-((4-((2-nitrophenoxy)methyl)phenyl)sulfonyl)-3-(thiophene-2-carbonyl)-1H-indole-5-carboxamide (SZ13TA45)

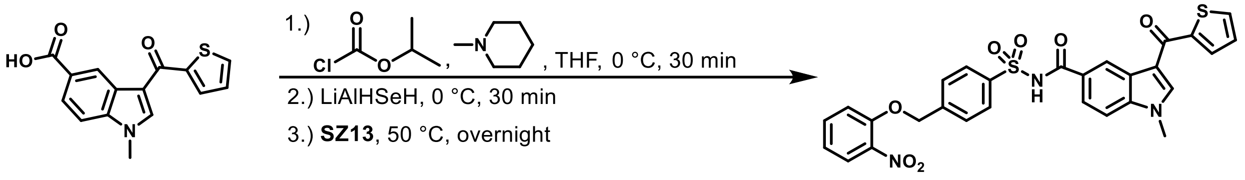

CA45 **SZ13TA45**

The synthesis was carried out according to general procedure **(B)** using carboxylic acid **CA45** and sulfonyl azide **SZ13**. The final reaction mixture was stirred overnight at 50 ºC. The crude material was purified by flash chromatography (DCM/MeOH) to yield pure **SZ13TA45** (38%). R_f = 0.2 in 4% MeOH/DCM.

^{1}H NMR (500 MHz, $(CD_3)_2SO$) δ 12.67 (s, 1H), 8.84 (d, *J* = 1.7 Hz, 1H), 8.55 (s, 1H), 8.11 – 8.05 (m, 2H), 8.00 (dq, *J* = 3.4, 1.2 Hz, 2H), 7.92 (dd, *J* = 8.0, 1.7 Hz, 1H), 7.81 (dd, *J* = 8.7, 1.8 Hz, 1H), 7.72 (d, *J* = 8.3 Hz, 2H), 7.68 (d, *J* = 8.6 Hz, 2H), 7.44 (d, *J* = 8.3 Hz, 1H), 7.30 (dd, *J* = 4.9, 3.8 Hz, 1H), 7.15 (t, *J* = 7.7 Hz, 1H), 5.45 (s, 2H), 3.95 (s, 3H).

HRMS-ESI (*m/z*): $[M+H]^+$ calculated for $C_{28}H_{22}N_3O_7S_2$, 576.0894 Da; found 576.0900 Da.

Synthesis N-((2-(4-(benzo[d][1,3]dioxol-5-ylmethyl)piperazin-1-yl)ethyl)sulfonyl)-1-(2,4-bis(trifluoromethyl)benzyl)-1H-indole-5-carboxamide (SZ25TA30)

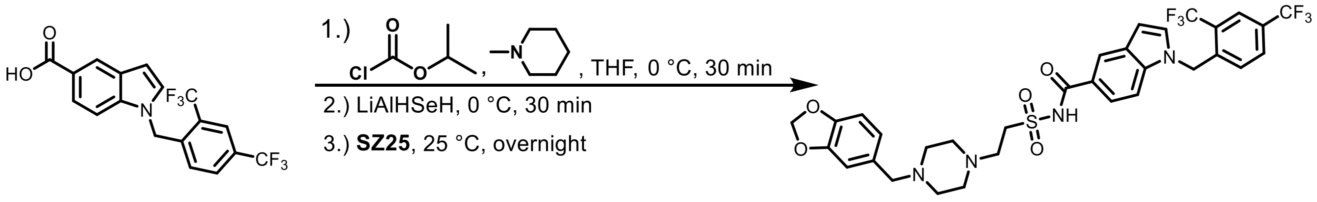

CA30 **SZ25TA30**

The synthesis was carried out according to general procedure **(B)** using carboxylic acid **CA30** and sulfonyl azide **SZ25**. The final reaction mixture was stirred overnight at room temperature. The crude product was purified by silica gel chromatography with 3% MeOH/DCM to yield **SZ25TA30** (59%). R_f = 0.21 in 5% MeOH/DCM.

^{1}H NMR (500 MHz, $CDCl_3$) δ 8.52 (s, 1H), 7.96 (s, 1H), 7.94 (dd, *J* = 8.7, 1.7 Hz, 1H), 7.49 (d, *J* = 8.3 Hz, 1H), 7.17 (d, *J* = 3.2 Hz, 1H), 7.10 (d, *J* = 8.7 Hz, 1H), 6.73 – 6.71 (m, 1H), 6.64 – 6.57 (m, 3H), 6.50 (d, *J* = 7.9 Hz, 1H), 5.89 (s, 2H), 5.61 (s, 2H), 3.76 (t, *J* = 7.4 Hz, 2H), 3.51 (t, *J* = 7.5 Hz, 2H), 3.16 (s, 3H), 3.14 – 3.06 (m, 2H), 2.73 – 2.39 (m, 6H).

HRMS-ESI (*m/z*): $[M+H]^+$ calculated for $C_{32}H_{31}F_6N_4O_5S$, 697.1920 Da; found 697.1917 Da.

Synthesis of 2'-methoxy-N-((4-(phenylsulfonyl)phenyl)sulfonyl)-[1,1'-biphenyl]-4-carboxamide (SZ38TA21)

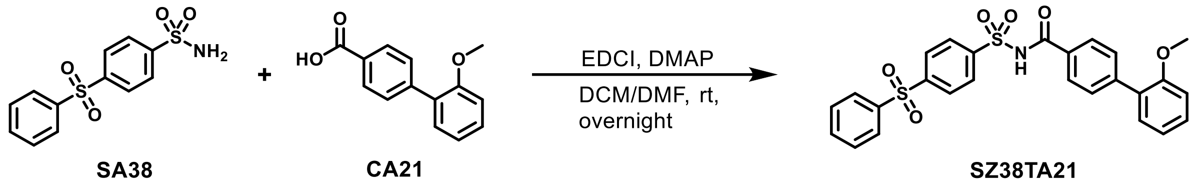

The synthesis was carried out according to general procedure **(A)** using carboxylic acid **CA21** and sulfonamide **SA38**. The reaction solvent was anhydrous DCM (0.05 M) and the crude material was purified by flash chromatography (2% MeOH/DCM) to yield pure **SZ38TA21** (5%). R_f = 0.38 in 5% MeOH/DCM.

^{1}H NMR (500 MHz, $CDCl_3$) δ 8.27 (d, J = 8.1 Hz, 2H), 8.09 (d, J = 8.0 Hz, 2H), 7.94 (d, J = 7.7 Hz, 2H), 7.78 (d, J = 8.0 Hz, 2H), 7.63 – 7.48 (m, 5H), 7.36 (t, J = 7.8 Hz, 1H), 7.26 (s, 1H), 7.06 – 6.95 (m, 2H), 3.78 (s, 3H).

HRMS-ESI (*m/z*): $[M+H]^+$ calculated for $C_{26}H_{22}NO_6S_2$, 508.0889 Da; found 508.0886 Da.

Synthesis of N,N-dimethyl-4'-(N-(4-(34yridine-2-yl)benzoyl)sulfamoyl)-[1,1'-biphenyl]-3-carboxamide (SZ33TA30)

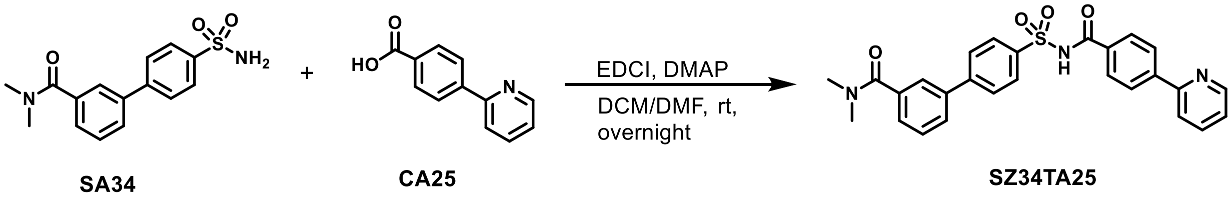

The synthesis was carried out according to general procedure **(A)** using carboxylic acid **CA25** and sulfonamide **SA34**. The reaction solvent was anhydrous DCM (0.05 M) and anhydrous DMF (0.13 M) and the mixture was stirred overnight at room temperature under an argon atmosphere. The crude material was purified by flash chromatography (DCM/MeOH) to yield pure **SZ34TA25** (31%). R_f = 0.35 in 5% MeOH/DCM.

^{1}H NMR (400 MHz, $CDCl_3$) δ 8.79 (d, J = 3.6 Hz, 1H), 8.15 (d, J = 8.1 Hz, 2H), 7.89 – 7.74 (m, 5H), 7.71 – 7.57 (m, 5H), 7.47 (t, J = 7.6 Hz, 1H), 7.41 (d, J = 7.5 Hz, 1H), 7.34 – 7.29 (m, 1H), 3.17 (s, 3H), 3.01 (s, 3H).

HRMS-ESI (*m/z*): $[M+H]^+$ calculated for $C_{27}H_{24}N_3O_4S$, 486.1488 Da; found 486.1488 Da.

Synthesis of N-((2-((benzo[d][1,3]dioxol-5-ylmethyl)((benzylthio)methyl)amino)ethyl)sulfonyl)-1-methyl-5-(4-(methylsulfonyl)phenyl)-1H-indole-3-carboxamide (SZ28TA26)

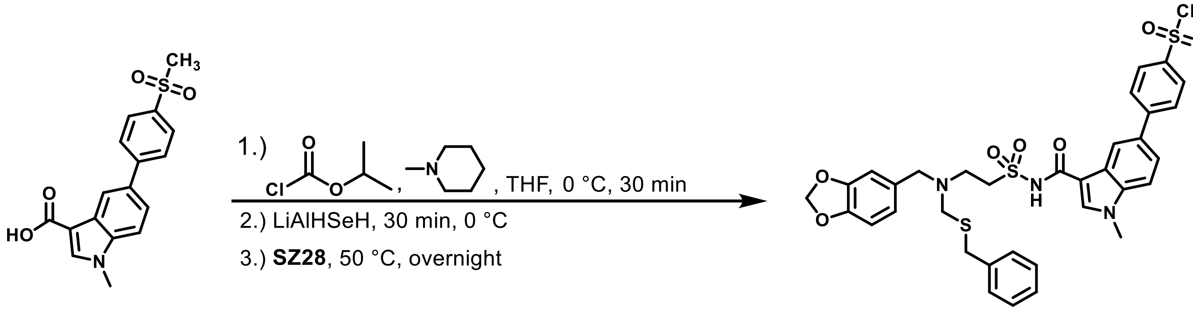

CA26 **SZ28TA26**

The synthesis was carried out according to general procedure **(B)** using carboxylic acid **CA26** and sulfonyl azide **SZ22**. The final reaction mixture was stirred overnight at 50 ºC. The product crashed out during the work-up procedure and thus was filtered off and washed with DCM in order to yield pure **SZ28TA26** (12%). R_f = 0.33 in 5% MeOH/DCM.

^{1}H NMR (500 MHz, $(CD_3)_2SO$) δ 8.63 (s, 1H), 7.93 (d, *J* = 8.4 Hz, 2H), 7.86 (d, *J* = 8.4 Hz, 2H), 7.70 (s, 1H), 7.49 (s, 2H), 7.18 – 7.10 (m, 4H), 7.09 – 7.00 (m, 1H), 6.83 (s, 1H), 6.70 (d, *J* = 8.0 Hz, 1H), 6.66 (d, *J* = 7.9 Hz, 1H), 5.90 (s, 2H), 3.78 (s, 3H), 3.45 (s, 2H), 3.28 – 3.22 (m, 2H), 3.19 (s, 3H), 2.99 (t, *J* = 7.2 Hz, 2H), 2.84 – 2.77 (m, 2H), 2.59 (t, *J* = 7.1 Hz, 2H).

HRMS-ESI (*m/z*): $[M+H]^+$ calculated for $C_{35}H_{36}N_3O_7S_3$, 706.1716 Da; found 706.1713 Da.

Synthesis of N-((2-((benzo[d][1,3]dioxol-5ylmethyl)((benzylthio)methyl)amino)ethyl)sulfonyl)-2-phenylbenzofuran-5-carboxamide (SZ28TA41)

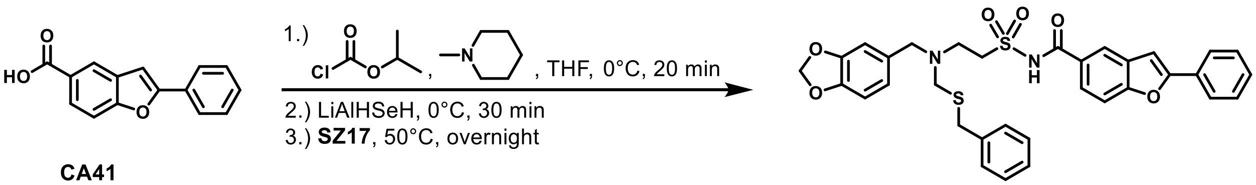

CA41 **SZ28TA41**

The synthesis was carried out according to general procedure **(B)** using carboxylic acid **CA41** and sulfonyl azide **SZ28**. The final reaction mixture was stirred overnight at 50 ºC and the product was purified by flash chromatography (DCM/MeOH) yielding pure **SZ28TA41** (9%). R_f = 0.31 in 2.5% MeOH/DCM.

^{1}H NMR (500 MHz, $CDCl_3$) δ 8.13 (s, 1H), 7.87 (d, J = 7.7 Hz, 2H), 7.82 (d, J = 8.5 Hz, 1H), 7.54 (d, J = 8.6 Hz, 1H), 7.48 (t, J = 7.6 Hz, 2H), 7.41 (t, J = 7.4 Hz, 1H), 7.21 (d, J = 4.3 Hz, 4H), 7.16 (q, J = 4.3 Hz, 1H), 6.99 (s, 1H), 6.62 (s, 1H), 6.55 (q, J = 7.9 Hz, 2H), 5.83 (s, 2H), 3.71 (t, J = 6.3 Hz, 2H), 3.54 (s, 2H), 3.11 (t, J = 6.3 Hz, 2H), 3.05 (t, J = 6.7 Hz, 2H), 2.71 (t, J = 6.7 Hz, 2H).
HRMS-ESI (*m/z*): $[M+H]^+$ calculated for $C_{33}H_{31}N_2O_6S_2$, 615.1624 Da; found 615.1620 Da.

Synthesis of 1-(1-(2,4-bis(trifluoromethyl)phenyl)ethyl)-N-(methylsuflonyl)-1H-indole-5-carboxamide (SZ(Me)TA30)

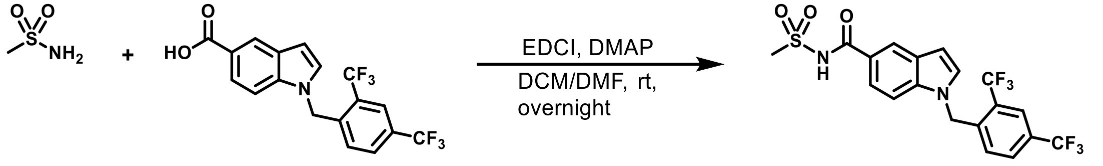

CA30 **SZ(Me)TA30**

The synthesis was carried out according to general procedure **(A)** using carboxylic acid **CA30** and methane sulfonamide. The reaction solvent was anhydrous DCM (0.05 M) and anhydrous DMF (0.13 M) and the mixture was stirred overnight at room temperature under an argon atmosphere. The crude material was purified by flash chromatography (DCM/MeOH) to yield pure **SZ(Me)TA30** (44%). R_f = 0.36 in 3% MeOH/DCM.
^{1}H NMR (400 MHz, $(CD_3)_2CO$) δ 10.52 (s, 1H), 8.43 (d, J = 0.97 Hz, 1H), 8.12 (s, 1H), 7.88 (d, J = 8.23 Hz, 1H), 7.83 (dd, J = 8.68, 1.55 Hz, 1H), 7.62 (d, J = 3.28 Hz, 1H), 7.41 (d, J = 8.70 Hz, 1H), 6.81 (d, J = 2.79 Hz, 1H), 6.74 (d, J = 8.25 Hz, 1H), 5.87 (s, 2H), 3.40 (s, 3H).
HRMS-ESI (*m/z*): $[M+H]^+$ calculated for $C_{19}H_{15}F_6N_2O_3S$, 465.0708 Da; found 465.0706 Da.

Synthesis of 2'-methoxy-N-(methylsulfonyl)-[1,1'-biphenyl]-4-carboxamide (SZ(Me)TA21)

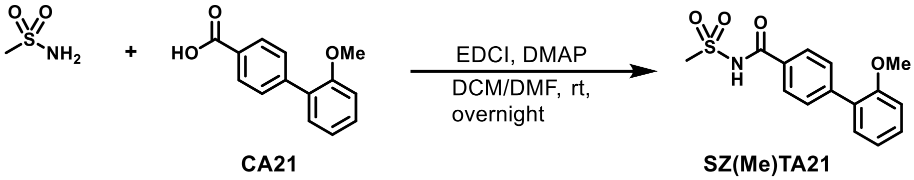

CA21 **SZ(Me)TA21**

The synthesis was carried out according to general procedure **(A)** using carboxylic acid **CA21** and methane sulfonamide. The reaction solvent was anhydrous DCM (0.05 M) and anhydrous DMF (0.13 M) and the mixture was stirred overnight at room temperature under an argon atmosphere. The crude material was

purified by flash chromatography (DCM/MeOH) to yield pure **SZ(Me)TA21** (4%). R_f = 0.25 in 4% MeOH/DCM.

^{1}H NMR (500 MHz, $(CD_3)_2SO$) δ 7.97 (d, *J* = 8.3 Hz, 2H), 7.58 (d, *J* = 8.2 Hz, 2H), 7.42 – 7.36 (m, 1H), 7.34 (dd, *J* = 7.5, 1.7 Hz, 1H), 7.14 (d, *J* = 8.2 Hz, 1H), 7.06 (t, *J* = 7.4 Hz, 1H), 3.78 (s, 3H), 3.26 (s, 3H).

HRMS-ESI (*m/z*): $[M+H]^+$ calculated for $C_{15}H_{16}NO_4S$, 306.0801 Da; found 306.0798 Da.

Synthesis of 1-methyl-N-(methylsulfonyl)-3-(thiophene-2-carbonyl)-1H-indole-5-carboxamide (SZ(Me)TA45)

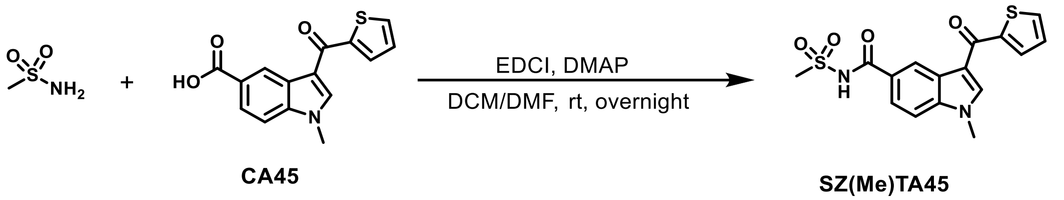

The synthesis was carried out according to general procedure **(A)** using carboxylic acid **CA45** and methane sulfonamide. The reaction solvent was anhydrous DCM (0.10 M) and anhydrous DMF (0.13 M) and the mixture was stirred overnight at room temperature under an argon atmosphere. The crude material was purified by flash chromatography (DCM/MeOH) to yield pure **SZ(Me)TA45** (11%). R_f = 0.23 in 4% MeOH/DCM.

^{1}H NMR (500 MHz, $(CD_3)_2SO$) δ 12.23 (s, 1H), 8.89 (d, *J* = 1.8 Hz, 1H), 8.56 (s, 1H), 8.05 – 7.97 (m, 2H), 7.90 (dd, *J* = 8.7, 1.8 Hz, 1H), 7.72 (d, *J* = 8.7 Hz, 1H), 7.31 (dd, *J* = 4.9, 3.7 Hz, 1H), 3.97 (s, 3H), 3.39 (s, 3H).

HRMS-ESI (*m/z*): $[M+H]^+$ calculated for $C_{16}H_{15}N_2O_4S_2$, 363.0474 Da; found 363.0470 Da.

Synthesis of N-(methylsulfonyl)-2-phenylbenzofuran-5-carboxamide (SZ(Me)TA41)

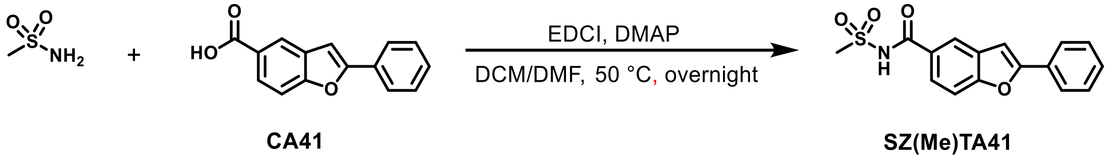

The synthesis was carried out according to general procedure **(A)** using carboxylic acid **CA41** and methane sulfonamide. The reaction solvent was anhydrous DCM (0.10 M) and anhydrous DMF (0.15 M) and the mixture was stirred overnight at room temperature under an argon atmosphere. The crude material was

purified by flash chromatography (DCM/MeOH) to yield pure **SZ(Me)TA41** (47%). R_f = 0.22 in 4% MeOH/DCM.
^{1}H NMR (500 MHz, $(CD_3)_2SO$) δ 12.15 (s, 1H), 8.32 (s, 1H), 7.97 (d, *J* = 7.7 Hz, 2H), 7.93 (d, *J* = 8.6 Hz, 1H), 7.76 (d, *J* = 8.6 Hz, 1H), 7.59 (s, 1H), 7.54 (t, *J* = 7.6 Hz, 2H), 7.45 (t, *J* = 7.5 Hz, 1H), 3.37 (s, 3H).
HRMS-ESI (*m/z*): $[M+H]^+$ calculated for $C_{16}H_{14}NO_4S$, 316.0644 Da; found 316.0644 Da.

Synthesis of 3,5-dimethoxy-N-(methylsulfonyl)benzamide (SZ(Me)TA5)

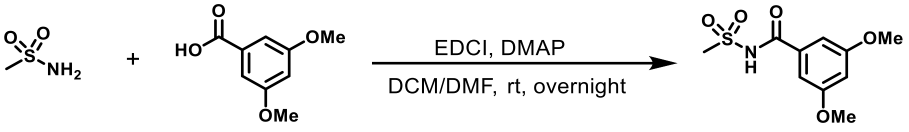

CA5 **SZ(Me)TA5**

The synthesis was carried out according to general procedure **(A)** using carboxylic acid **CA5** and methane sulfonamide. The reaction solvent was anhydrous DCM (0.10 M) and anhydrous DMF (0.15 M) and the mixture was stirred overnight at room temperature under an argon atmosphere. The crude material was purified by flash chromatography (DCM/MeOH) to yield pure **SZ(Me)TA5** (11%). R_f = 0.18 in 5% MeOH/DCM.
^{1}H NMR (500 MHz, $(CD_3)_2SO$) δ 7.12 (d, *J* = 2.3 Hz, 2H), 6.67 (s, 1H), 3.78 (s, 6H), 3.22 (s, 3H).
HRMS-ESI (*m/z*): $[M+H]^+$ calculated for $C_{10}H_{14}NO_5S$, 260.0593 Da; found 260.0590 Da.

Synthesis of N-((4-(2,3-dihydrobenzo[b][1,4]dioxin-6-yl)phenyl)sulfonyl)acetamide (SZ33TA(Me))

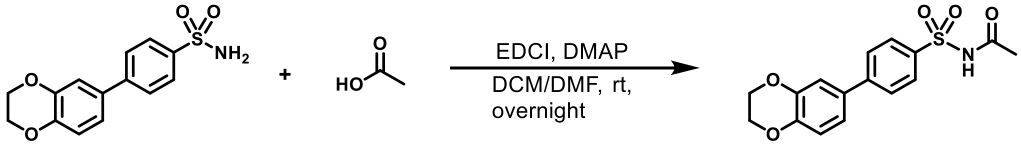

CA30 **SZ33TA(Me)**

The synthesis was carried out according to general procedure **(A)** using acetic acid and sulfonamide **SA33**. The reaction solvent was anhydrous DCM (0.05 M) and anhydrous DMF (0.13 M) and the mixture was stirred overnight at room temperature under an argon atmosphere. The crude material was purified by flash chromatography (DCM/MeOH) to yield pure **SZ33TA(Me)** (21%). R_f = 0.35 in 4% MeOH/DCM. ^{1}H NMR (400 MHz, $(CD_3)_2CO$) δ 10.66 (s, 1H), 8.04 (d, *J* = 8.49 Hz, 3H), 7.83 (d, *J* = 8.50 Hz, 2H), 7.24 (dd, *J* = 6.01, 2.00 Hz, 1H), 6.96 (d, *J* = 9.09 Hz, 1H), 4.33 (s, 4H), 2.04 (s, 3H).
HRMS-ESI (*m/z*): $[M+H]^+$ calculated for $C_{16}H_{16}NO_5S$, 334.0750 Da; found 334.0749 Da.

Synthesis of N-((4'-phenoxy-[1,1'-biphenyl]-4-yl)sulfonyl)acetamide (SZ35TA(Me))

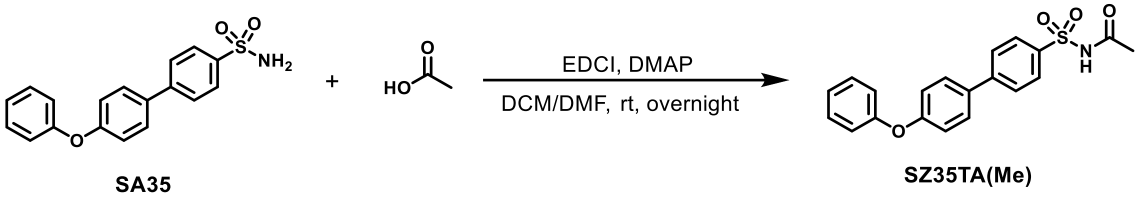

The synthesis was carried out according to general procedure **(A)** using acetic acid and sulfonamide **SA35**. The reaction solvent was anhydrous DCM (0.10 M) and anhydrous DMF (0.15 M) and the mixture was stirred overnight at room temperature under an argon atmosphere. The crude material was purified by flash chromatography (DCM/MeOH) to yield pure **SZ35TA(Me)** (60%). R_f = 0.24 (4% MeOH in DCM)

^{1}H NMR (500 MHz, $CDCl_3$) δ 8.10 (d, *J* = 8.4 Hz, 2H), 7.72 (d, *J* = 8.4 Hz, 2H), 7.57 (d, *J* = 8.6 Hz, 2H), 7.38 (t, *J* = 7.9 Hz, 2H), 7.16 (t, *J* = 7.4 Hz, 1H), 7.08 (t, *J* = 9.0 Hz, 4H), 2.10 (s, 3H).

HRMS-ESI (*m/z*): $[M+H]^+$ calculated for $C_{20}H_{18}NO_4S$, 368.0957 Da; found 334.0955 Da.

Synthesis of N-((4-(benzo[b]thiophen-2-yl)phenyl)sulfonyl)acetamide (SZ37TA(Me))

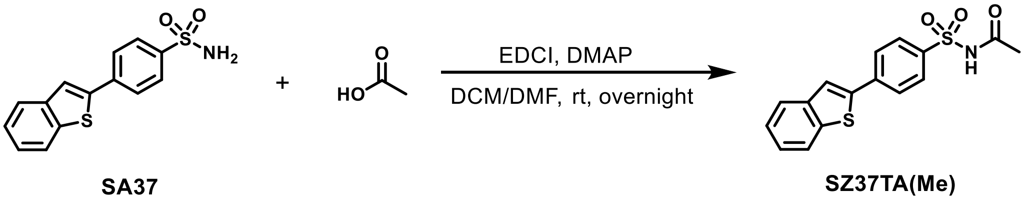

The synthesis was carried out according to general procedure **(A)** using acetic acid and sulfonamide **SA37**. The reaction solvent was anhydrous DCM (0.1 M) and anhydrous DMF (0.15 M) and the mixture was stirred overnight at room temperature under an argon atmosphere. The crude material was purified by flash chromatography (DCM/MeOH) to yield pure **SZ37TA(Me)** (38%). R_f = 0.23 in 4% MeOH/DCM.

^{1}H NMR (400 MHz, $(CD_3)_2SO$) δ 8.49 – 7.70 (m, 7H), 7.67 – 7.25 (m, 2H), 1.93 (s, 3H).

HRMS-ESI (*m/z*): $[M+H]^+$ calculated for $C_{16}H_{14}NO_3S_2$, 332.0416 Da; found 332.0404 Da.

Synthesis of N-((4'-(trifluoromethyl)-[1,1'-biphenyl]-4-yl)sulfonyl)acetamide (SZ16TA(Me))

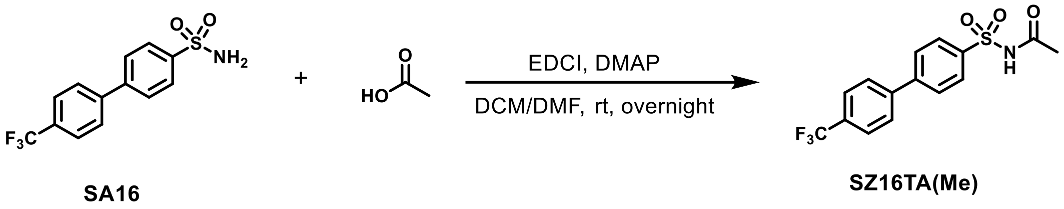

The synthesis was carried out according to general procedure **(A)** using acetic acid and sulfonamide **SA16**. The reaction solvent was anhydrous DCM (0.10 M) and anhydrous DMF (0.15 M) and the mixture was stirred overnight at room temperature under an argon atmosphere. The crude material was suspended in DCM and the resulting precipitate was filtered off to afford pure **SZ16TA(Me)** (28%). R_f = 0.15 in 5% MeOH/DCM.

^{1}H NMR (400 MHz, $(CD_3)_2CO$) δ 6.77 (d, *J* = 8.5 Hz, 2H), 6.60 (d, *J* = 8.2 Hz, 2H), 6.56 – 6.46 (m, 4H), 0.70 – 0.56 (m, 3H).

HRMS-ESI (*m/z*): $[M+H]^+$ calculated for $C_{15}H_{13}F_3NO_3S$, 344.0569 Da; found 344.0571 Da.

Synthesis of N-((4-((2-nitrophenoxy)methyl)phenyl)sulfonyl)acetamide (SZ13TA(Me))

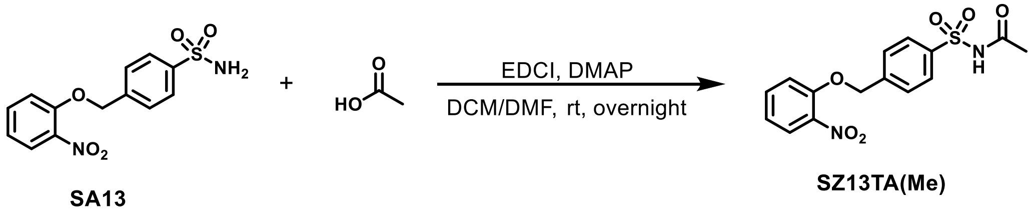

The synthesis was carried out according to general procedure **(A)** using acetic acid and sulfonamide **SA13**. The reaction solvent was anhydrous DCM (0.10 M) and anhydrous DMF (0.15 M) and the mixture was stirred overnight at room temperature under an argon atmosphere. The crude material was purified by flash chromatography (DCM/MeOH) to yield pure **SZ13TA(Me)** (61%). R_f = 0.28 in 4% MeOH/DCM.

^{1}H NMR (400 MHz, $(CD_3)_2CO$) δ 8.07 (d, *J* = 8.4 Hz, 2H), 7.91 (dd, *J* = 8.0, 1.6 Hz, 1H), 7.76 (d, *J* = 8.3 Hz, 2H), 7.71 – 7.64 (m, 1H), 7.46 (d, *J* = 9.6 Hz, 1H), 7.19 (t, *J* = 7.9 Hz, 1H), 5.49 (s, 2H), 2.02 (s, 3H).

HRMS-ESI (*m/z*): $[M+H]^+$ calculated for $C_{15}H_{15}N_2O_6S$, 351.0652 Da; found 351.0651 Da.

Synthesis of N-((4-((4-phenylpiperazin-1-yl)methyl)phenyl)sulfonyl)acetamide (SZ1TA(Me))

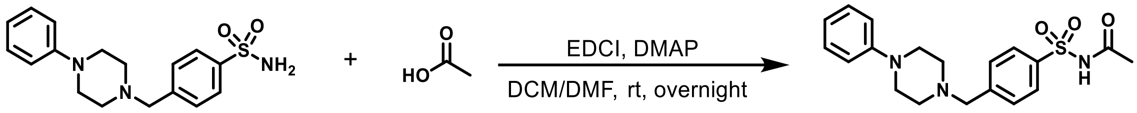

SA1

SZ1TA(Me)

The synthesis was carried out according to general procedure **(A)** using acetic acid and sulfonamide **SA1**. The reaction solvent was anhydrous DCM (0.10 M) and anhydrous DMF (0.15 M) and the mixture was stirred overnight at room temperature under an argon atmosphere. The crude material was purified by flash chromatography (DCM/MeOH) to yield pure **SZ13TA(Me)** (27%). R_f = 0.18 in 5% MeOH/DCM.

^{1}H NMR (400 MHz, $CDCl_3$) δ 8.01 (d, *J* = 8.4 Hz, 2H), 7.57 (d, *J* = 8.4 Hz, 2H), 7.32 – 7.21 (m, 2H), 6.93 (d, *J* = 8.1 Hz, 2H), 6.87 (t, *J* = 7.3 Hz, 1H), 3.65 (s, 2H), 3.21 (t, *J* = 5.0 Hz, 4H), 2.63 (t, *J* = 5.0 Hz, 4H), 2.08 (s, 3H).

HRMS-ESI (*m/z*): $[M+H]^+$ calculated for $C_{19}H_{24}N_3O_3S$, 374.1539 Da; found 374.1538 Da.

^{1}H NMR and HPLC spectra of *N*-acylsulfonamides

PAWS-1, SZ36TA1

^{1}H NMR (400 MHz) in CD_3OD

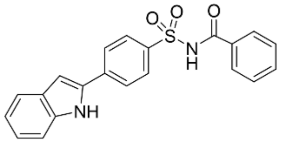

SZ36TA1

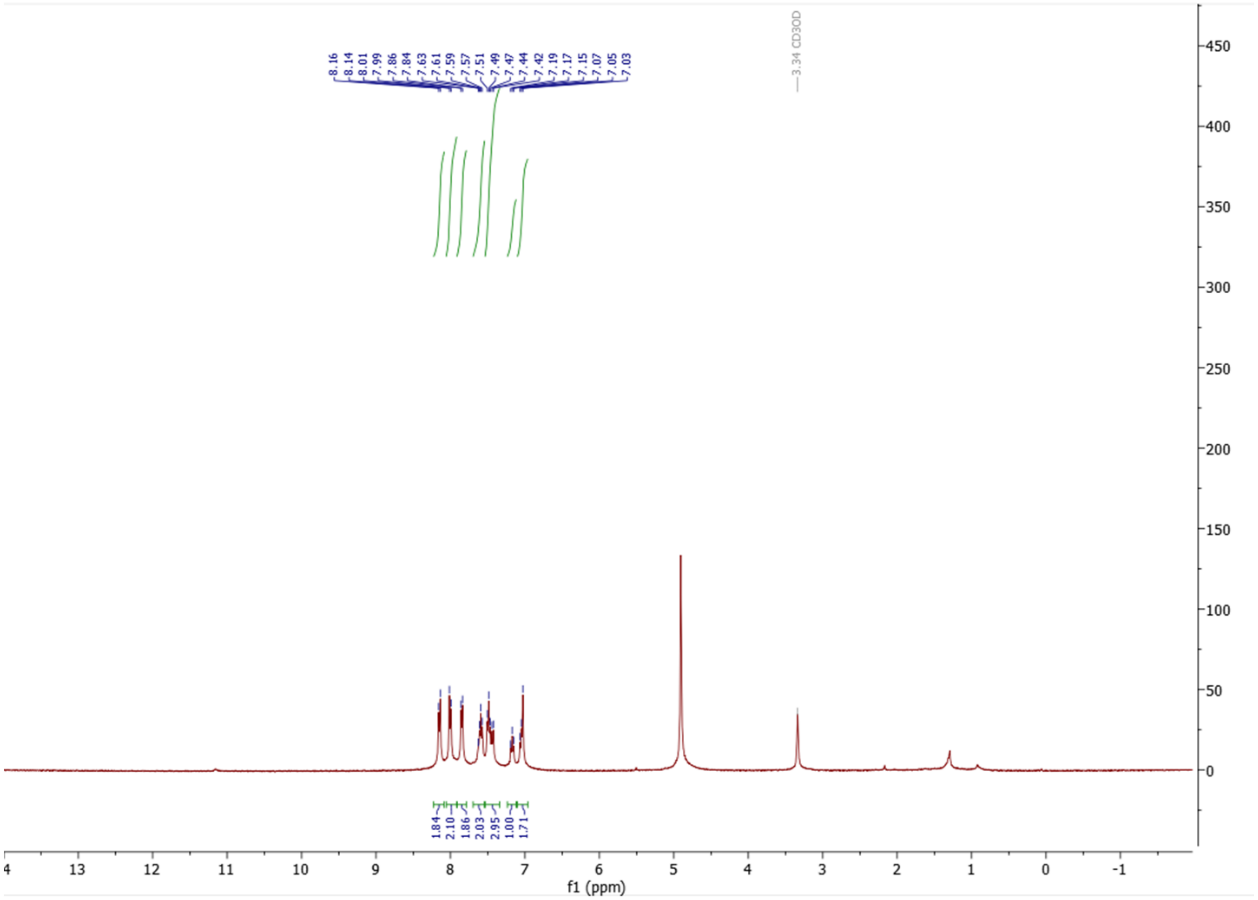

PAWS-2, SZ22TA45

^{1}H NMR (400 MHz) in $CDCl_3$

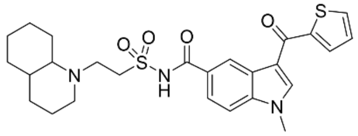

SZ22TA45

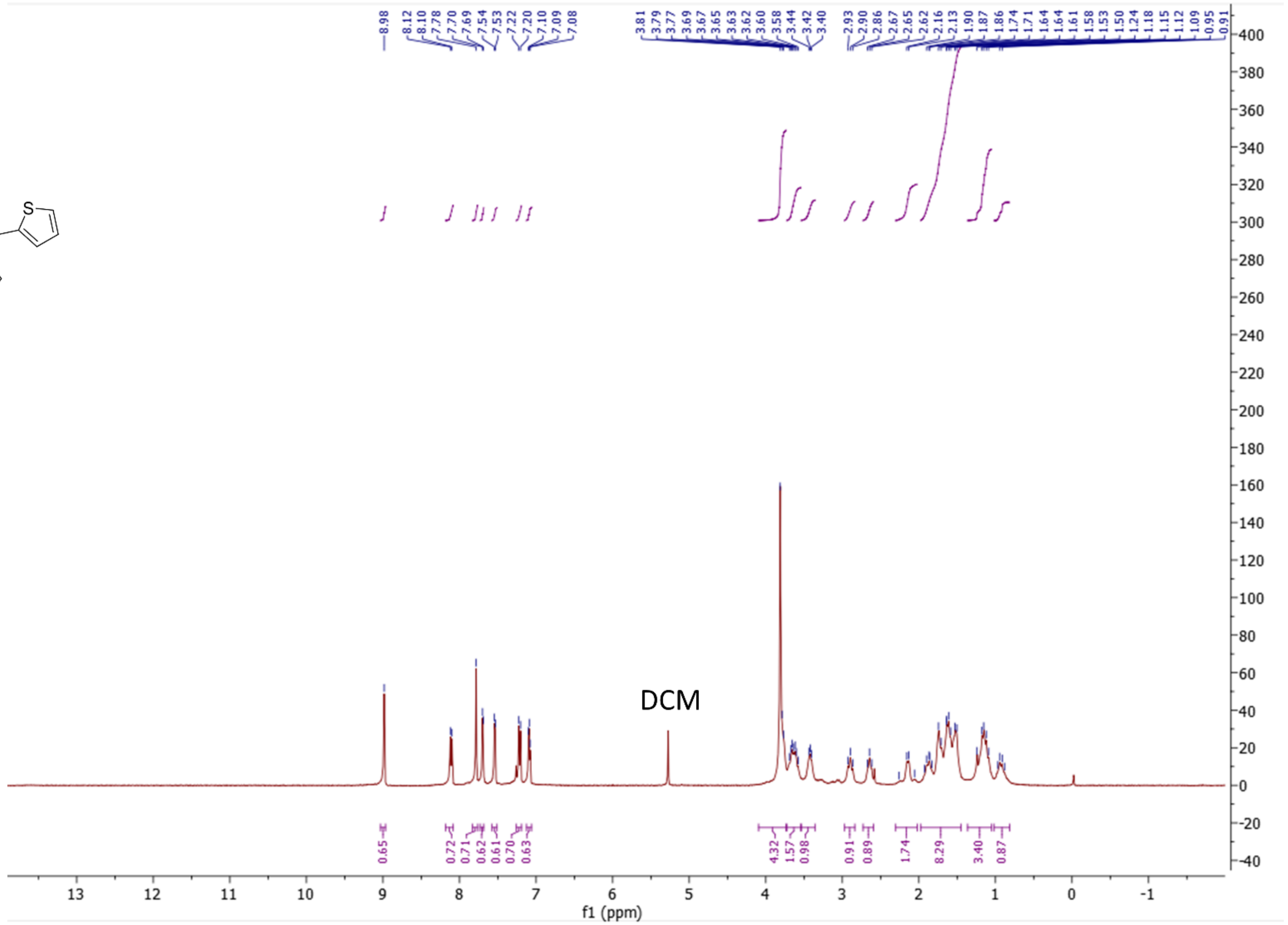

PAWS-3, SZ33TA45

^{1}H NMR (400 MHz) in $(CD_3)_2SO$

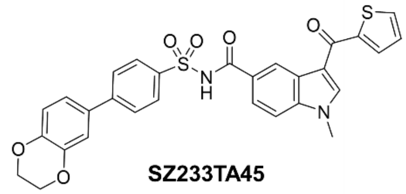

SZ233TA45

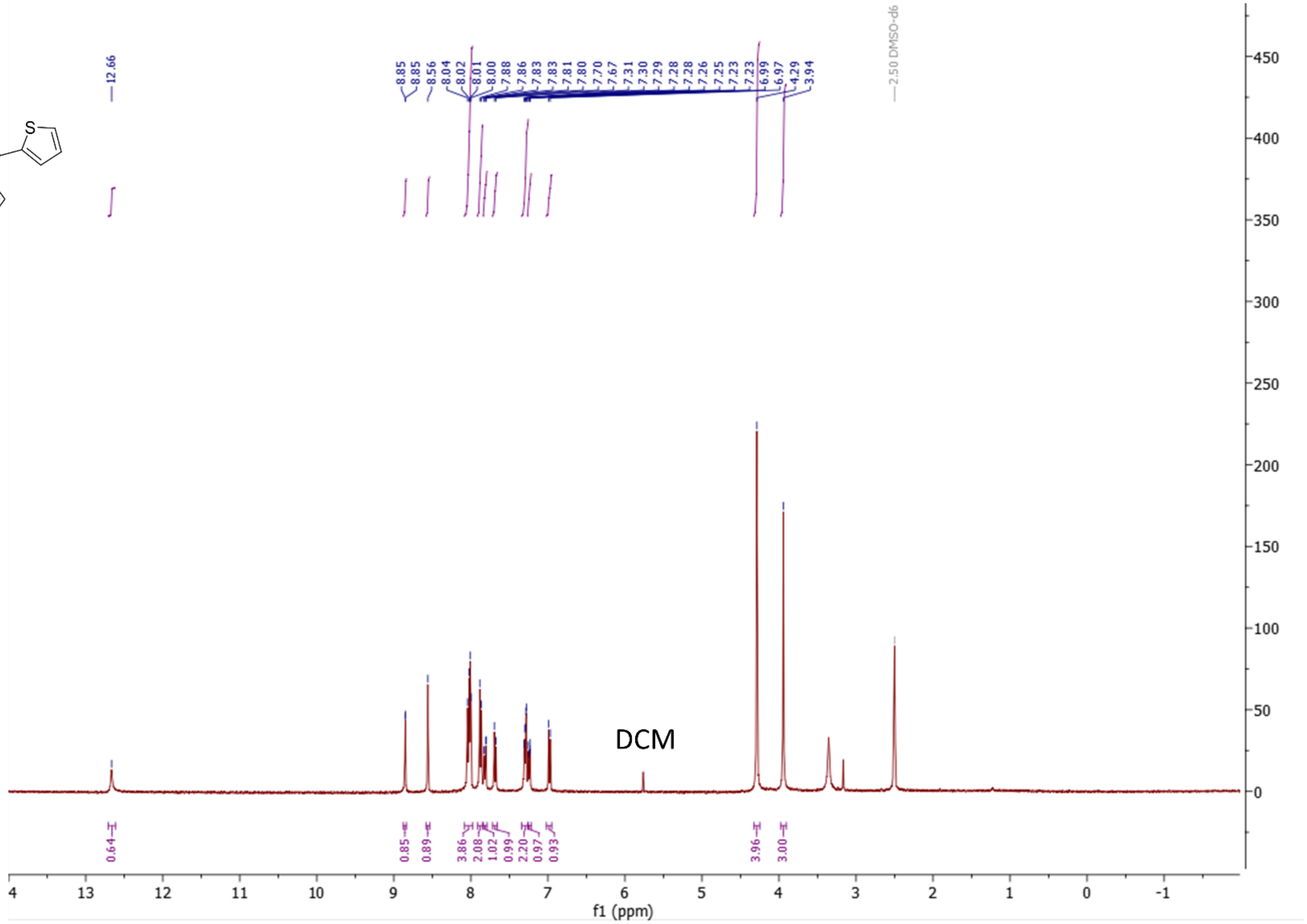

PAWS-4, SZ23TA5

^{1}H NMR (500 MHz) in $(CD_3)_2SO$

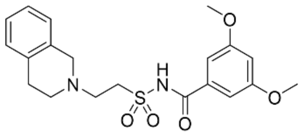

SZ23TA5

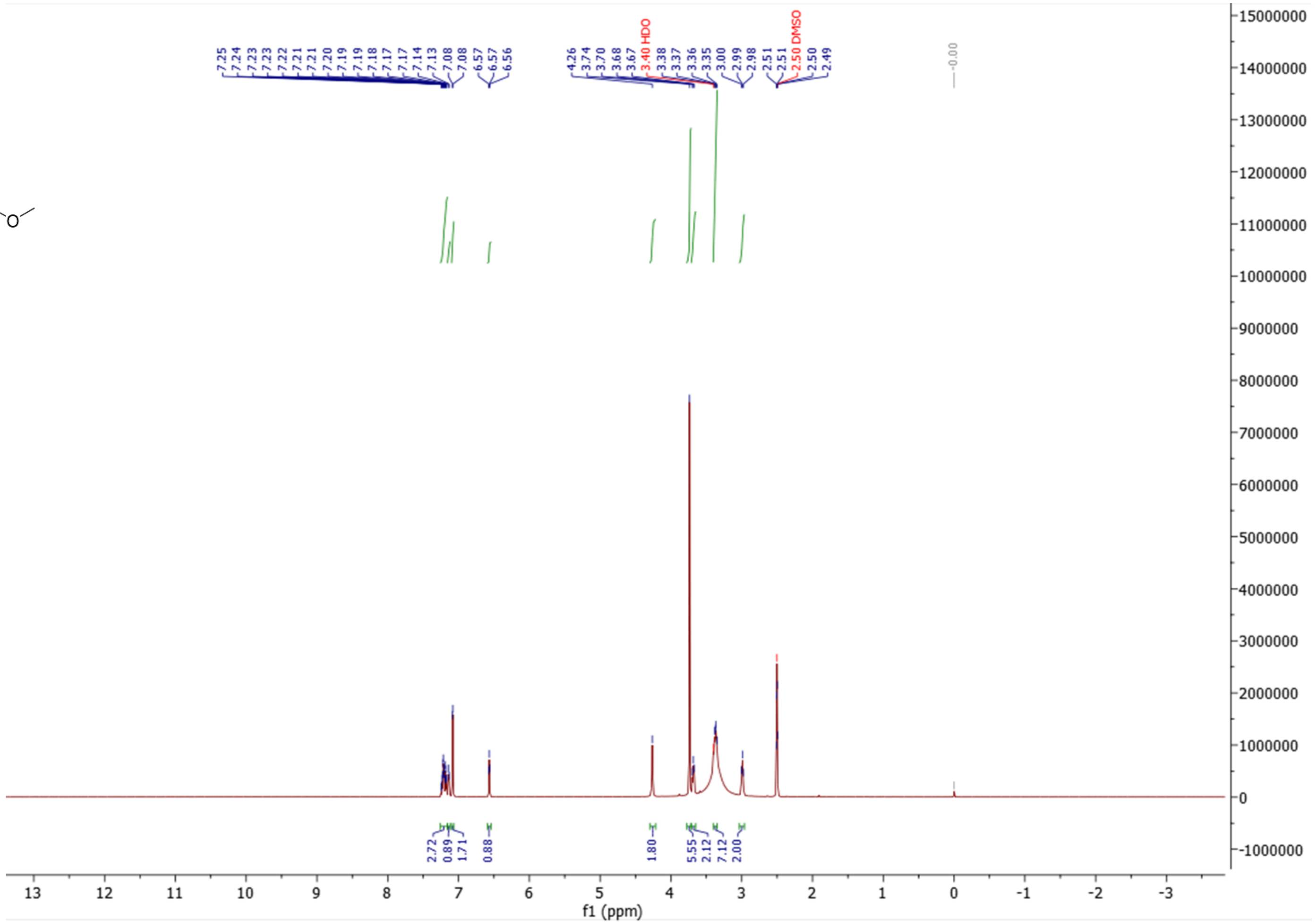

PAWS-5, SZ26TA45

^{1}H NMR (400 MHz) in $CDCl_3$

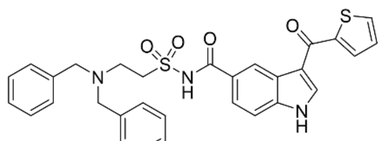

SZ26TA45

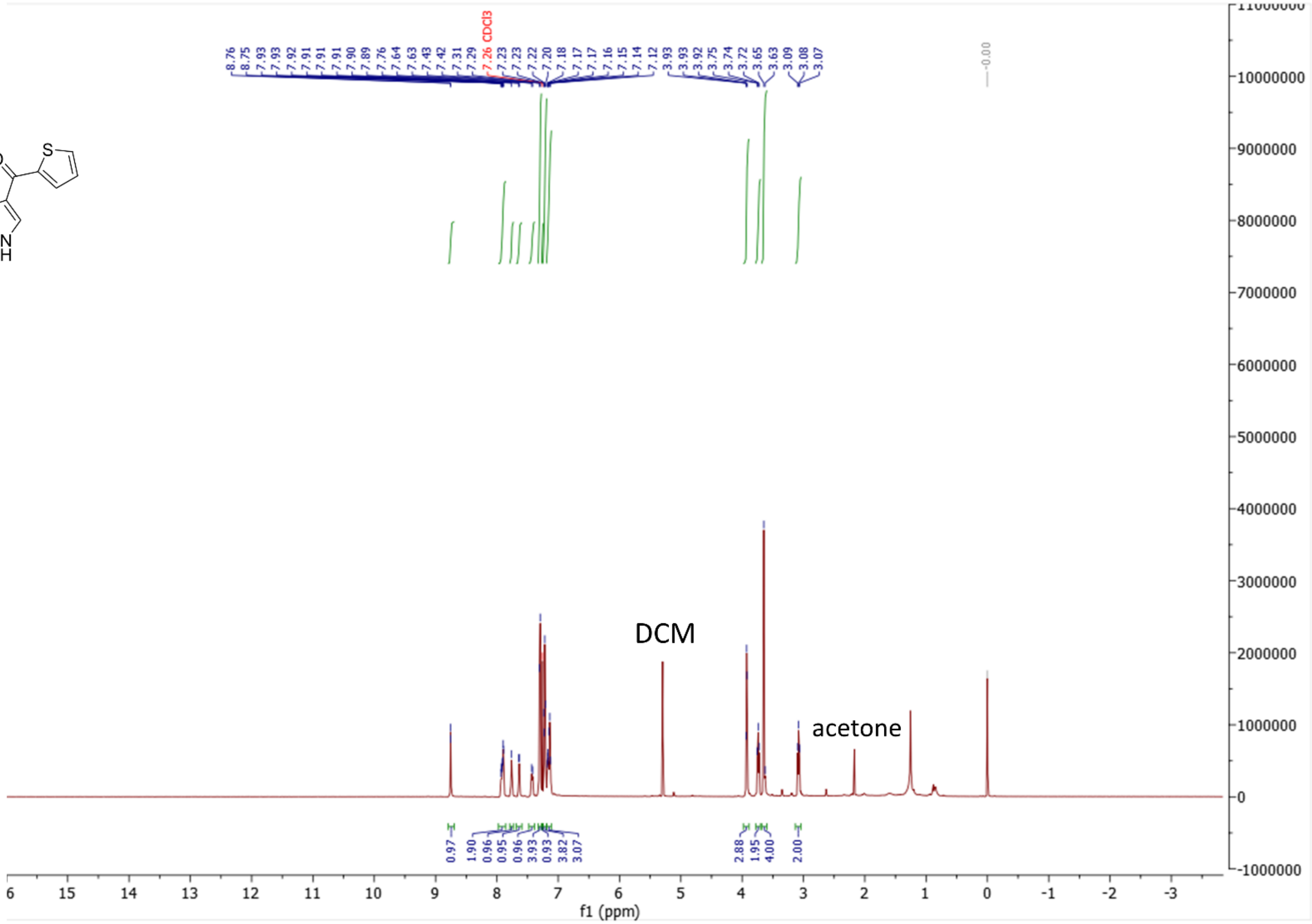

PAWS-6, SZ16TA19

^{1}H NMR (500 MHz) in $(CD_3)_2SO$

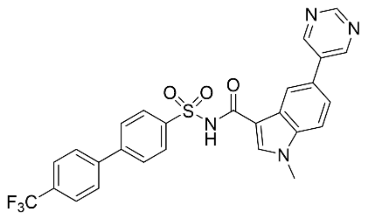

SZ16TA19

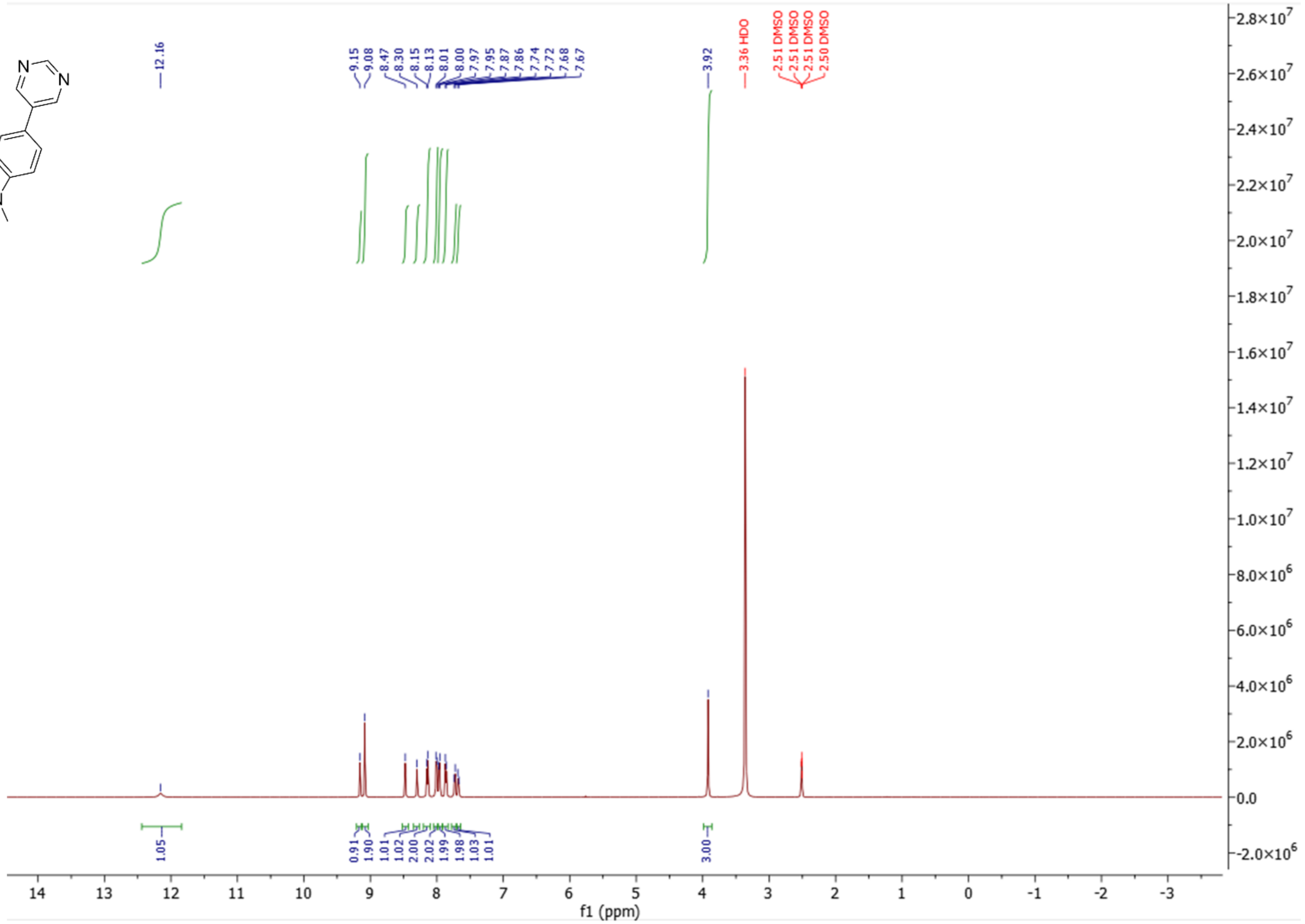

PAWS-7, SZ35TA30

^{1}H NMR (500 MHz) in $(CD_3)_2SO$

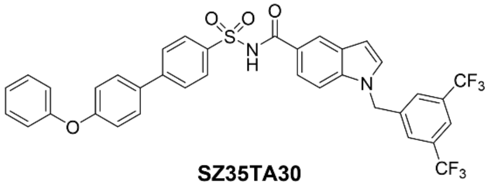

SZ35TA30

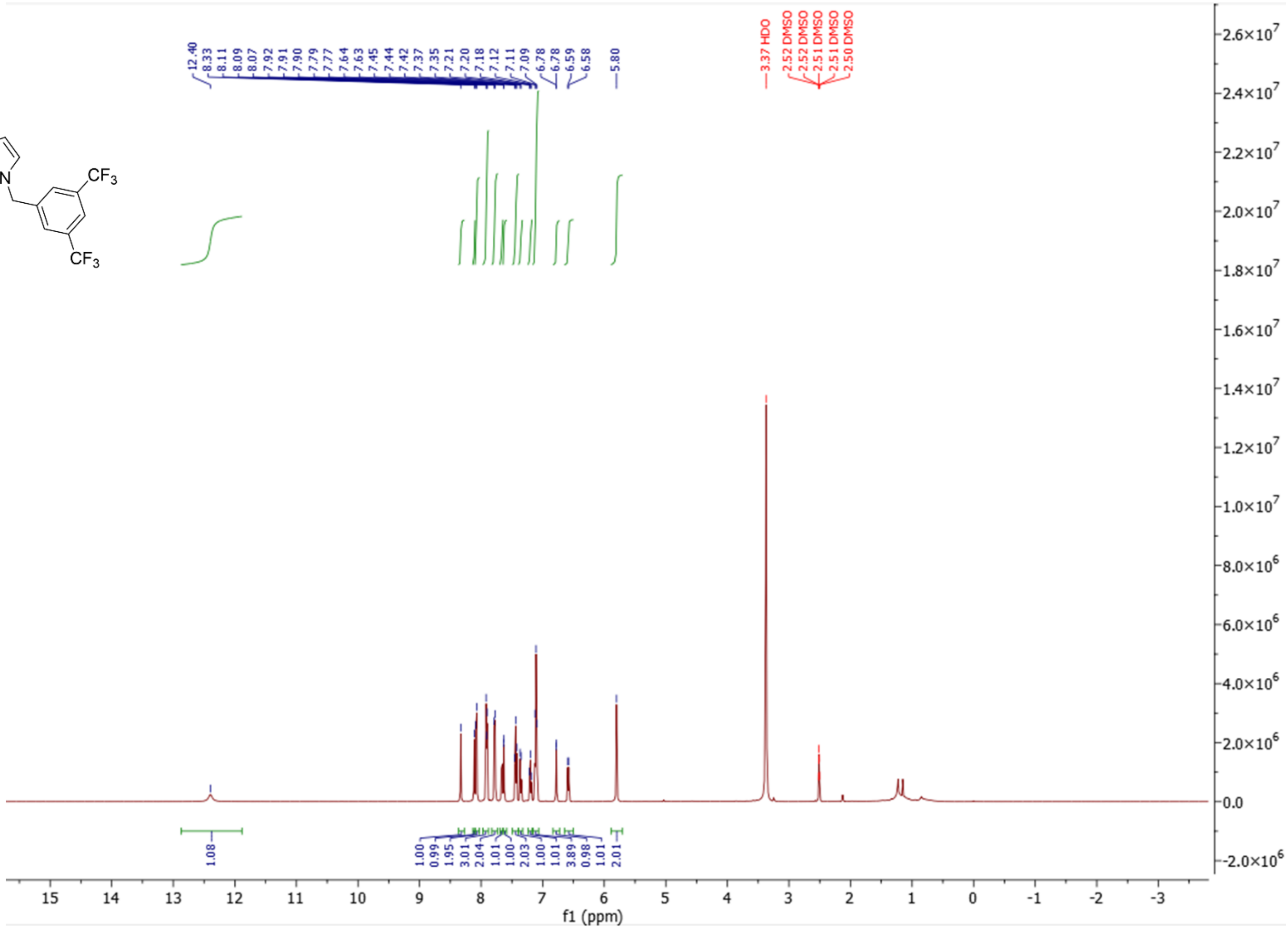

PAWS-8, SZ1TA5

^{1}H NMR (500 MHz) in $(CD_3)_2SO$

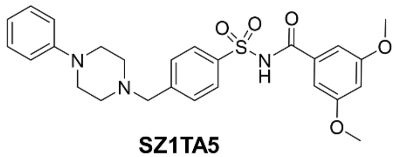
SZ1TA5

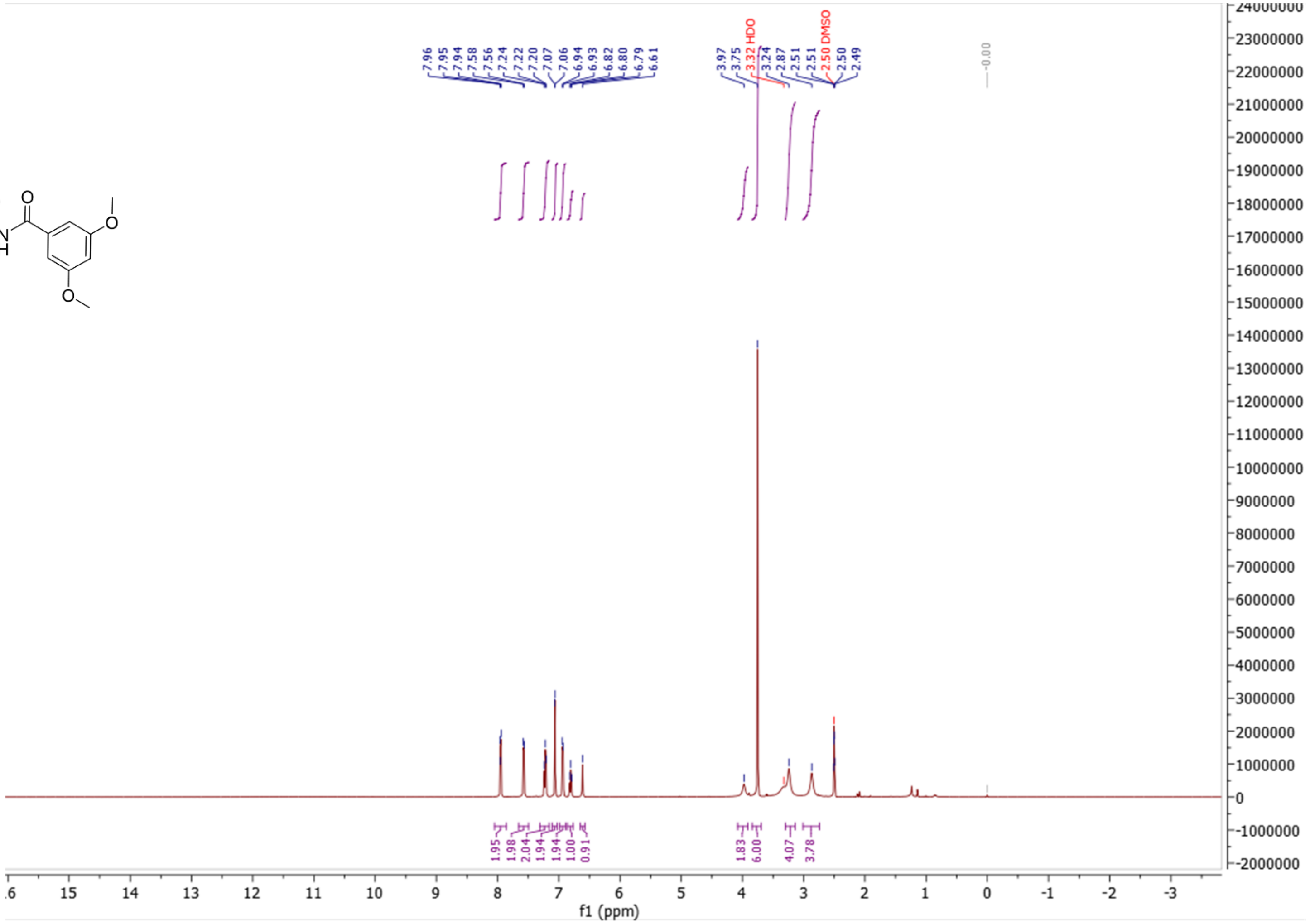

PAWS-9, SZ2TA3

^{1}H NMR (500 MHz) in $(CD_3)_2SO$

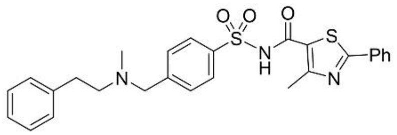

SZ2TA3

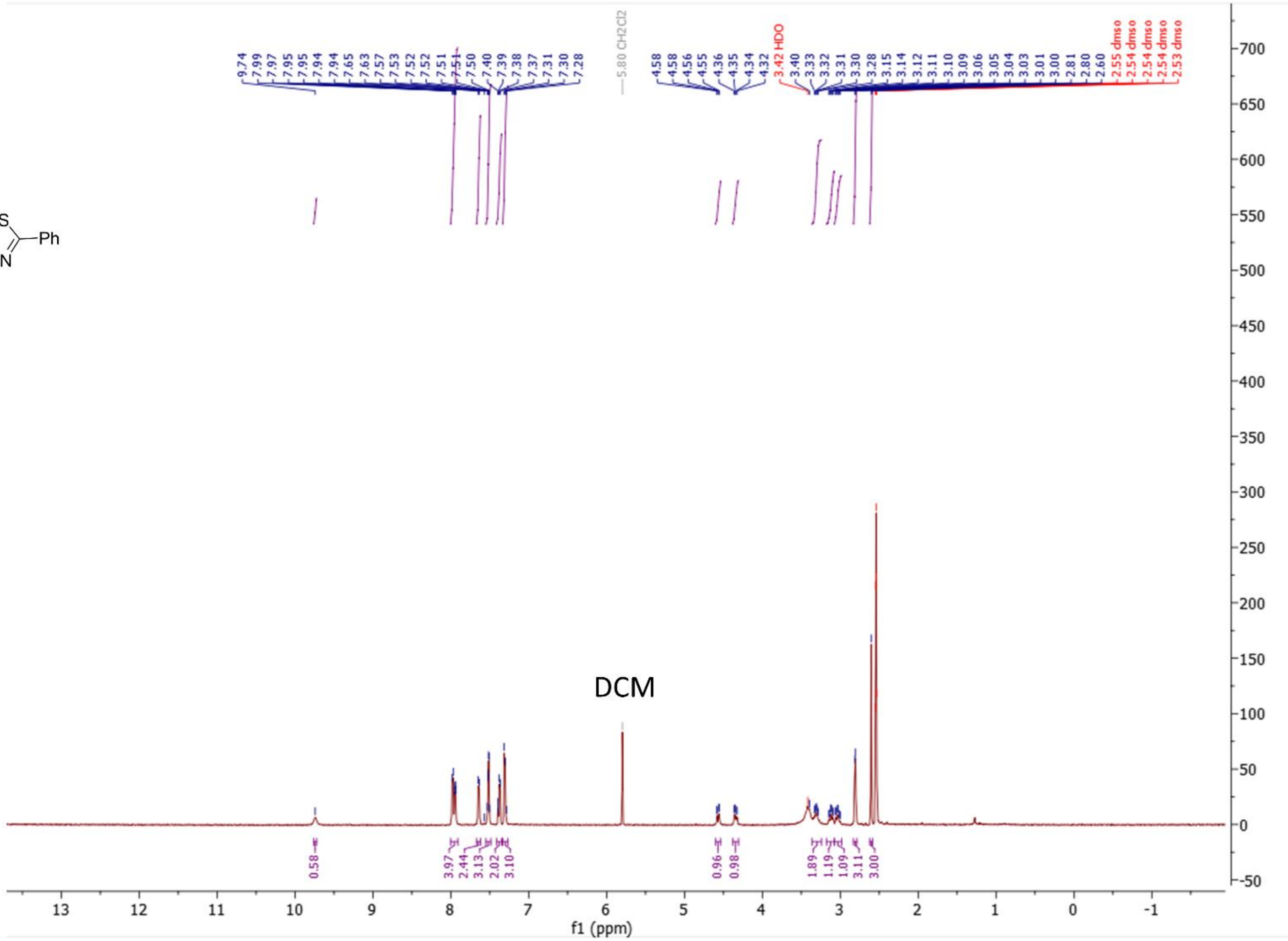

PAWS-10, SZ2TA5

^{1}H NMR (500 MHz) in $(CD_3)_2SO$

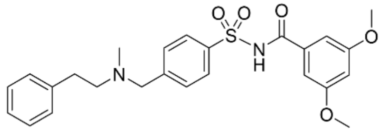

SZ2TA5

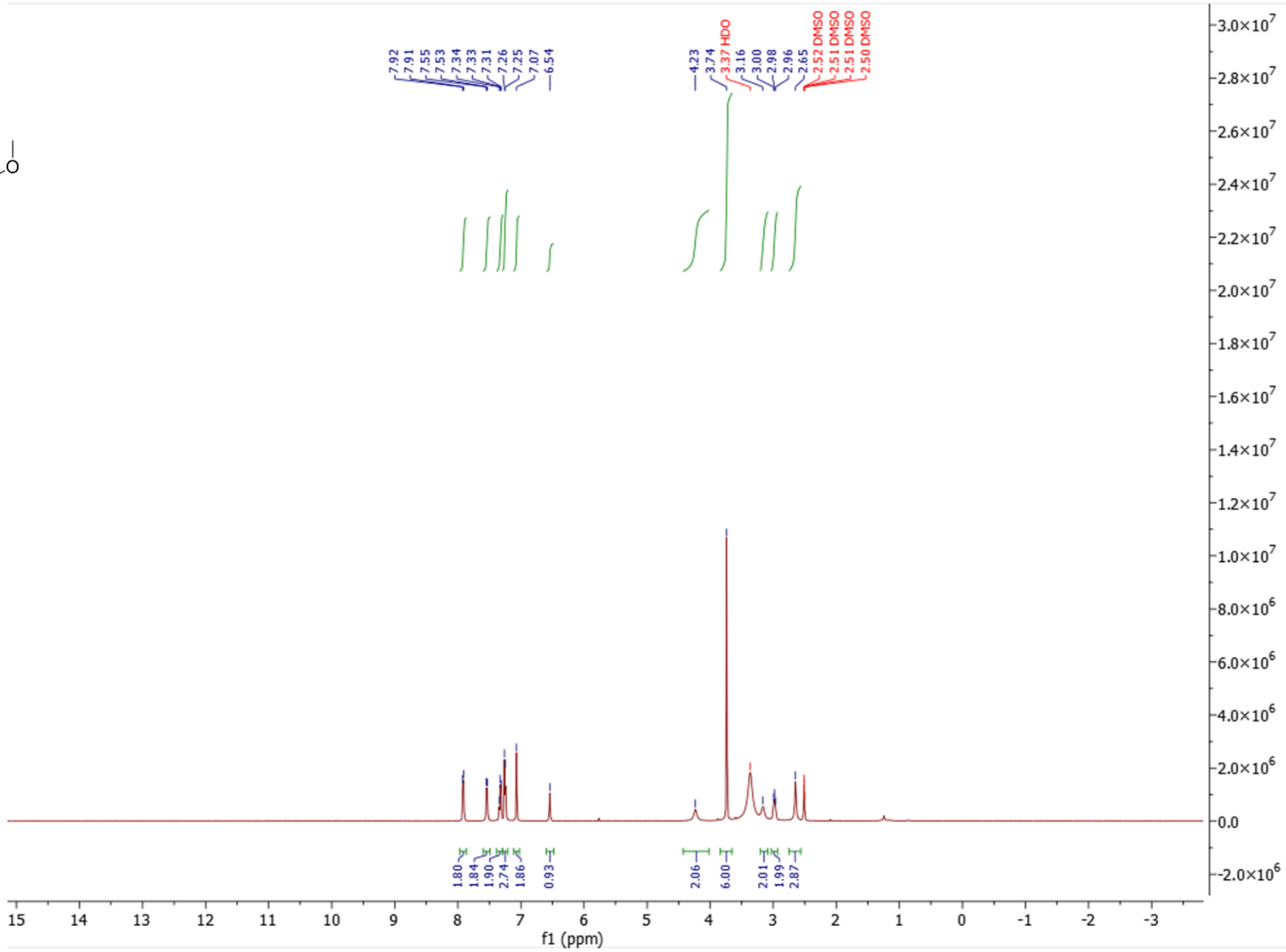

PAWS-11, SZ35TA5

^{1}H NMR (500 MHz) in $(CD_3)_2CO$

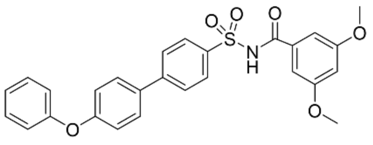

SZ35TA5

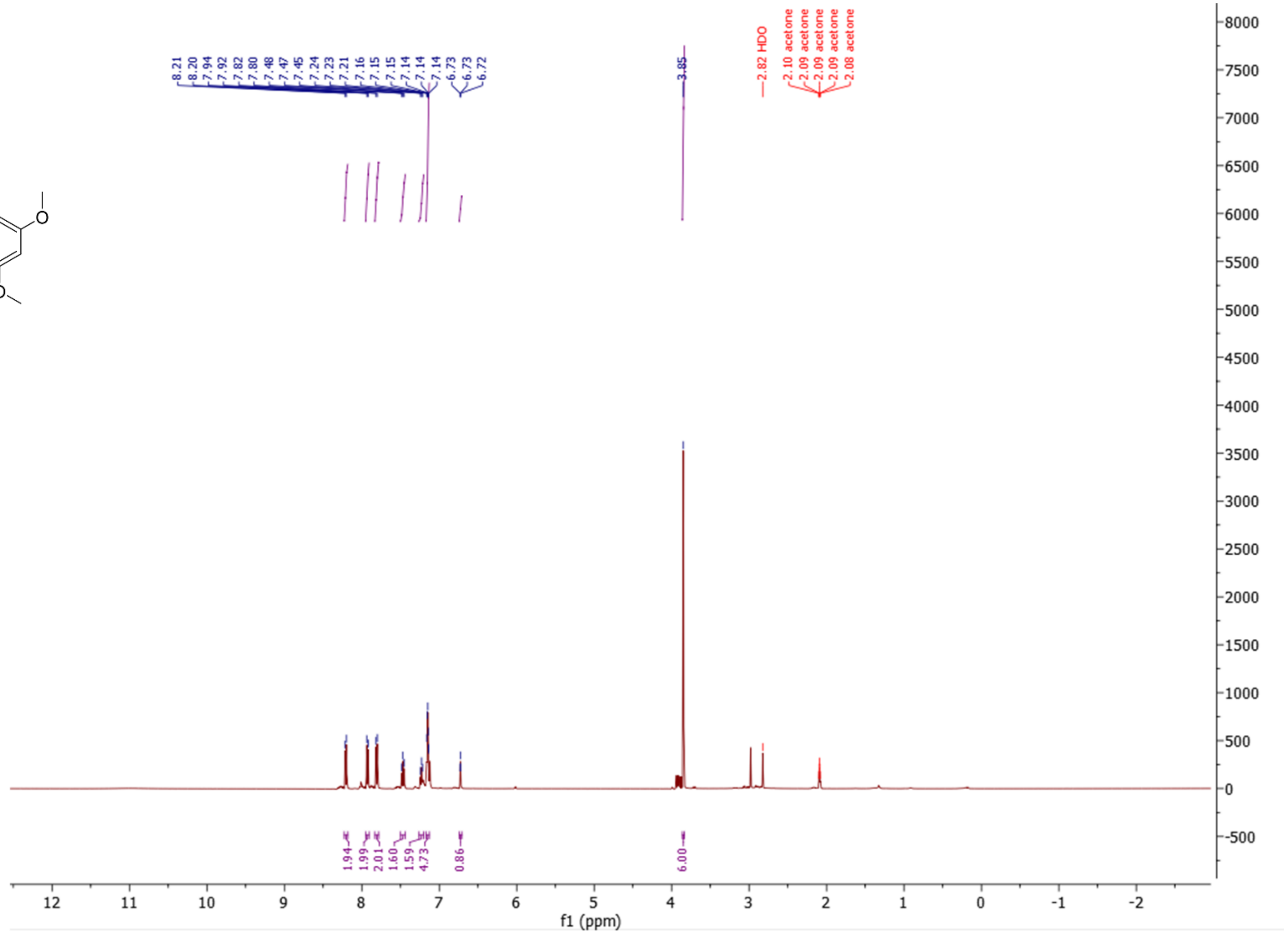

PAWS-12, SZ35TA23

^{1}H NMR (500 MHz) in $(CD_3)_2SO$

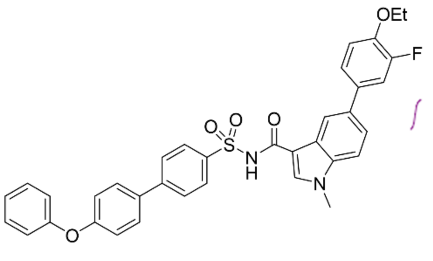

SZ35TA23

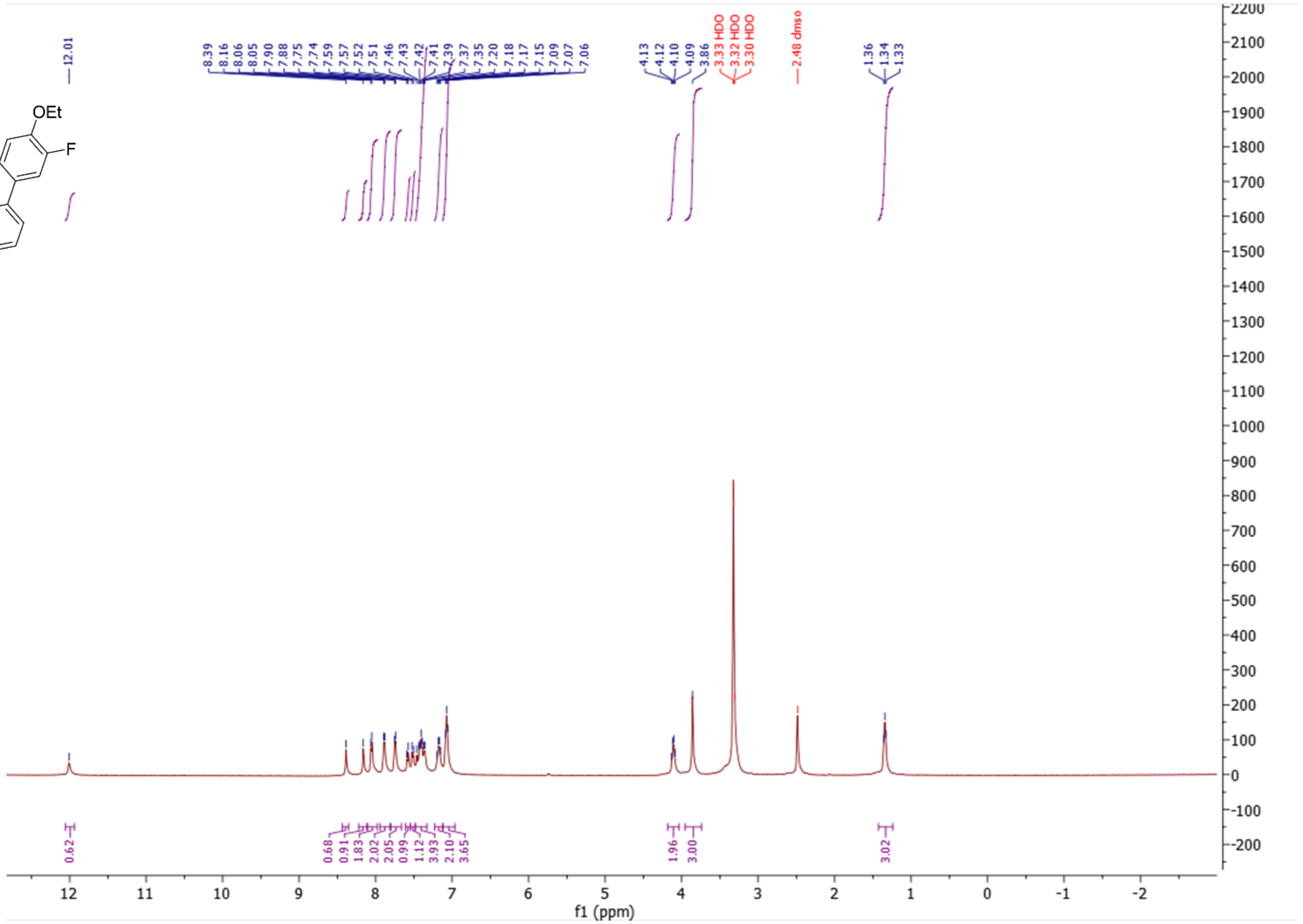

PAWS-13, SZ27TA2

^{1}H NMR (500 MHz) in $CDCl_3$

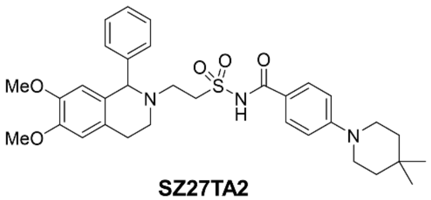

SZ27TA2

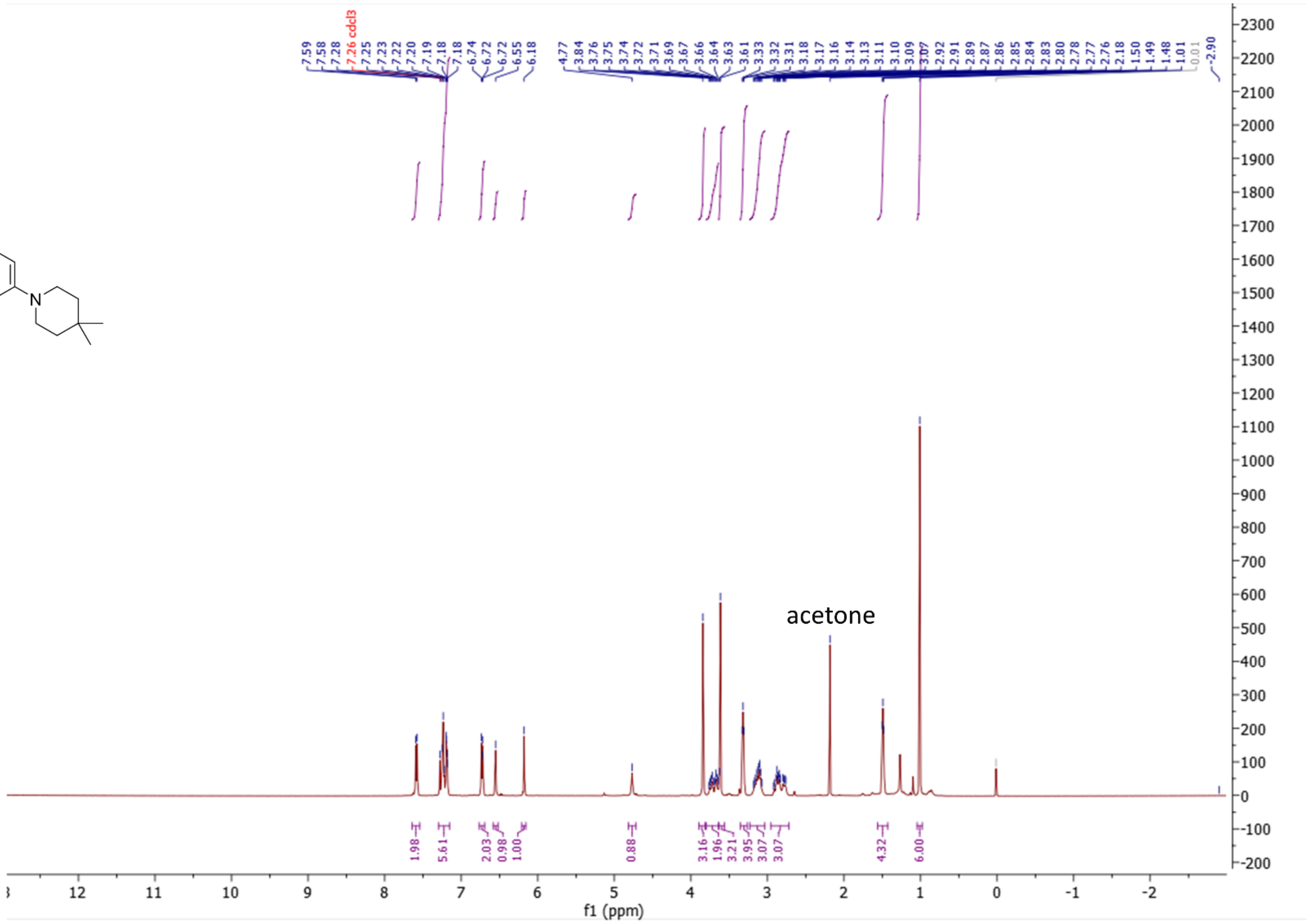

PAWS-14, SZ18TA23

^{1}H NMR (500 MHz) in $(CD_3)_2SO$

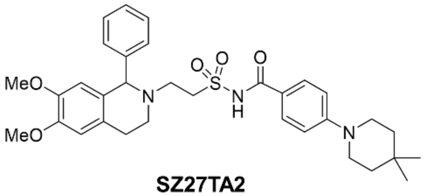

SZ27TA2

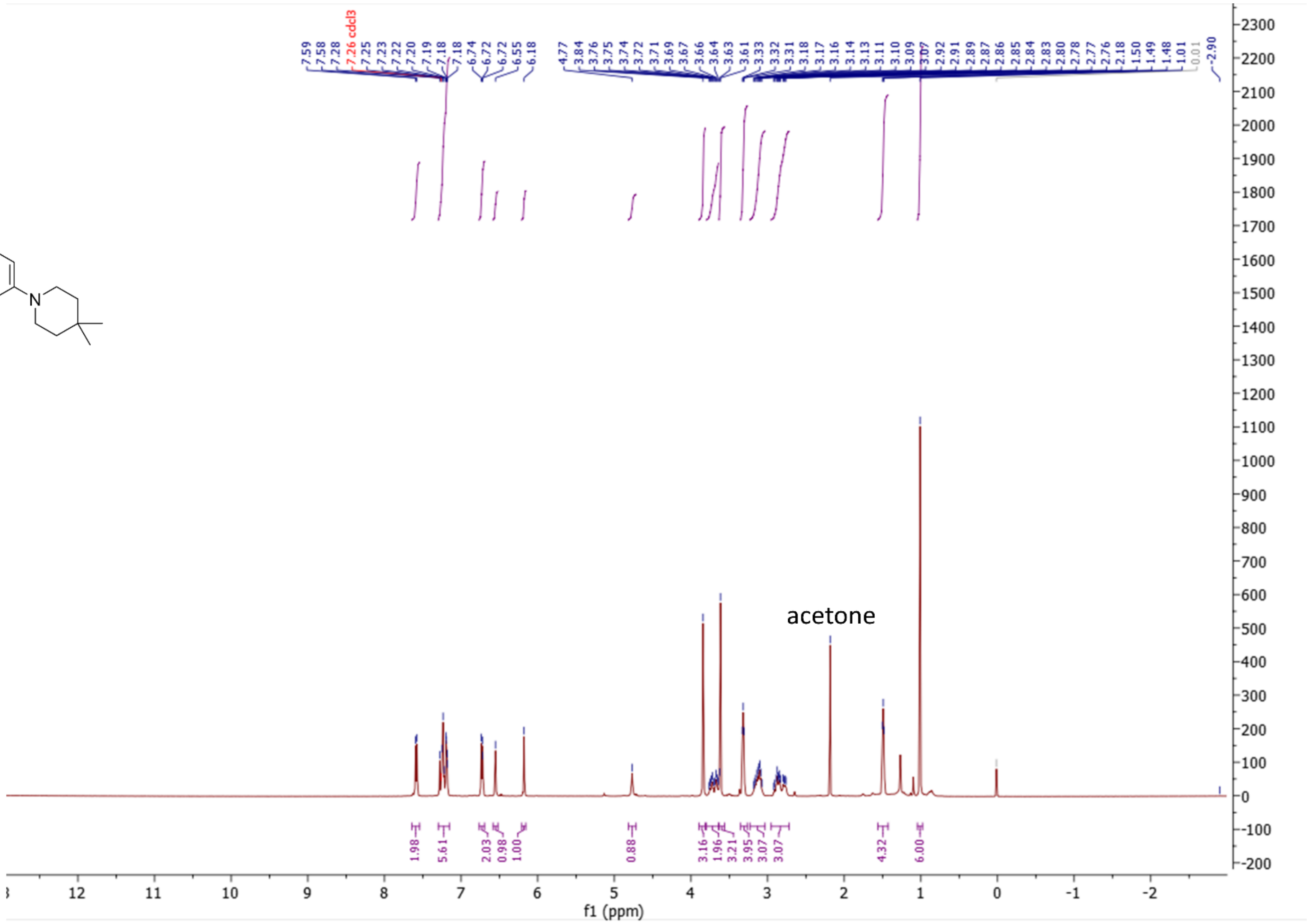

PAWS-16, SZ23TA14

^{1}H NMR (500 MHz) in $(CD_3)_2SO$

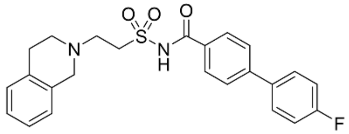

SZ23TA14

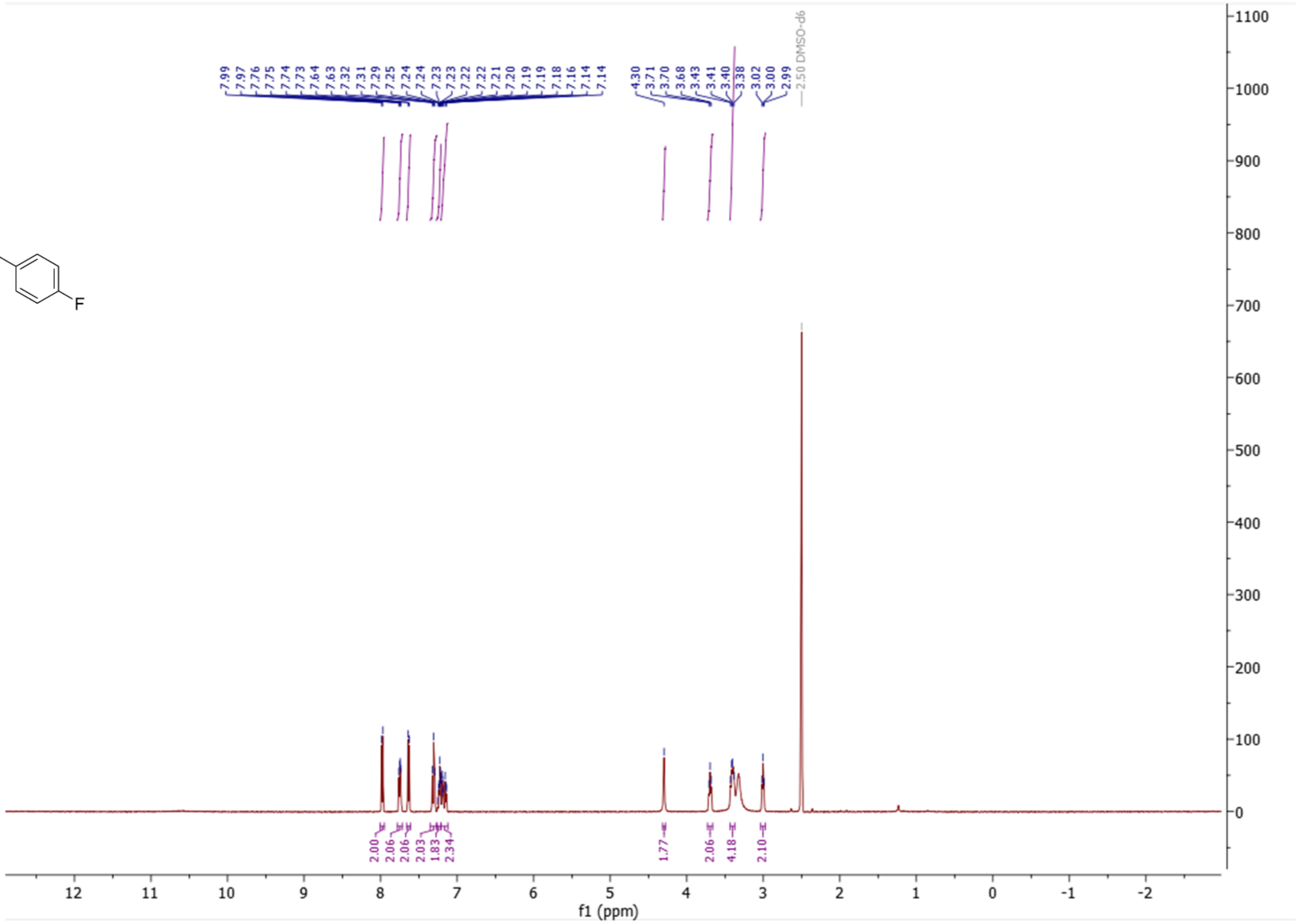

PAWS-17, SZ34TA5

^{1}H NMR (400 MHz) in $CDCl_3$

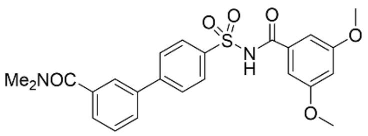

SZ34TA5

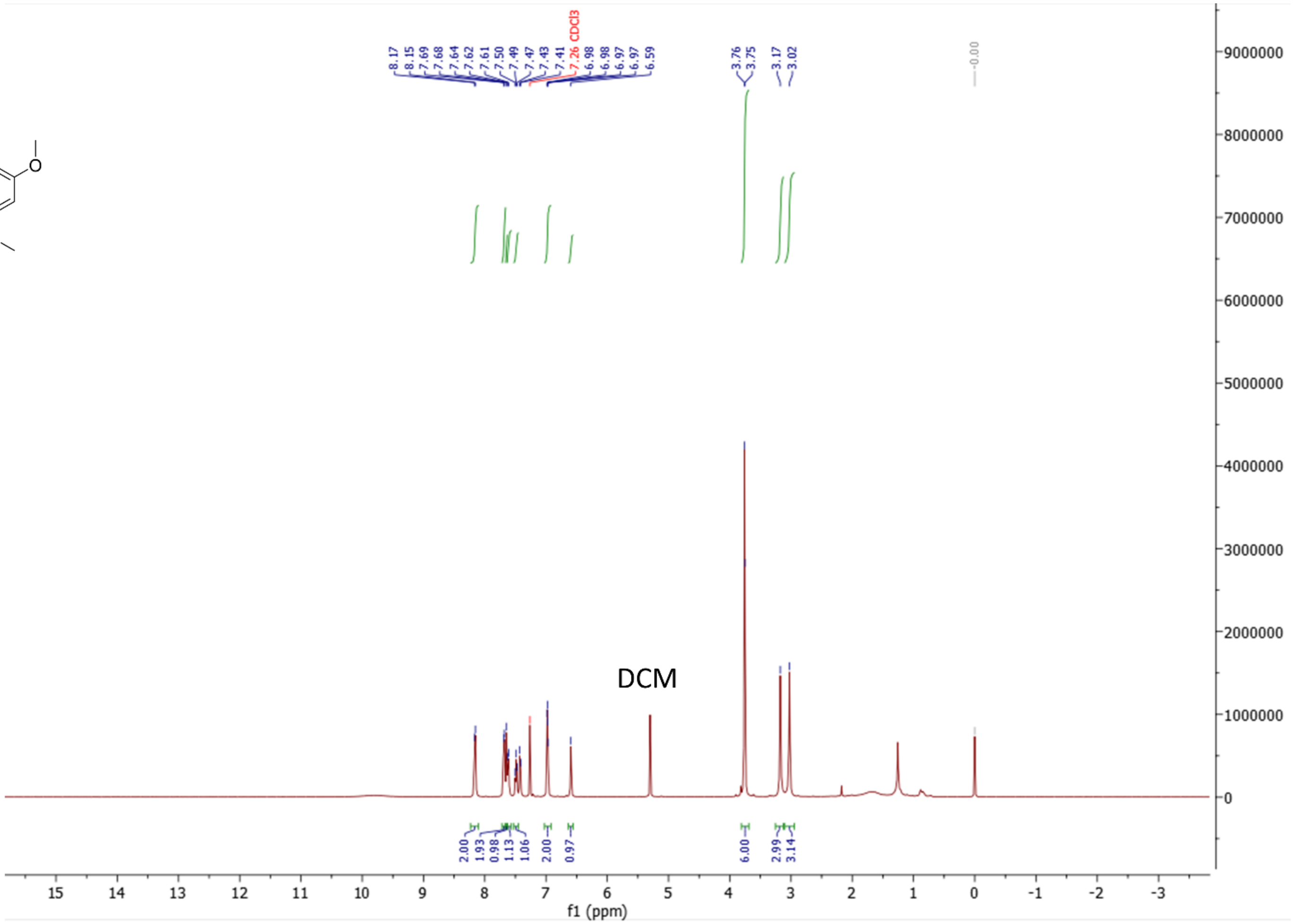

PAWS-18, SZ25TA44

^{1}H NMR (500 MHz) in $CDCl_3$

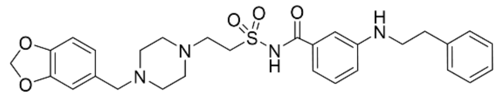

SZ25TA44

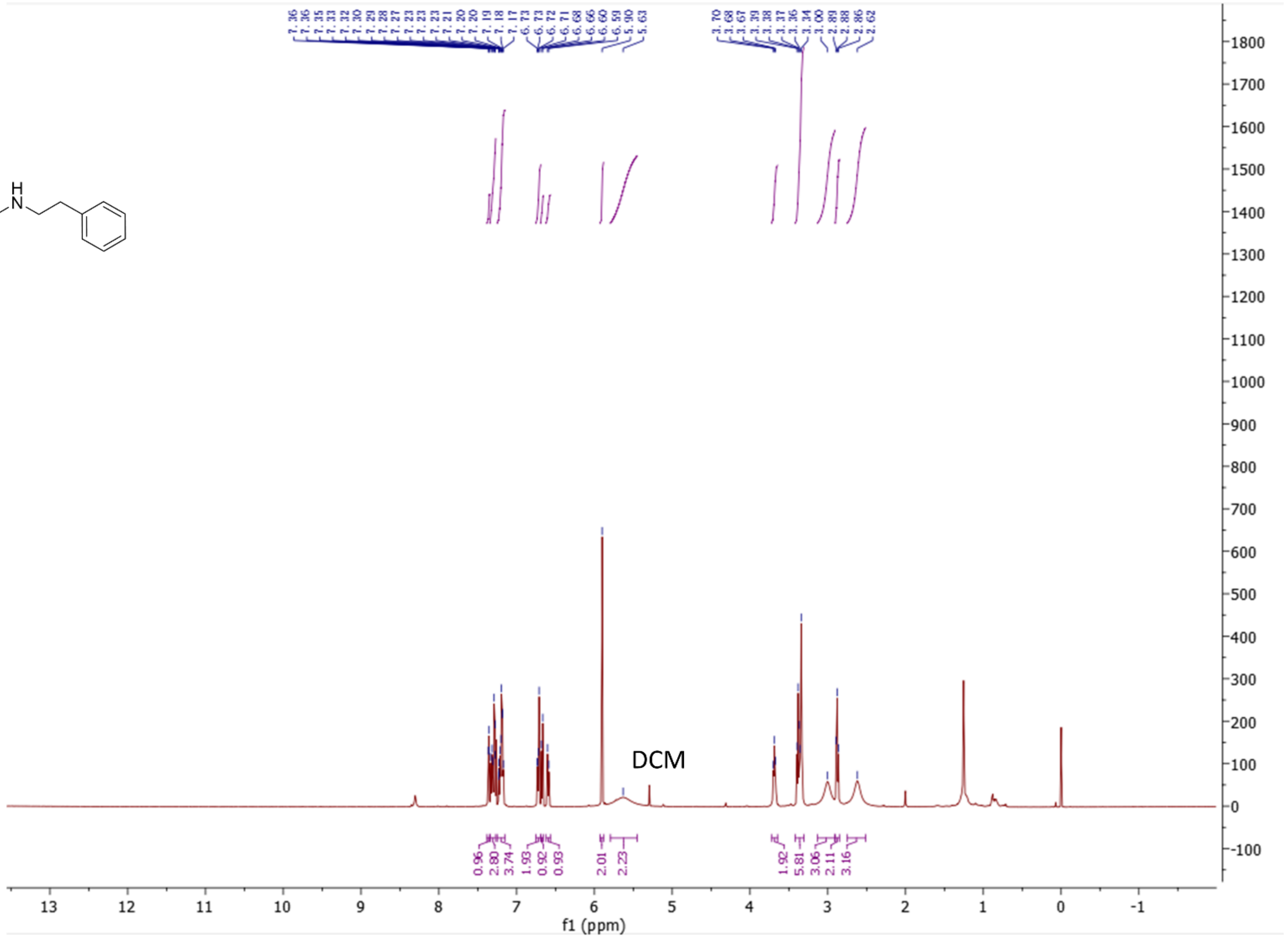

PAWS-19, SZ22TA14

^{1}H NMR (500 MHz) in $CDCl_3$

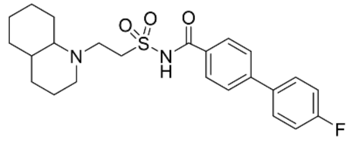

SZ22TA14

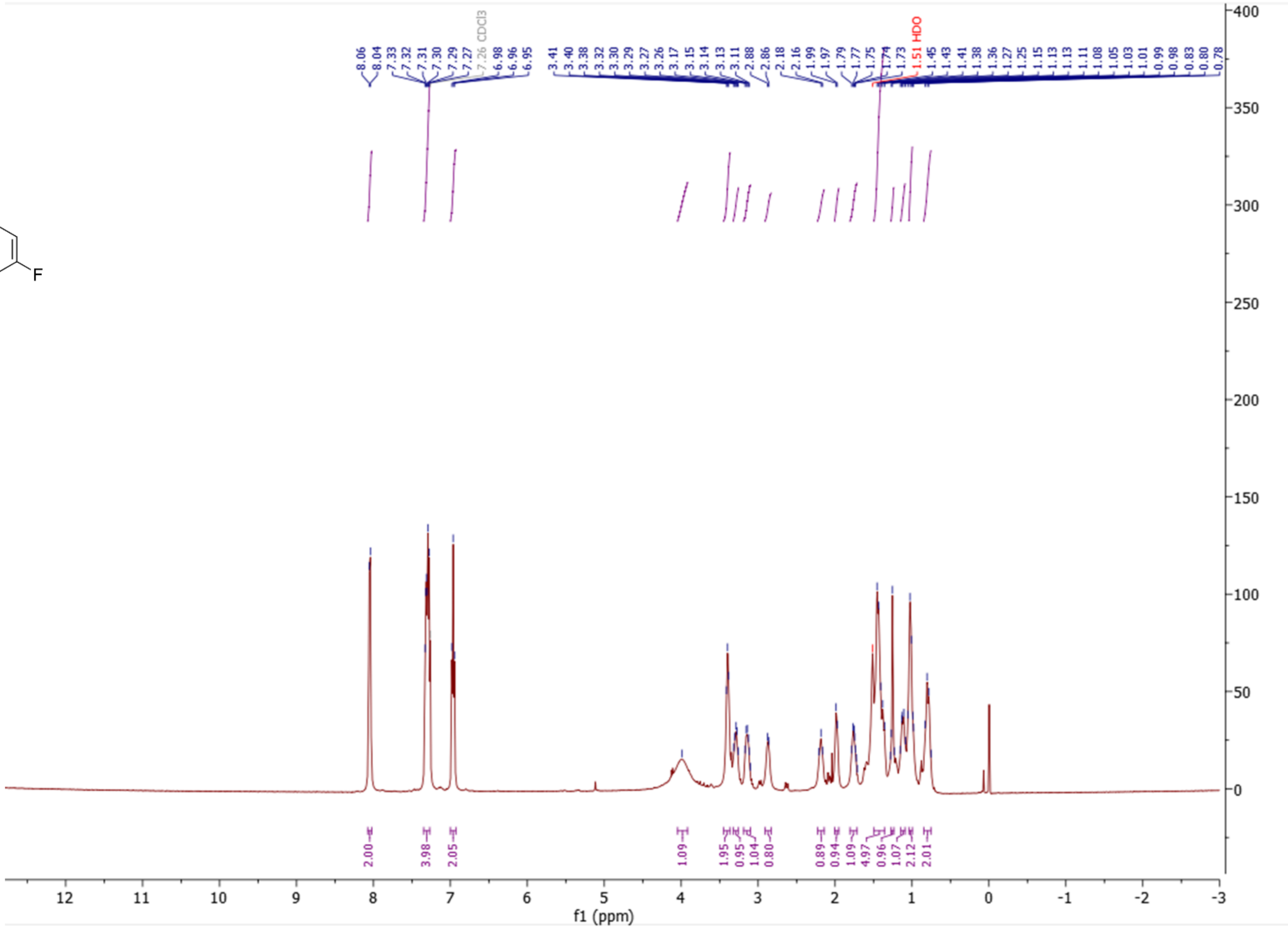

PAWS-20, SZ3TA44

^{1}H NMR (500 MHz) in CD_3OD

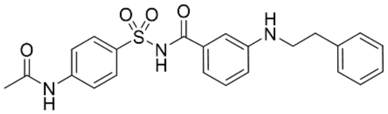

SZ3TA44

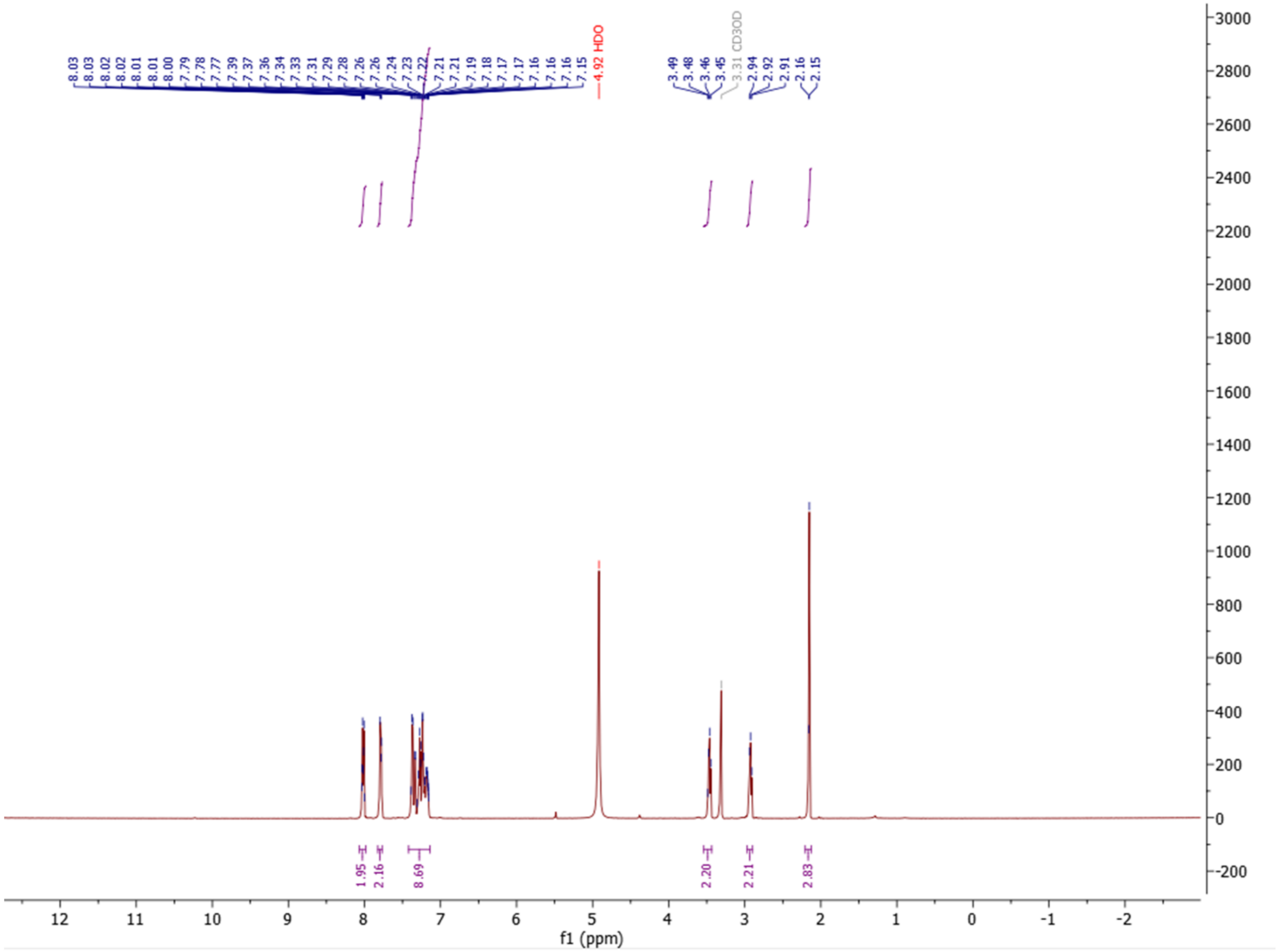

PAWS-21, SZ22TA44

^{1}H NMR (500 MHz) in CD_3OD

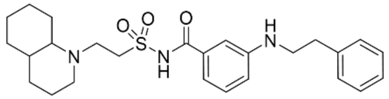

SZ22TA44

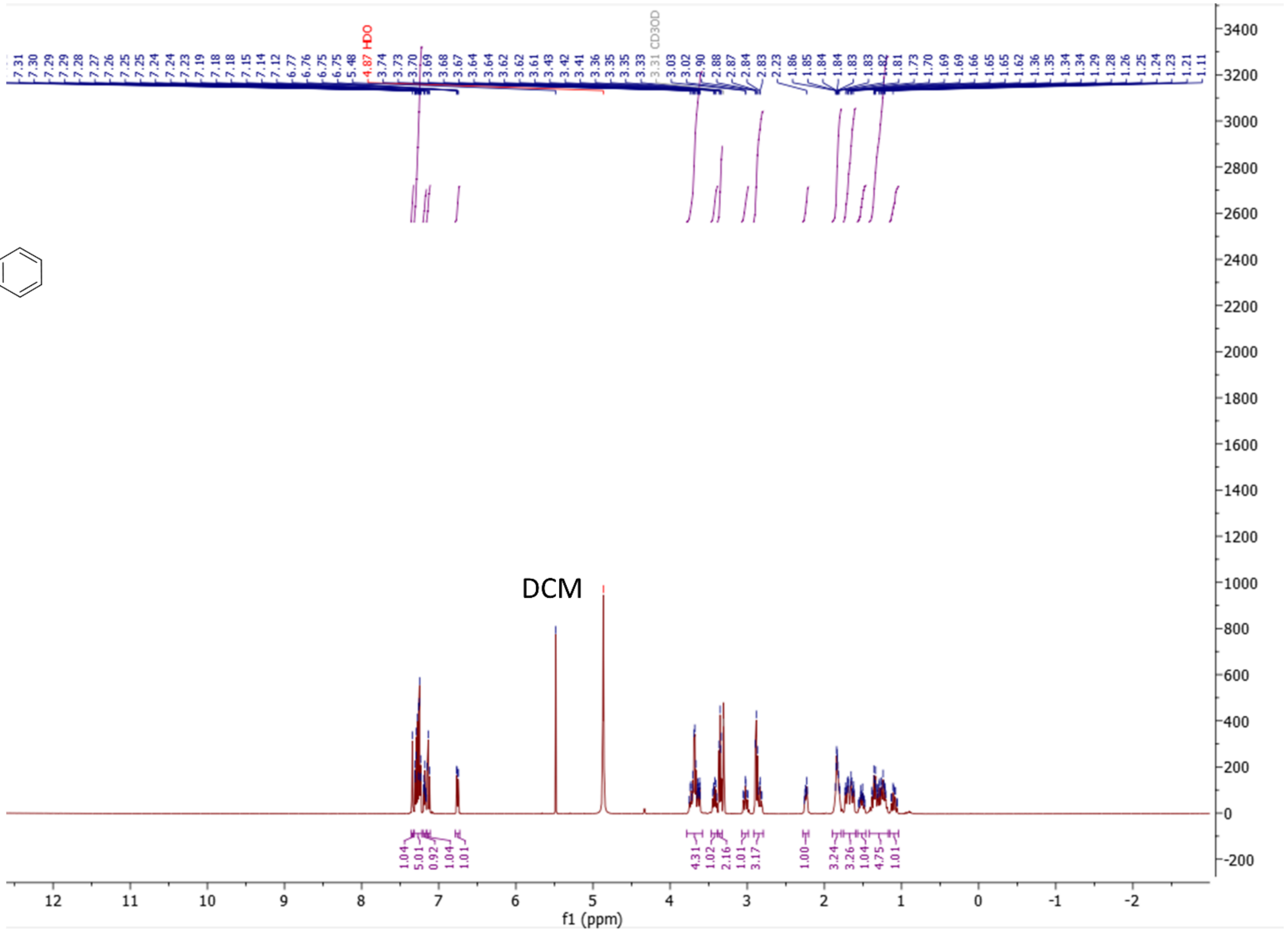

PAWS-22, SZ26TA44

^{1}H NMR (500 MHz) in CD_3OD

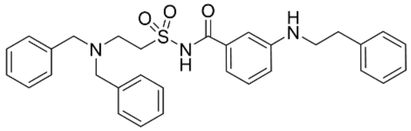

SZ26TA44

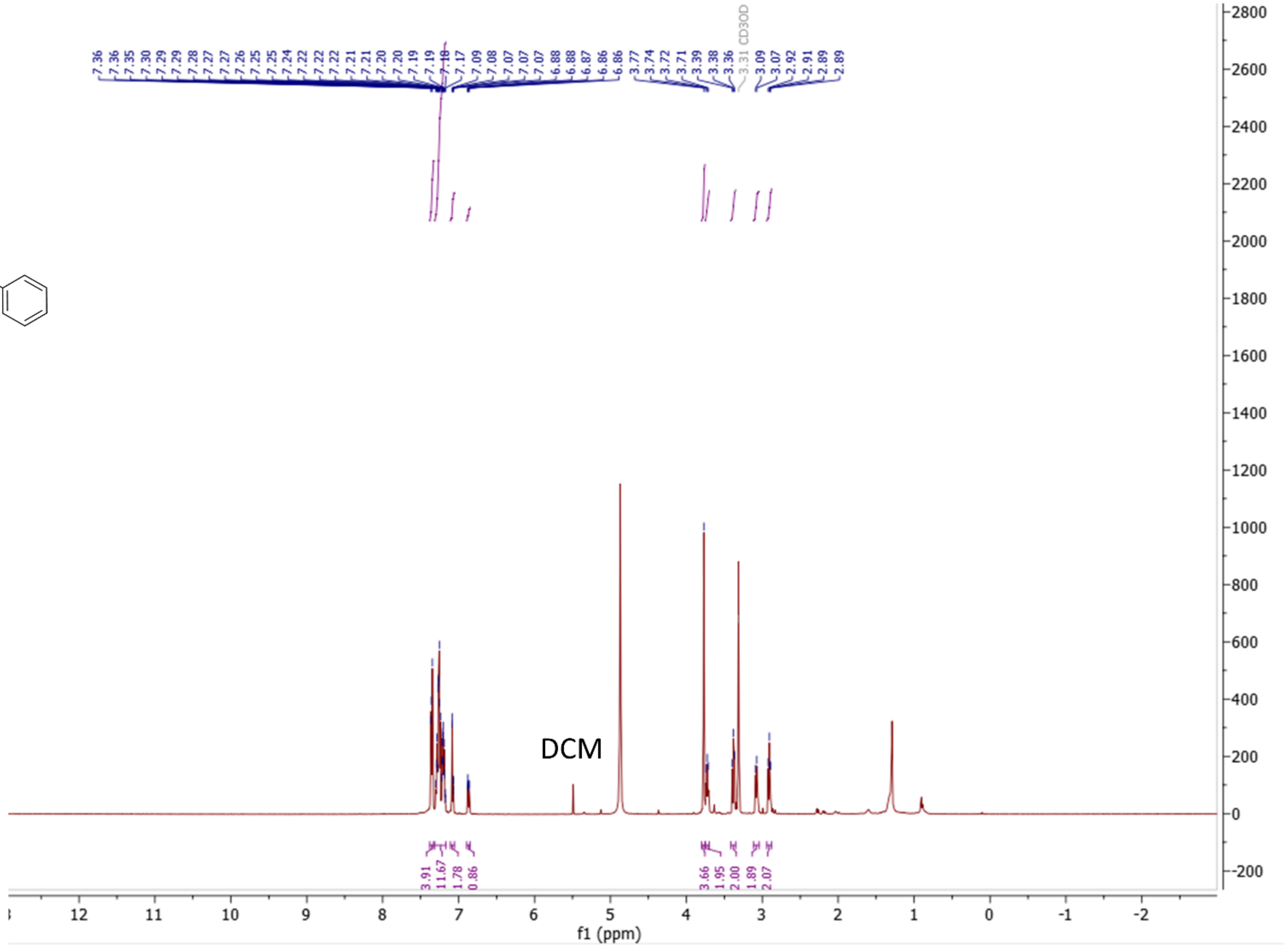

PAWS-23, SZ3TA4

^{1}H NMR (500 MHz) in $CDCl_3$

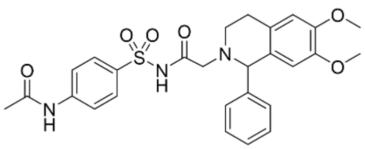

SZ3TA4

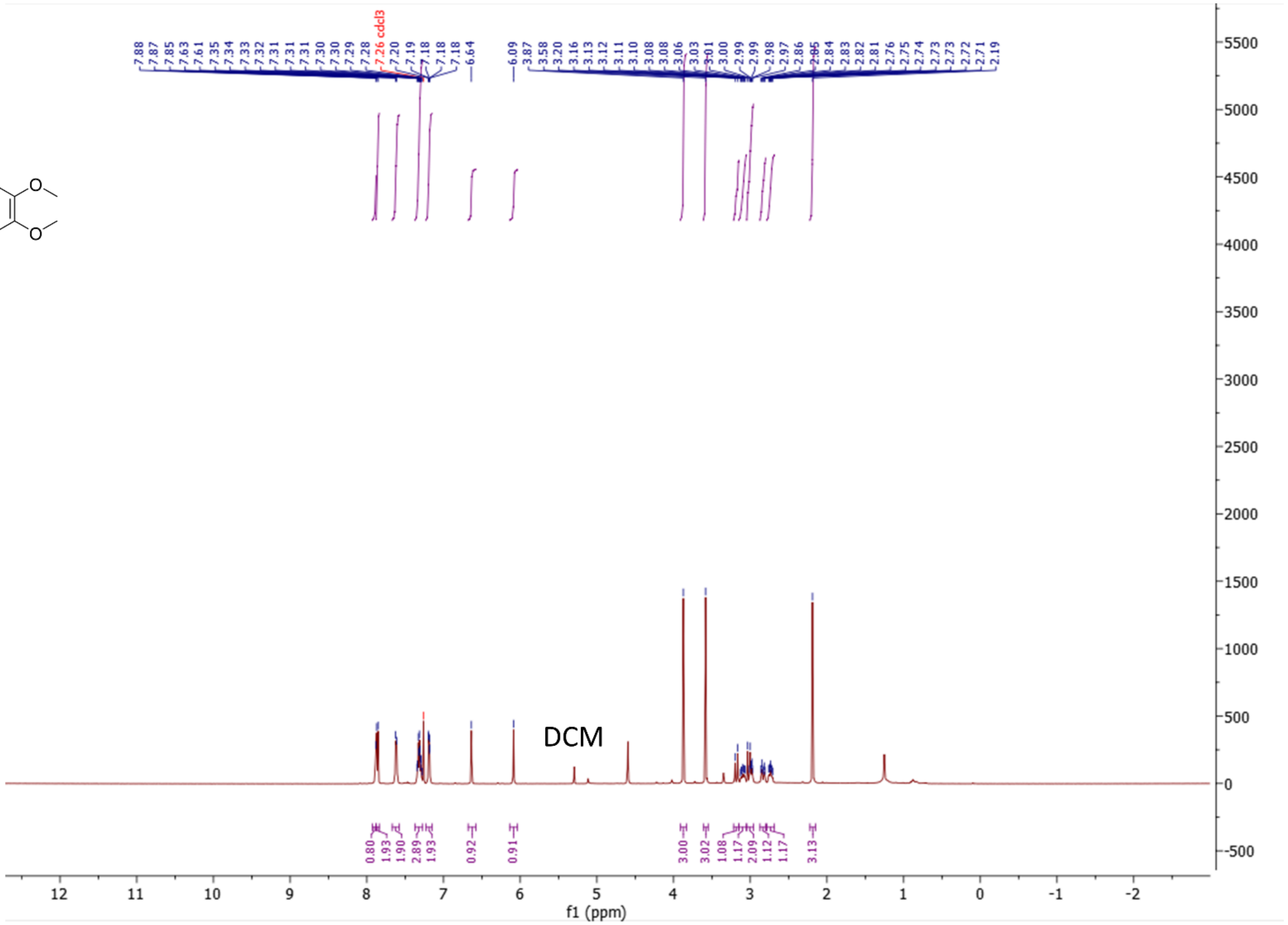

PAWS-24, SZ26TA35

^{1}H NMR (500 MHz) in $(CD_3)_2SO$

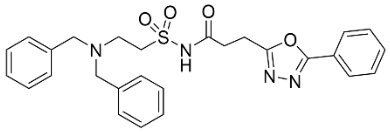

SZ26TA35

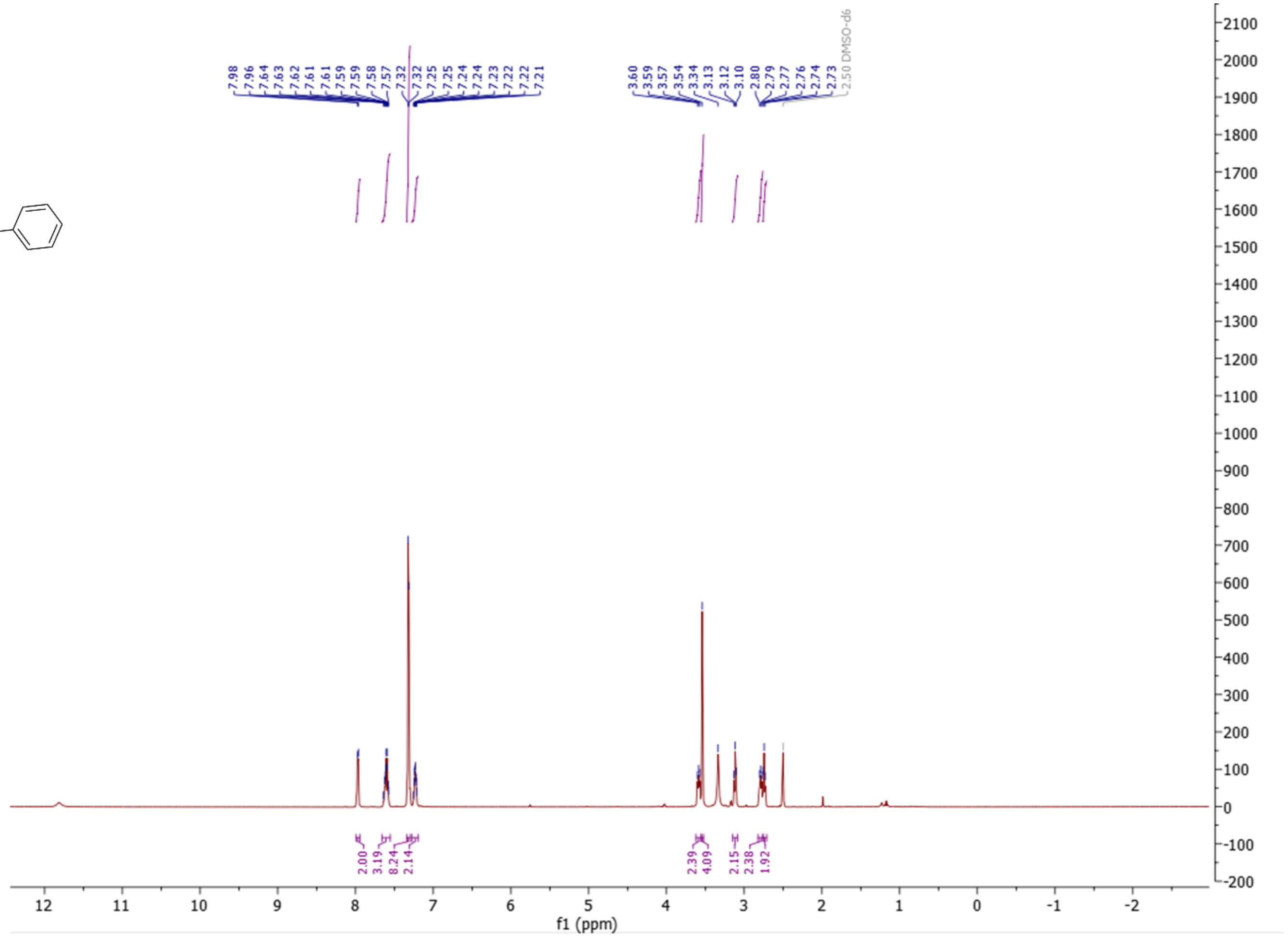

PAWS-25, SZ33TA21

^{1}H NMR (500 MHz) in $(CD_3)_2SO$

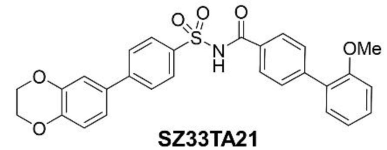

SZ33TA21

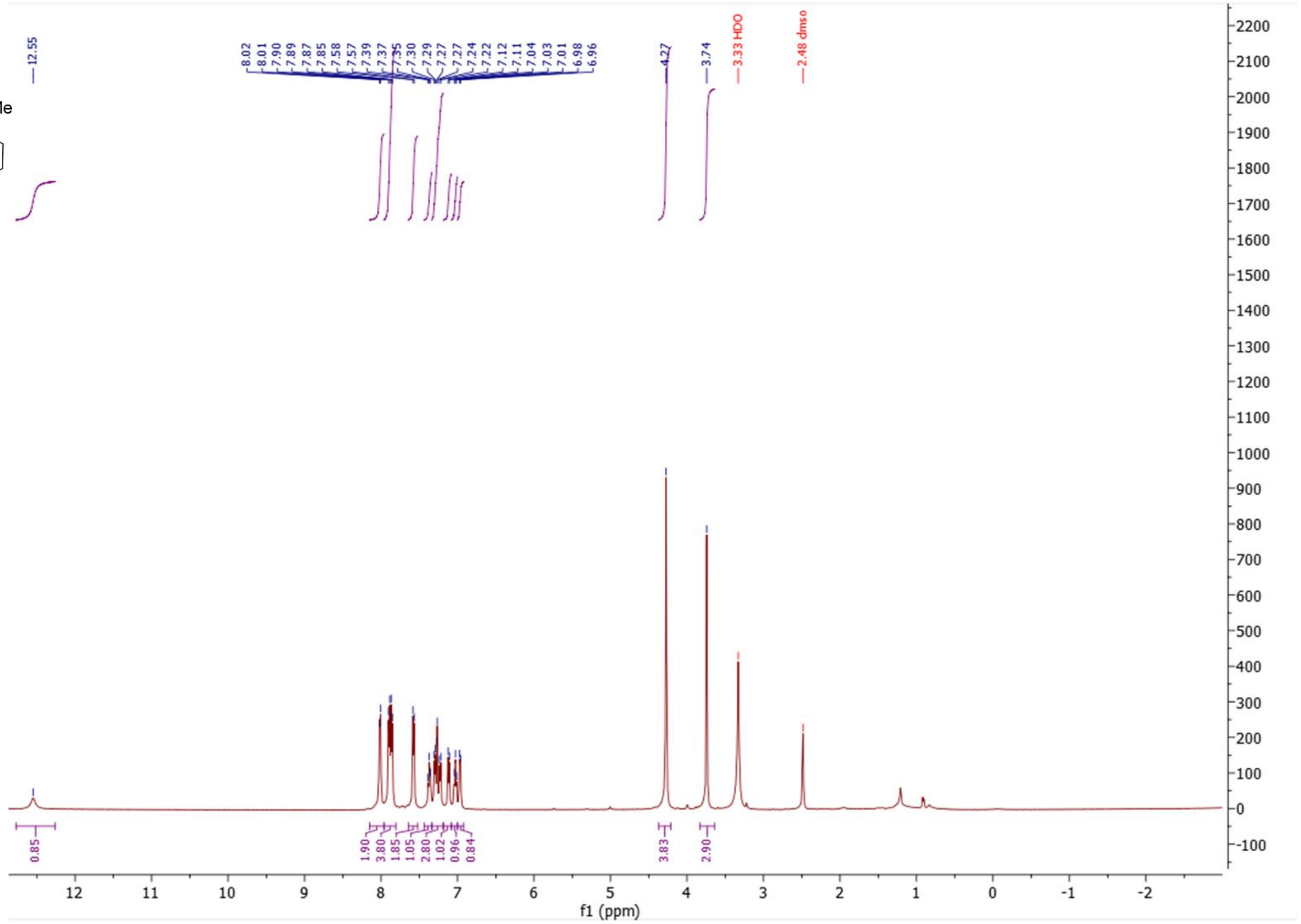

PAWS-26, SZ37TA21

^{1}H NMR (500 MHz) in $(CD_3)_2SO$

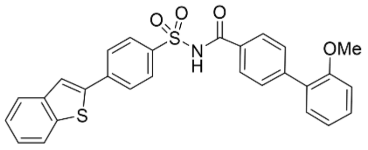

SZ37TA21

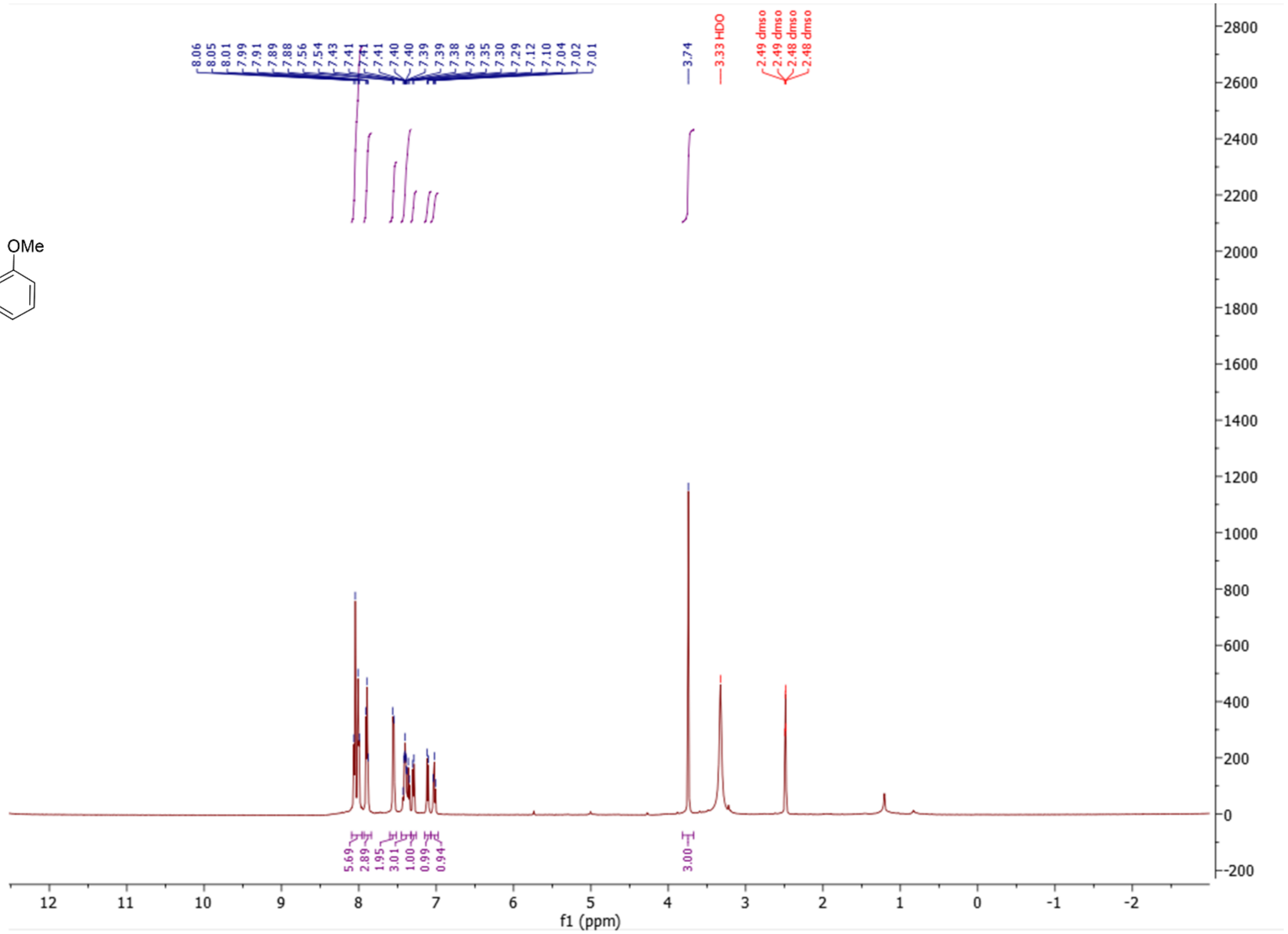

PAWS-27, SZ28TA40

^{1}H NMR (500 MHz) in $(CD_3)_2SO$

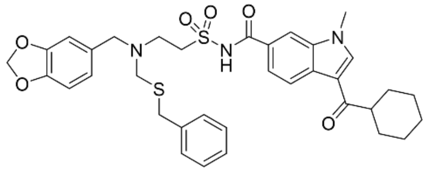

SZ28TA40

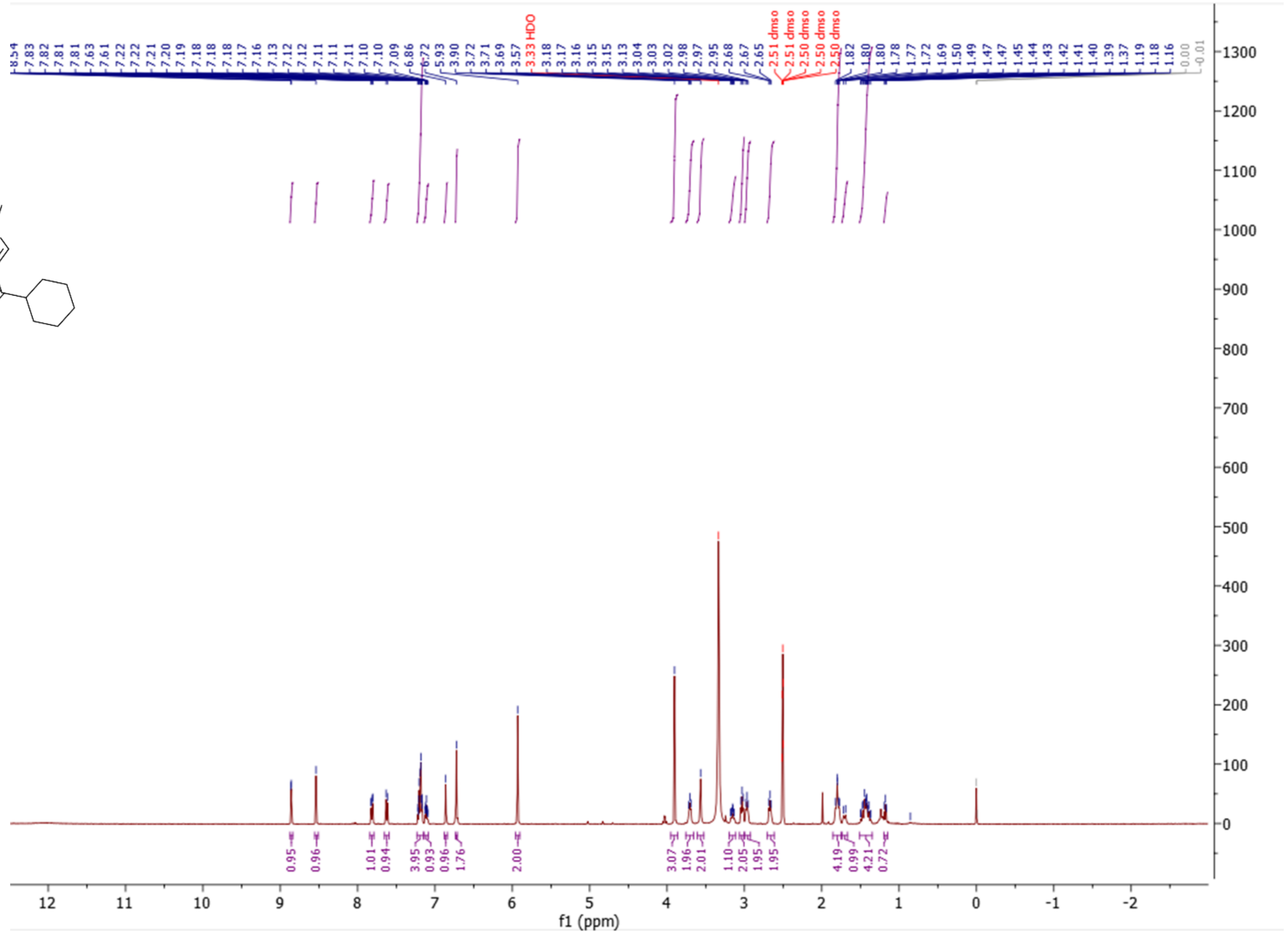

PAWS-28, SZ35TA45

^{1}H NMR (500 MHz) in $(CD_3)_2CO$

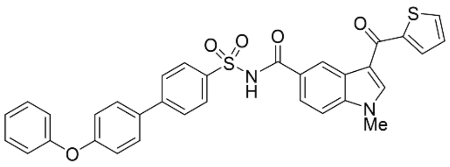

SZ35TA45

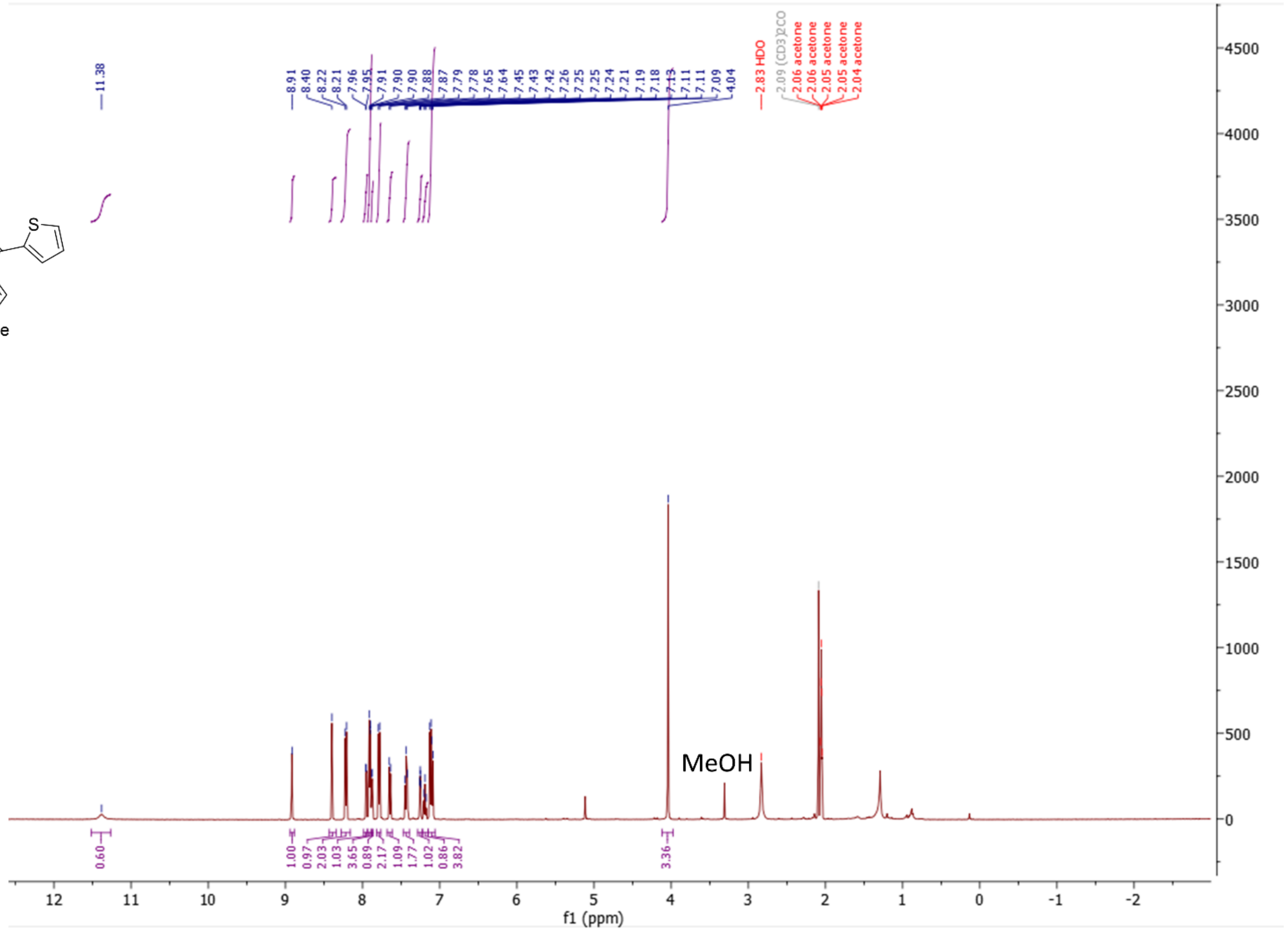

PAWS-29, SZ33TA30

^{1}H NMR (500 MHz) in $(CD_3)_2CO$

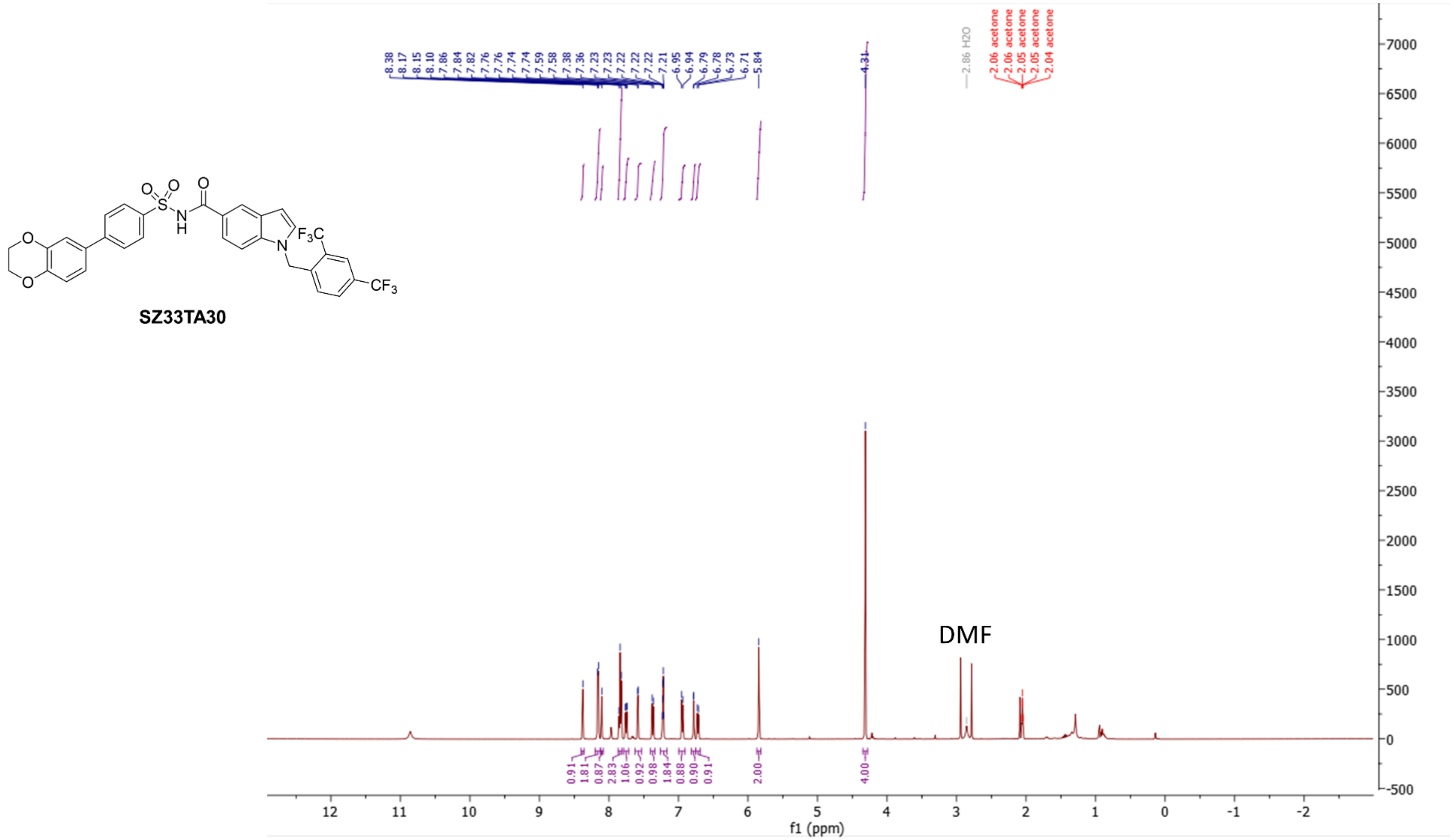

PAWS-30, SZ24TA4

^{1}H NMR (500 MHz) in $CDCl_3$

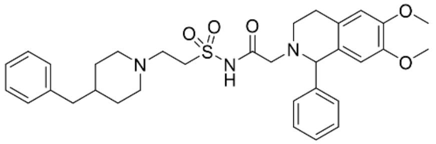

SZ24TA4

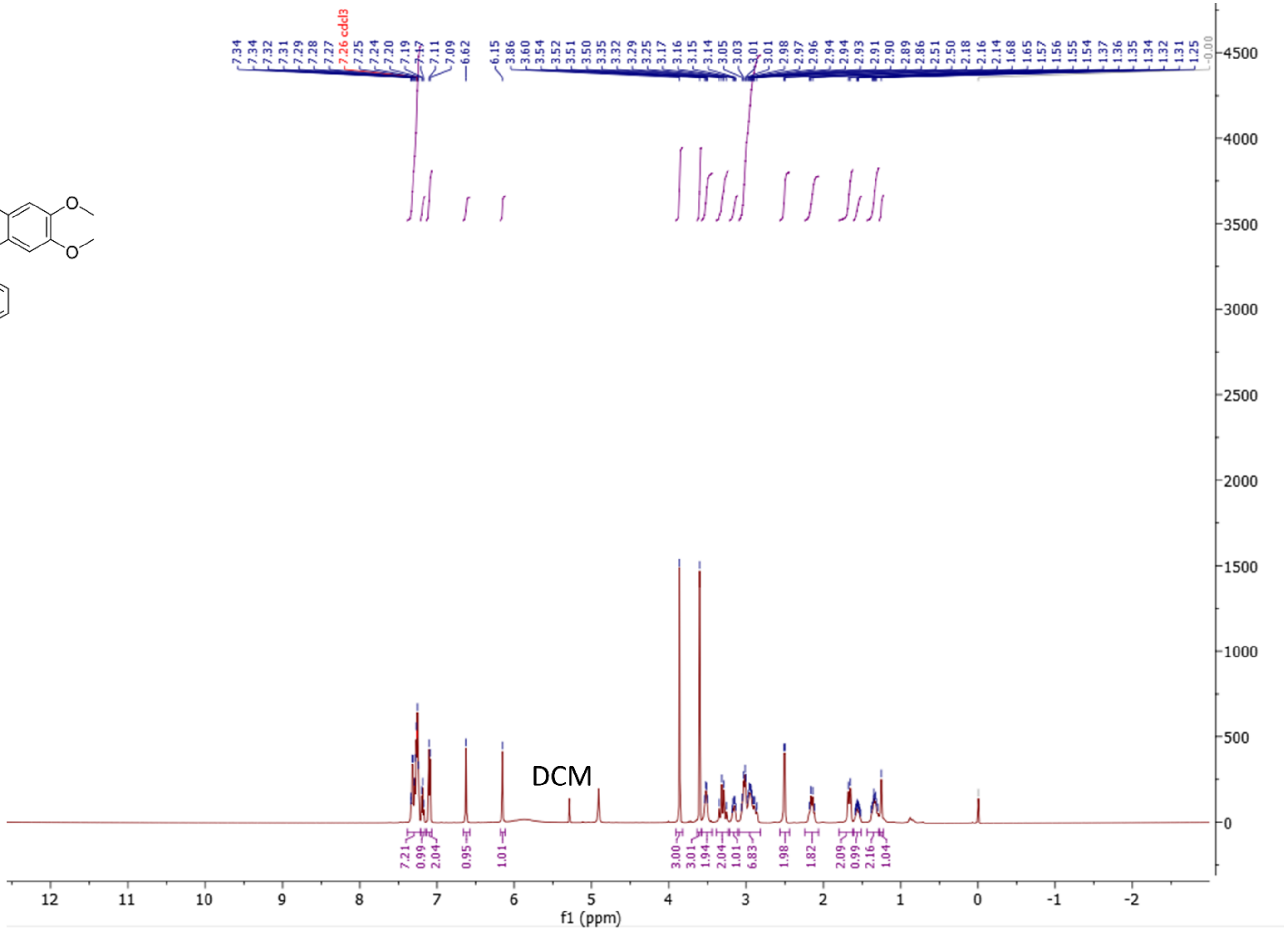

PAWS-31, SZ16TA41

^{1}H NMR (500 MHz) in $(CD_3)_2SO$

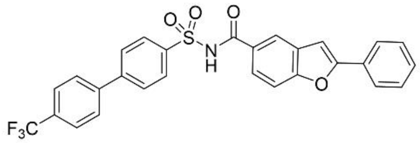

SZ16TA41

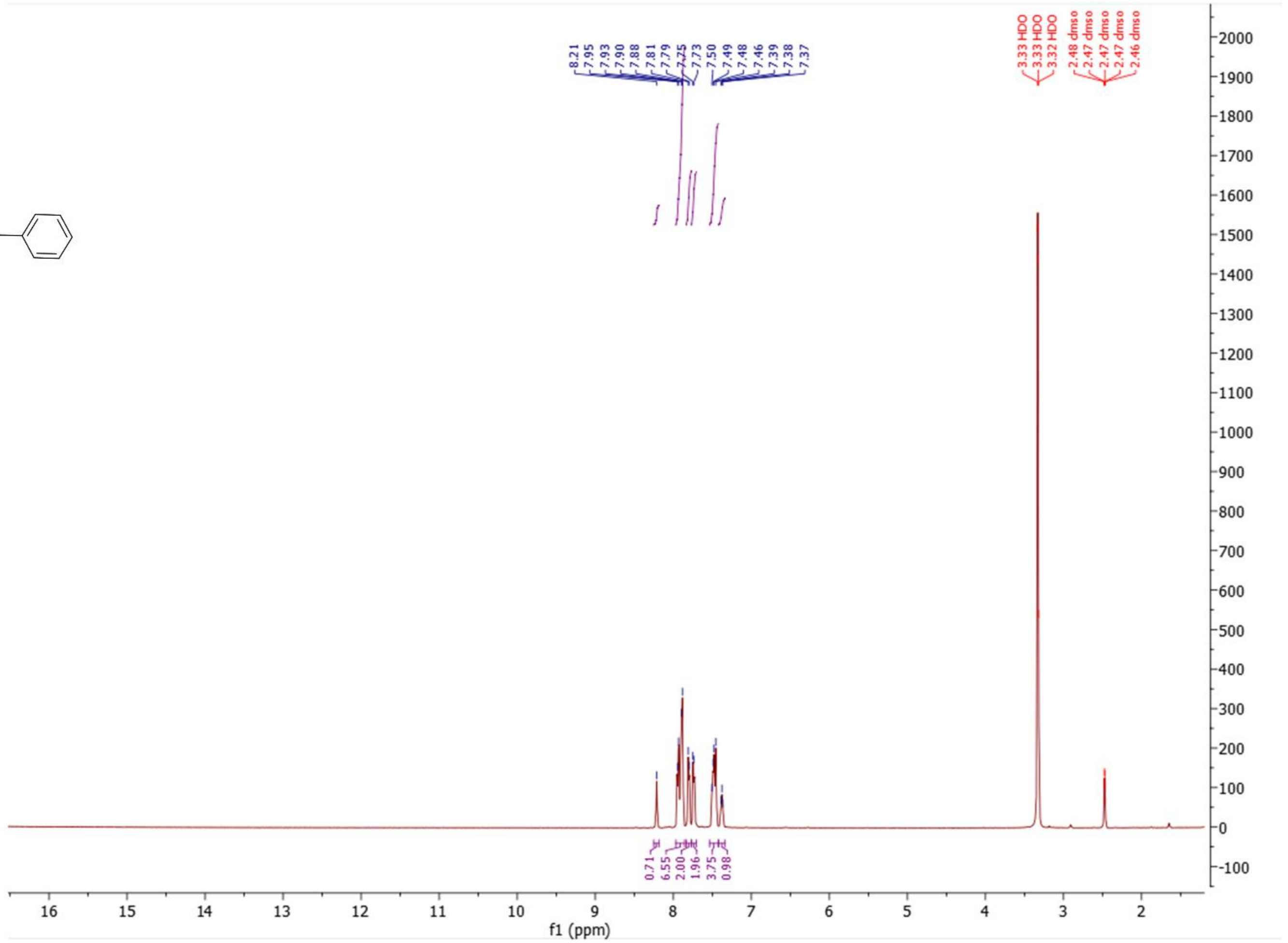

PAWS-32, SZ28TA30

^{1}H NMR (500 MHz) in $CDCl_3$

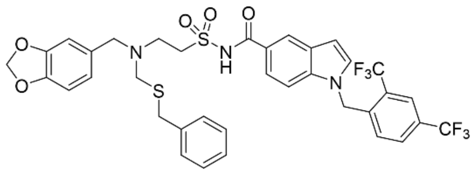

SZ28TA30

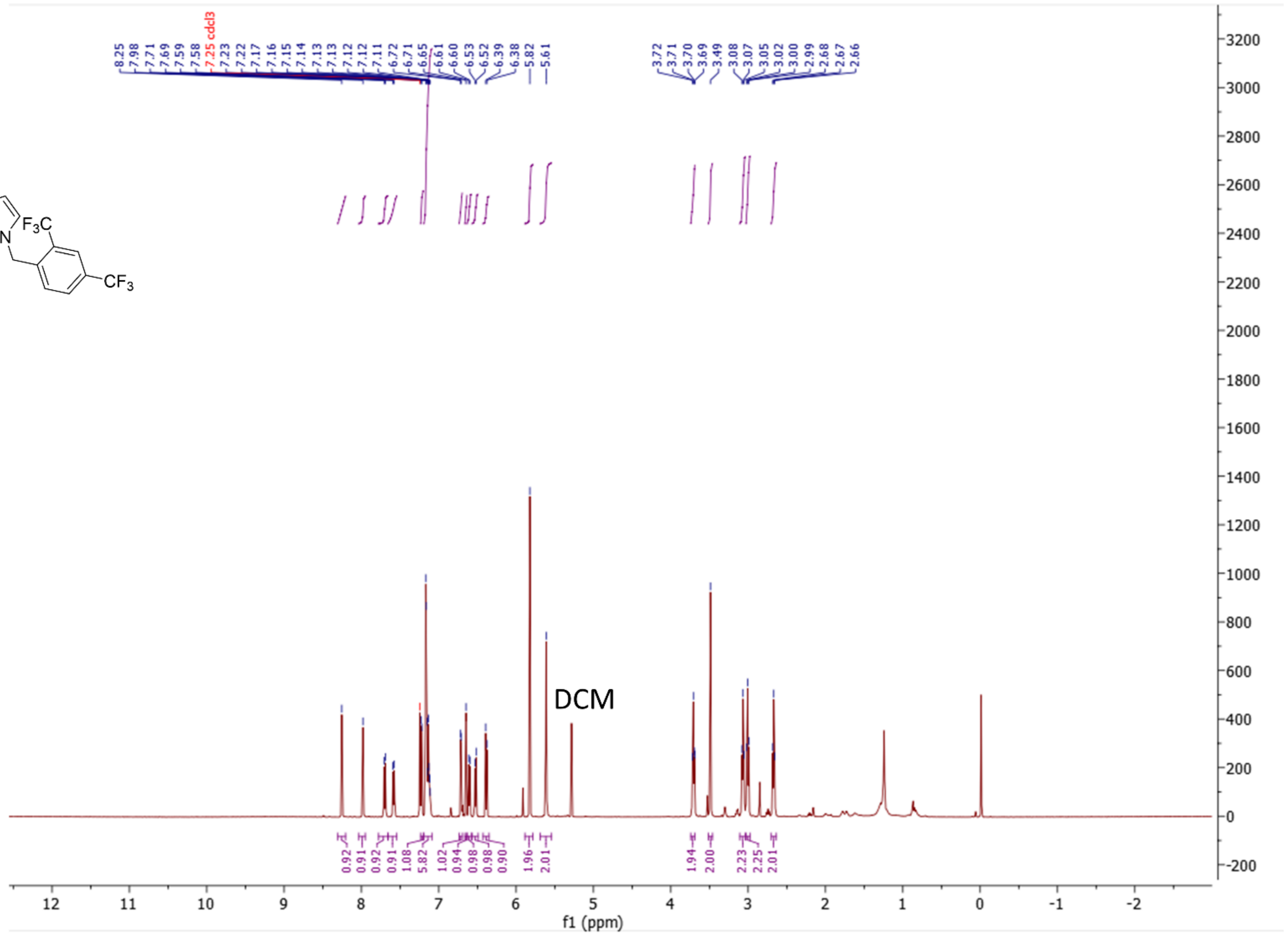

PAWS-33, SZ22TA41

^{1}H NMR (400 MHz) in $CDCl_3$

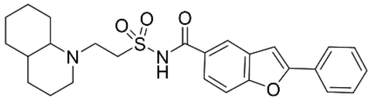

SZ22TA41

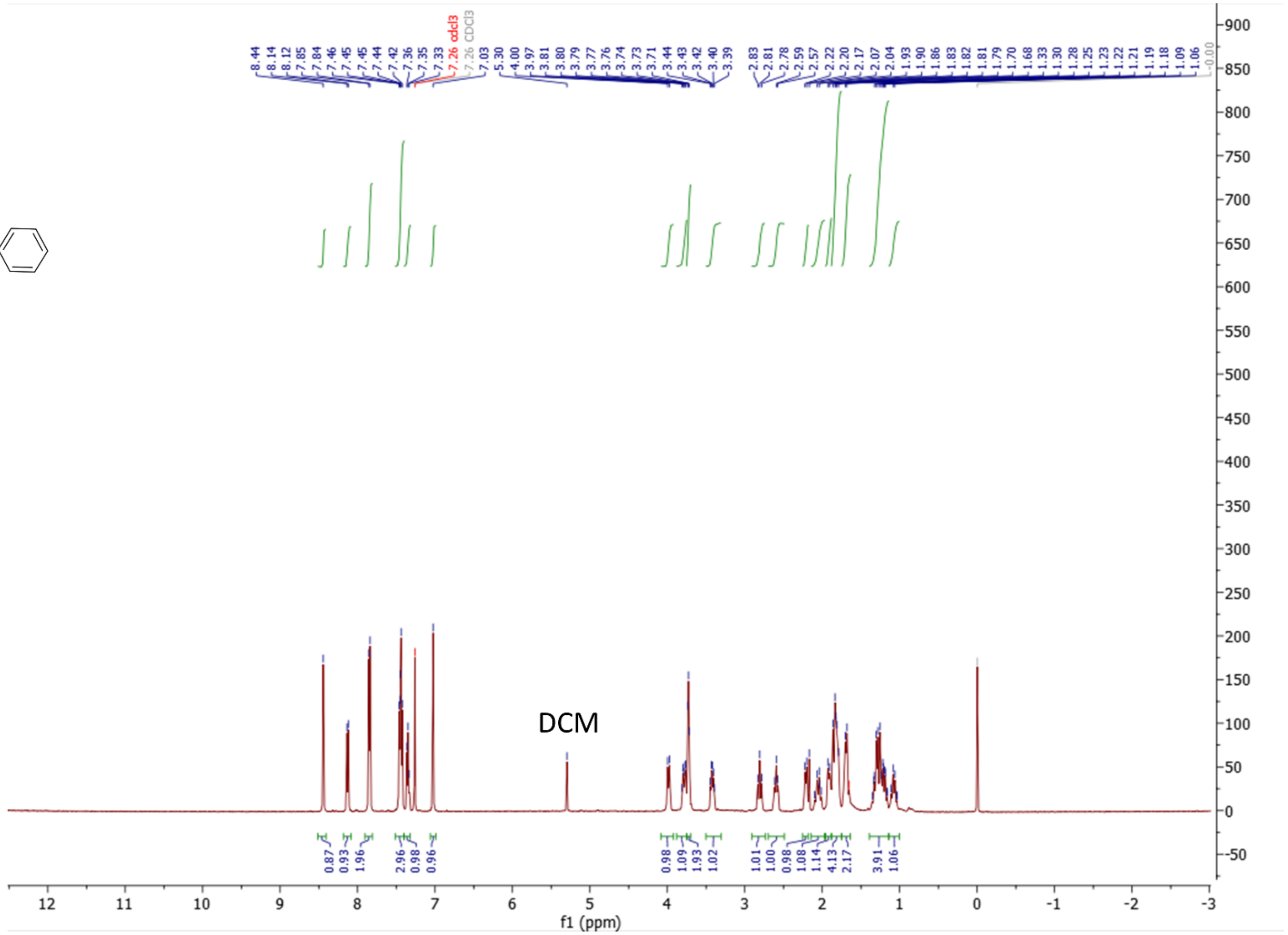

PAWS-34, SZ35TA41

^{1}H NMR (400 MHz) in $(CD_3)_2SO$

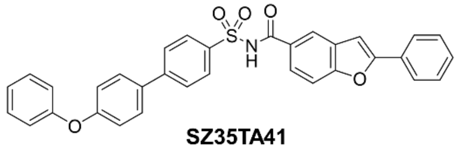

SZ35TA41

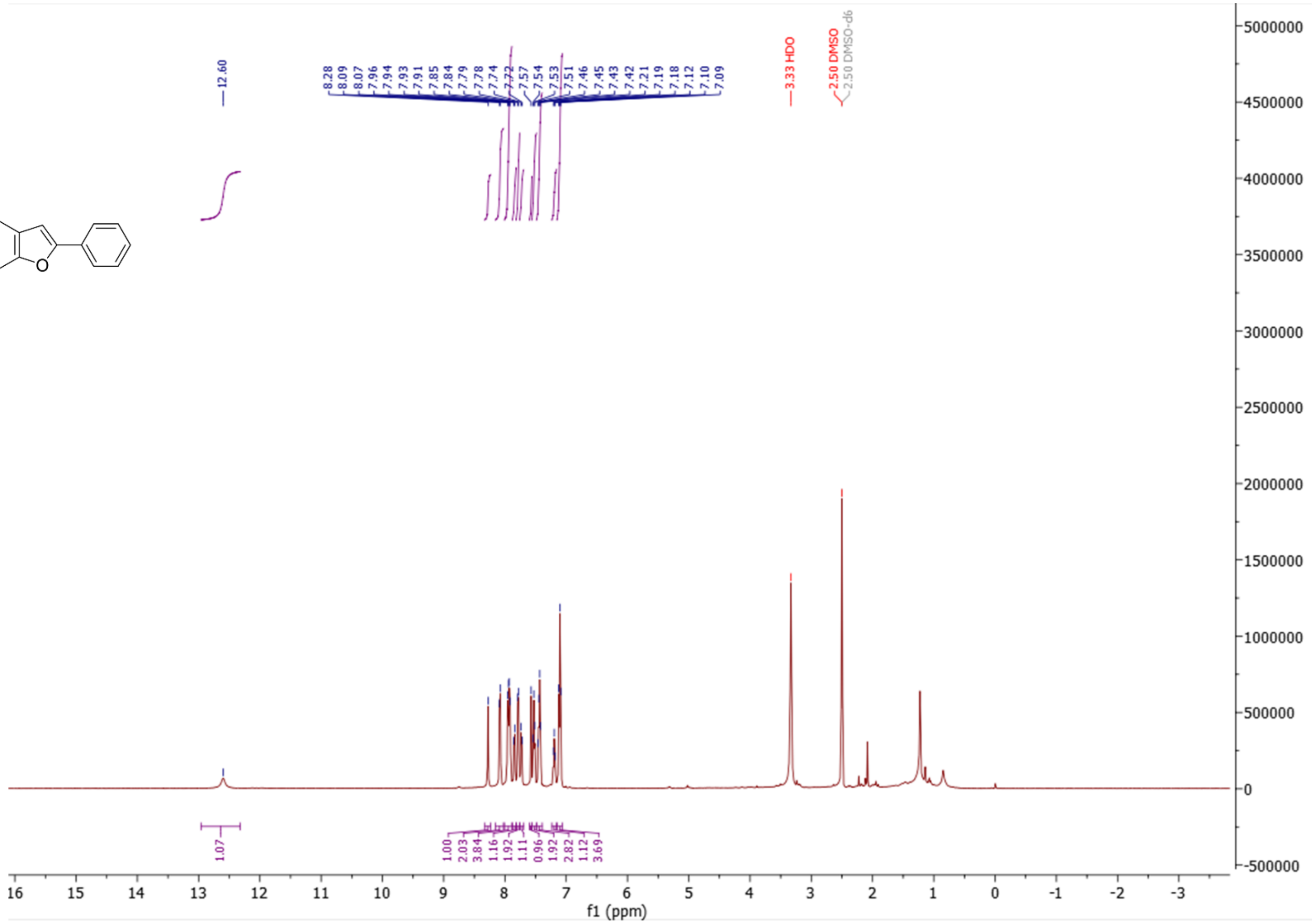

PAWS-35, SZ19TA41

^{1}H NMR (500 MHz) in $(CD_3)_2SO$

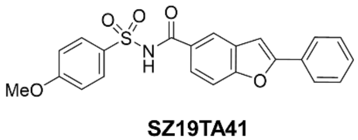
SZ19TA41

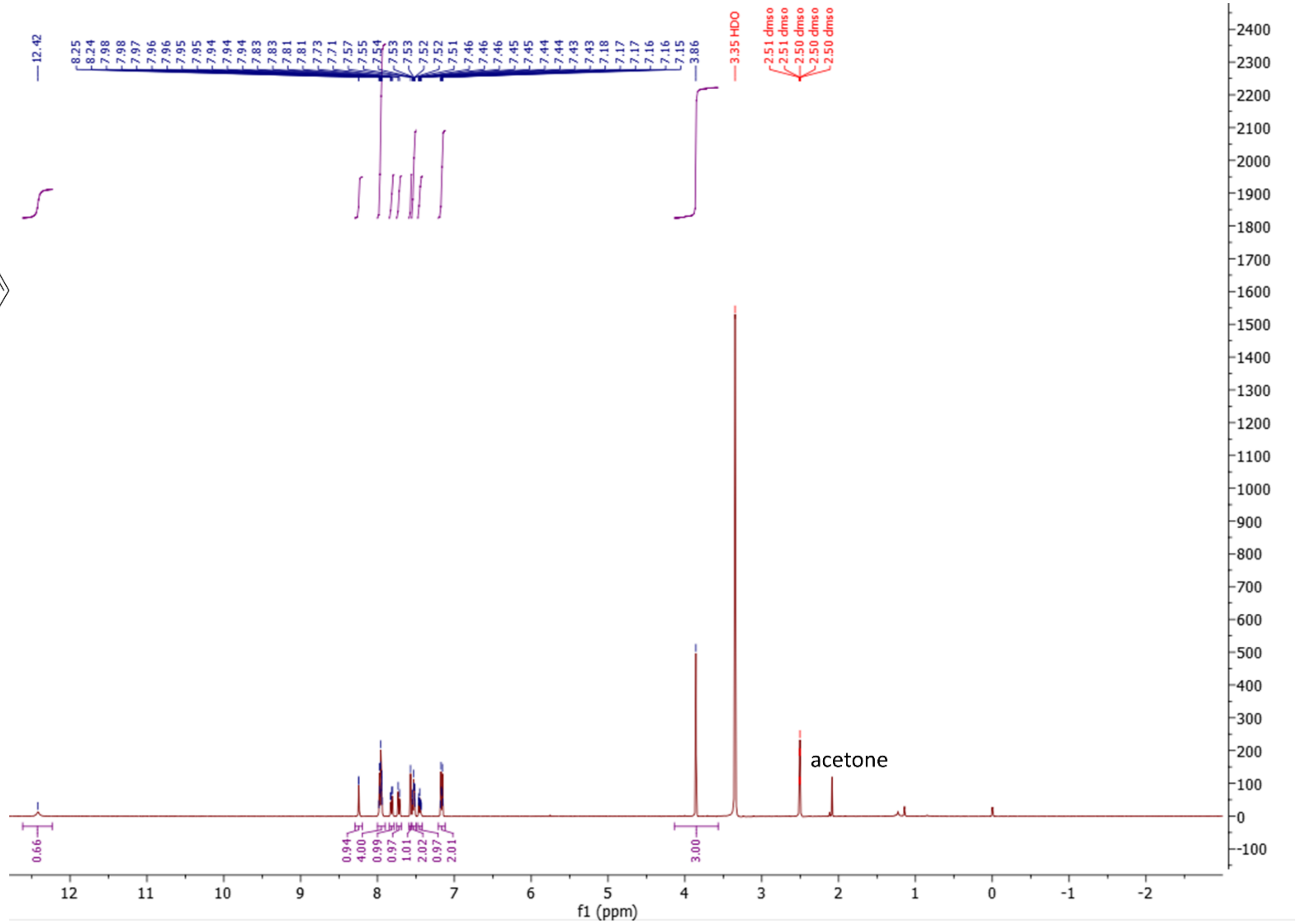

PAWS-36, SZ35TA17

^{1}H NMR (500 MHz) in $CDCl_3$

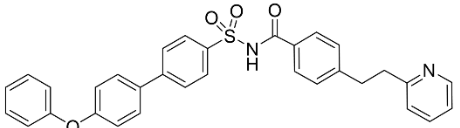
SZ35TA17

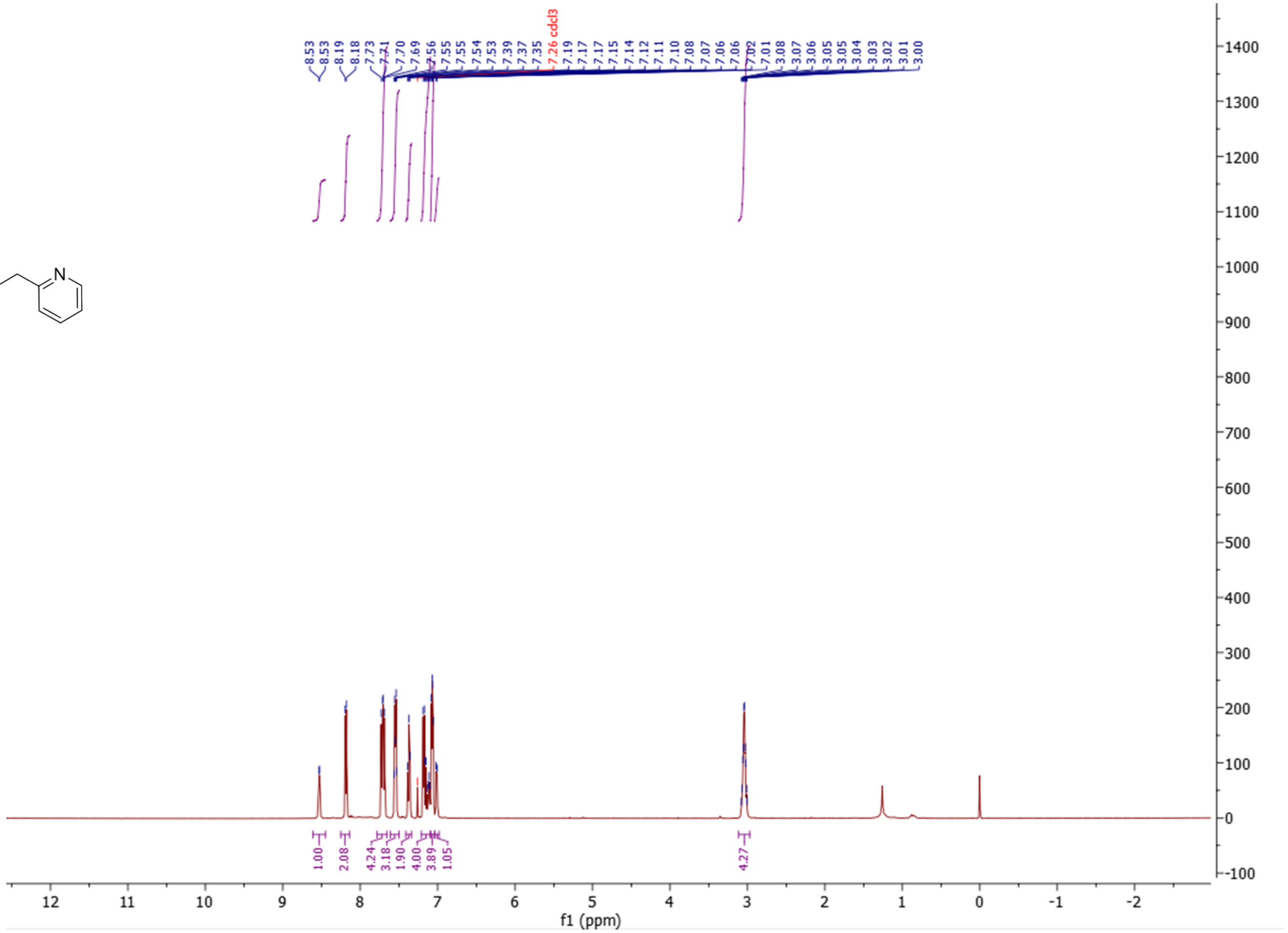

PAWS-37, SZ13TA45

^{1}H NMR (500 MHz) in $(CD_3)_2SO$

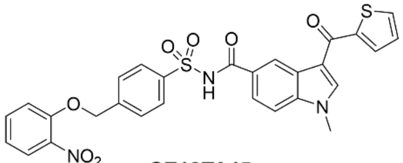

SZ13TA45

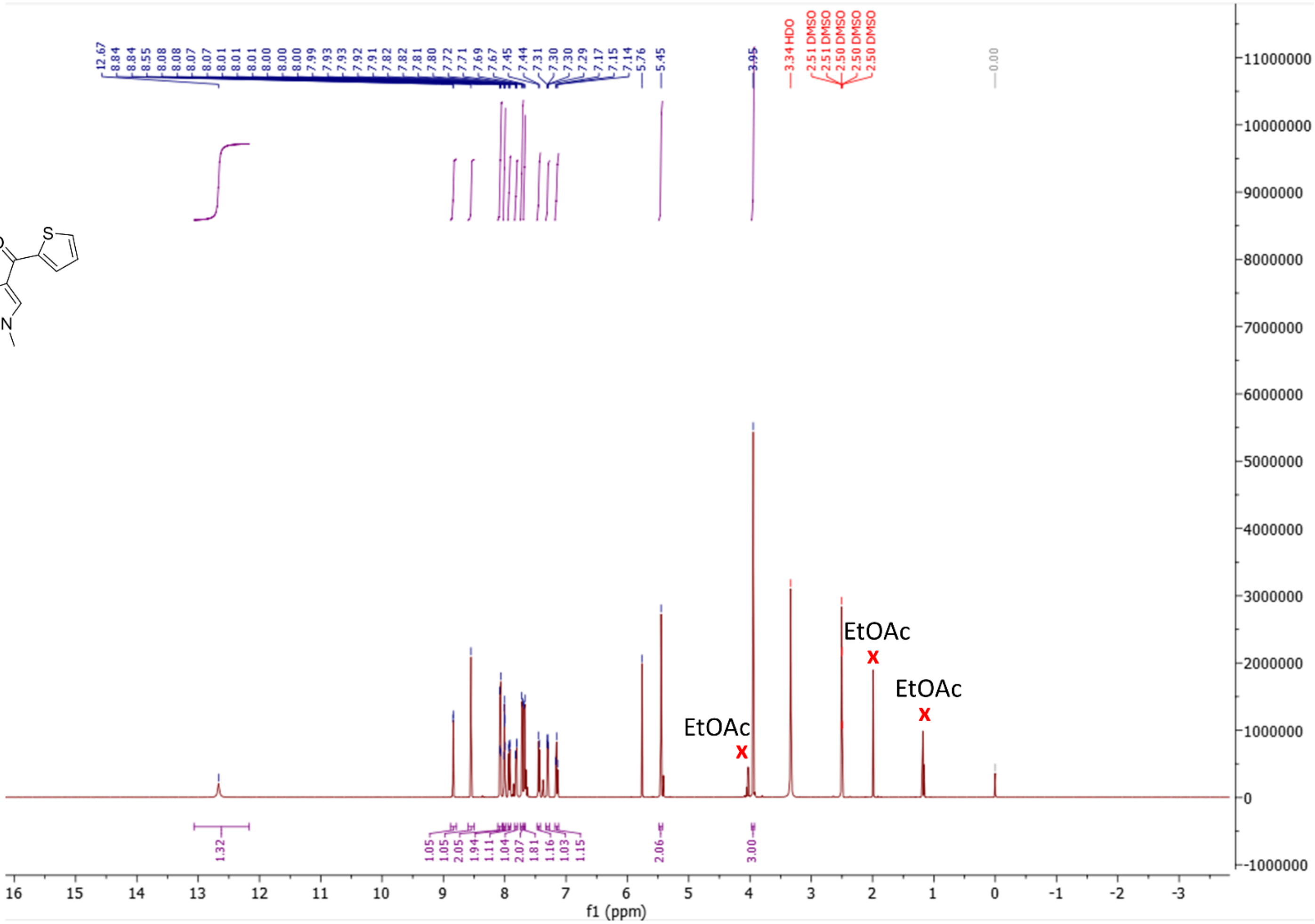

PAWS-38, SZ25TA30

^{1}H NMR (500 MHz) in $CDCl_3$

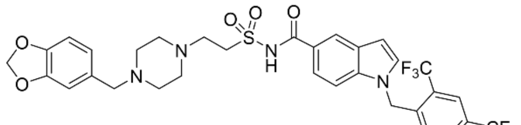

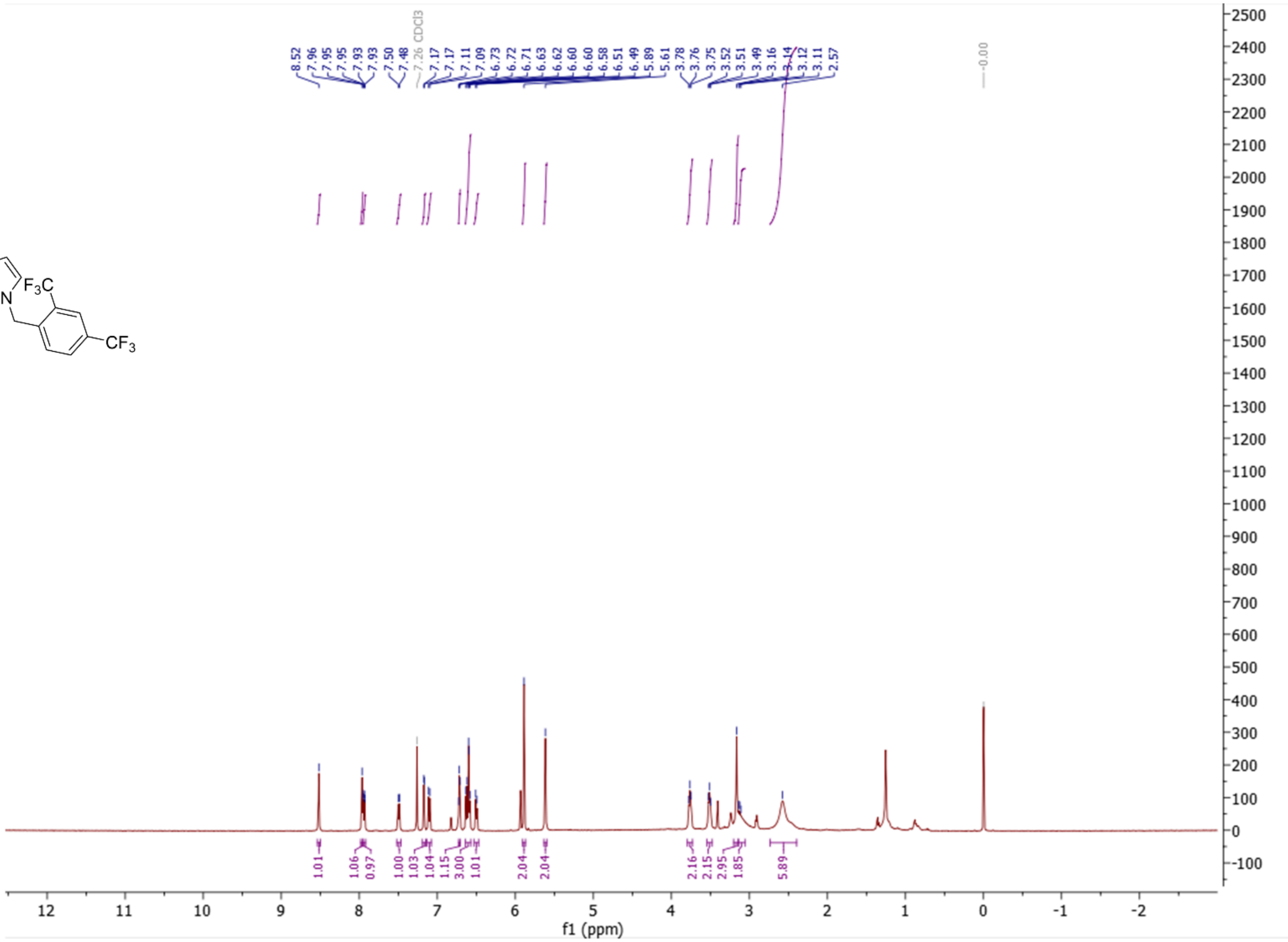

PAWS-39, SZ38TA21

^{1}H NMR (500 MHz) in $CDCl_3$

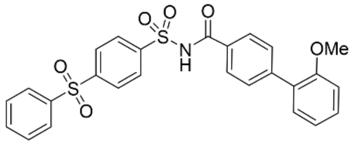

SZ38TA21

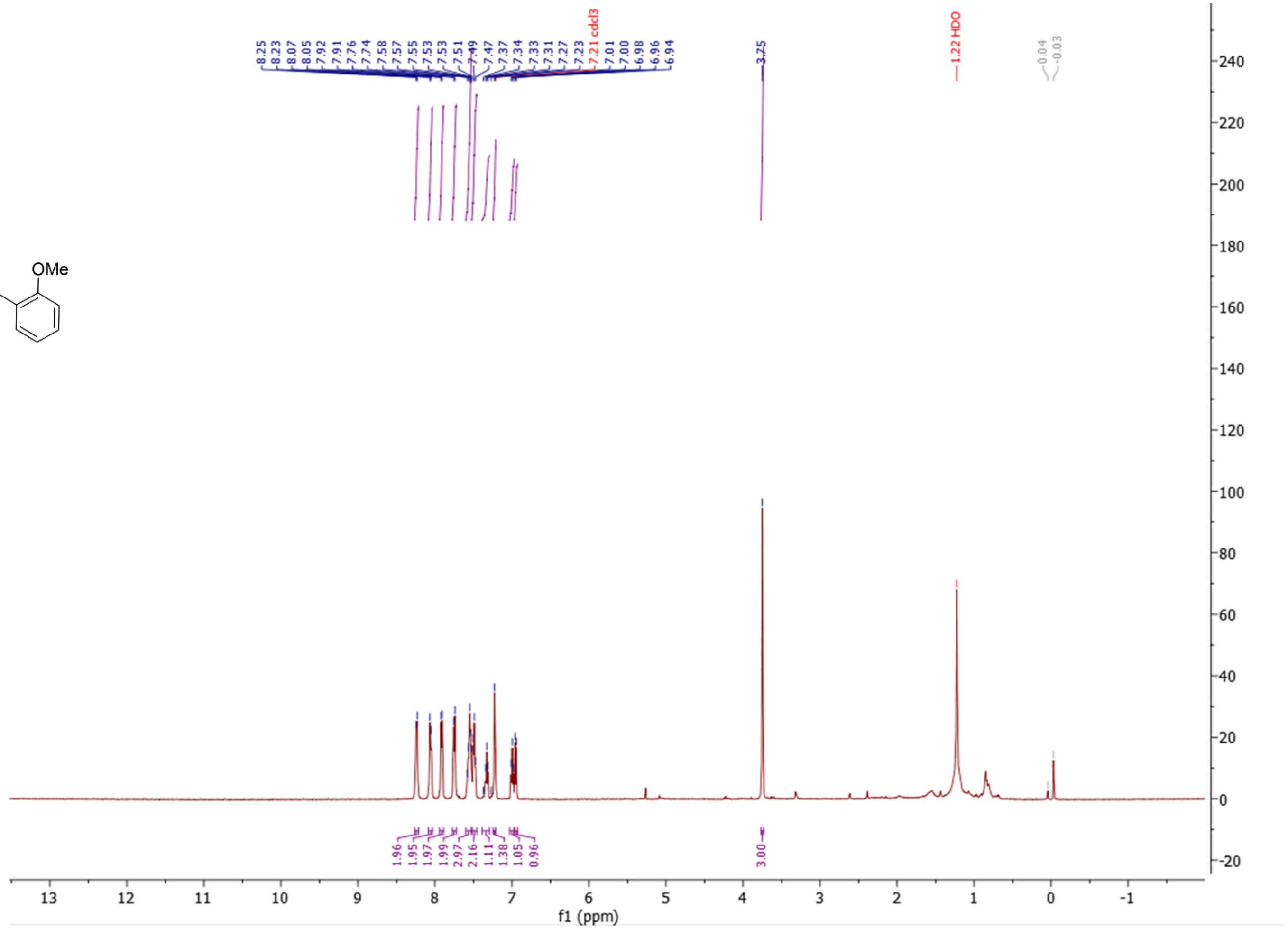

PAWS-40, SZ34TA25

^{1}H NMR (400 MHz) in $CDCl_3$

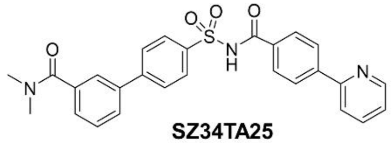

SZ34TA25

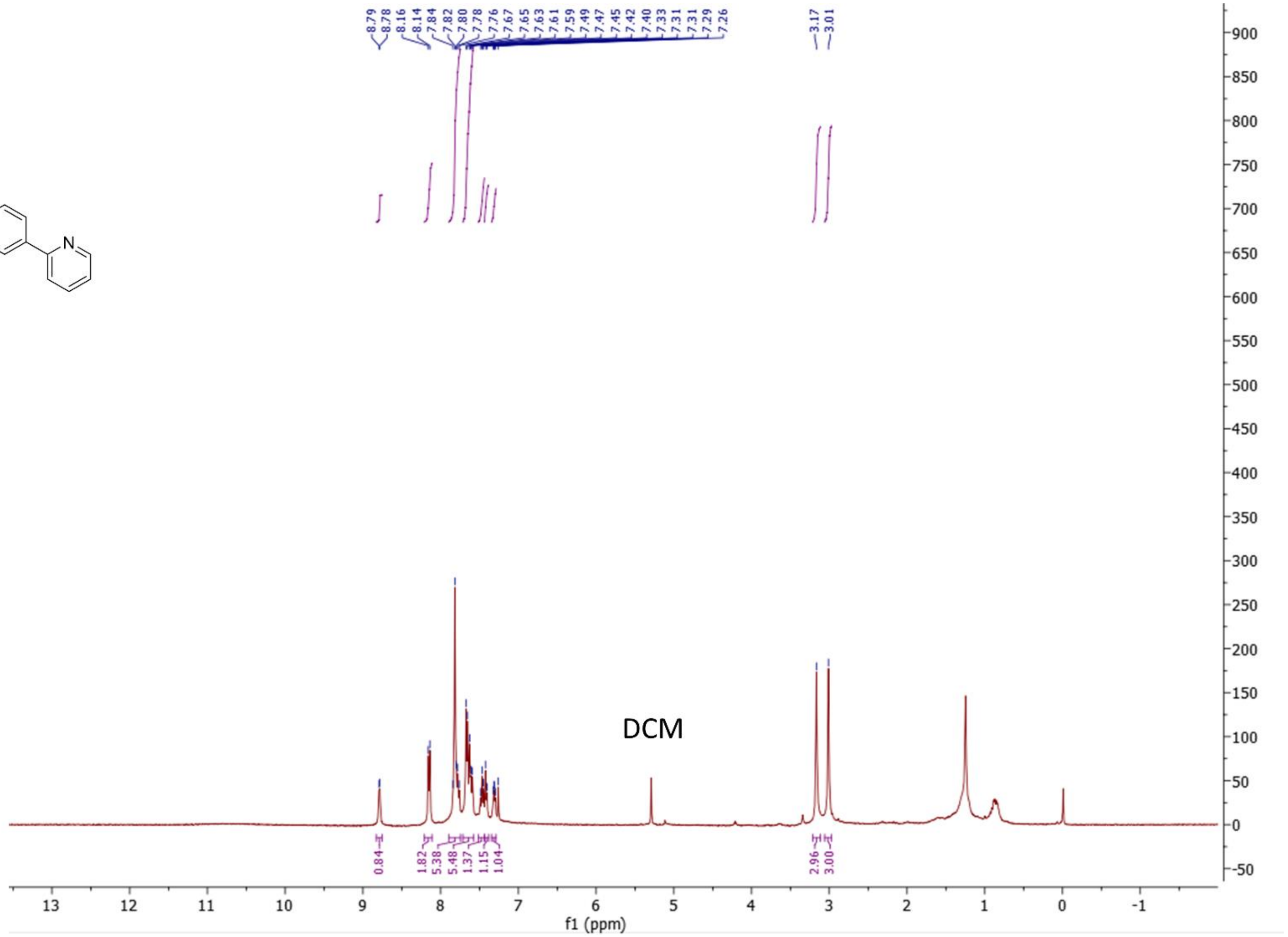

PAWS-41, SZ28TA26

^{1}H NMR (500 MHz) in $(CD_3)_2SO$

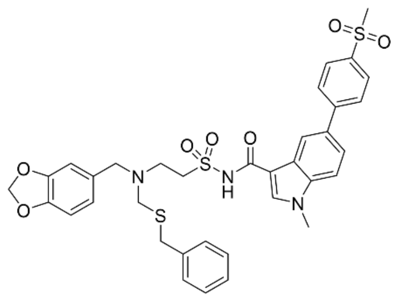

SZ28TA26

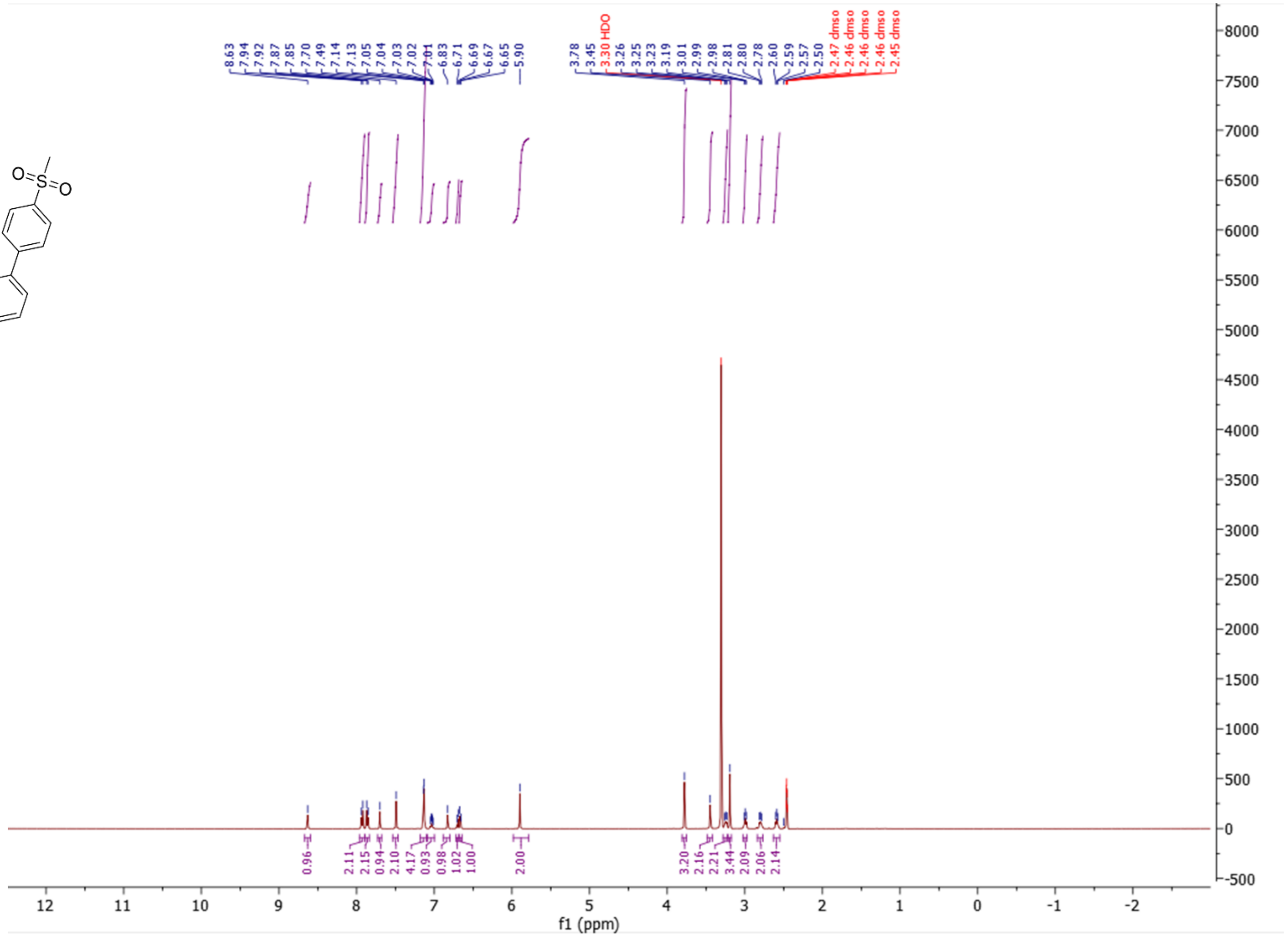

PAWS-42, SZ28TA41

^{1}H NMR (400 MHz) in $CDCl_3$

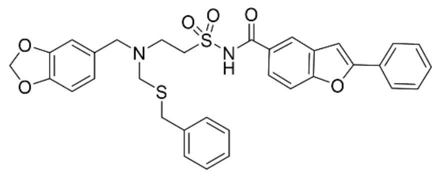

SZ28TA41

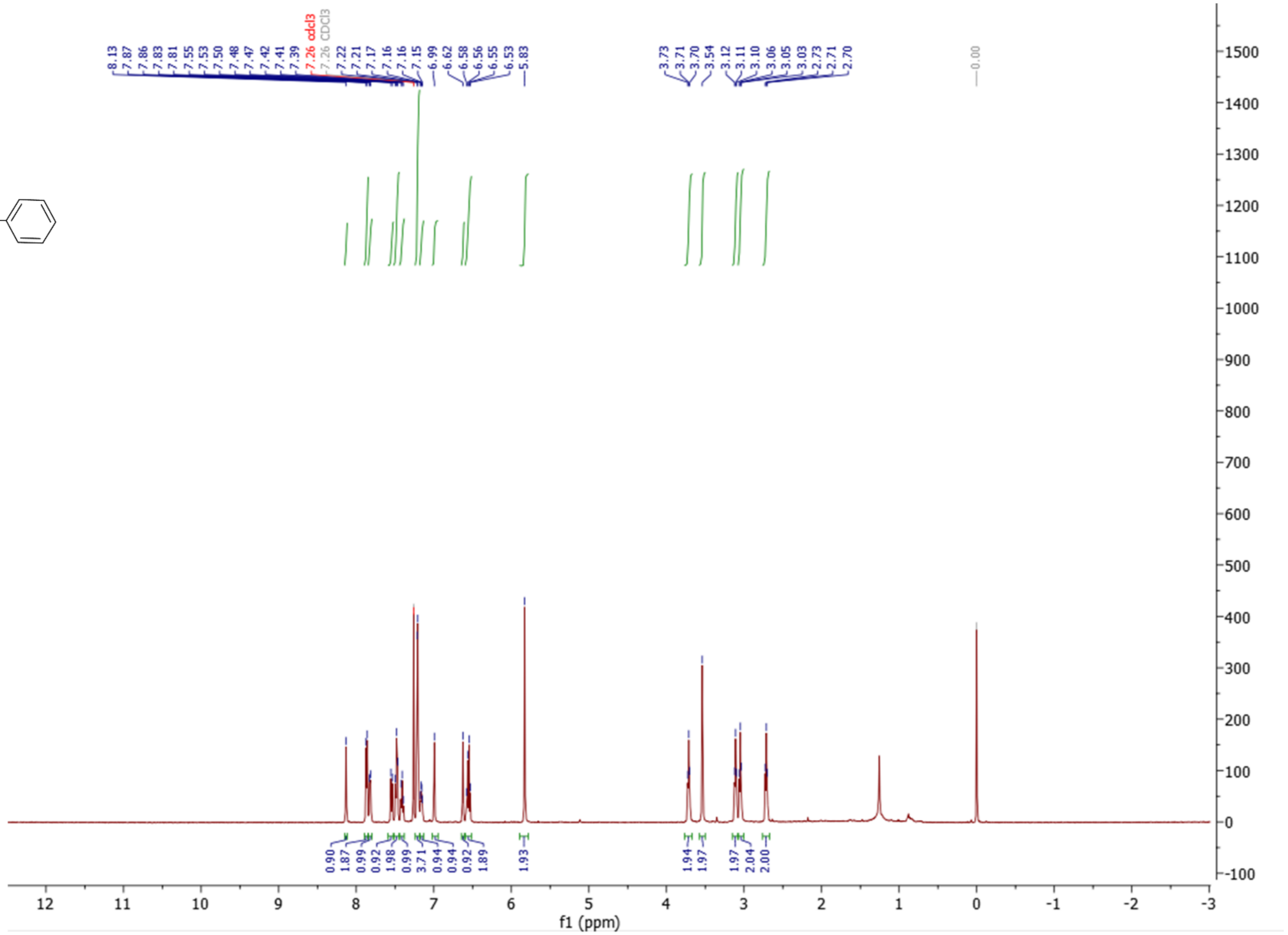

PAWS-A1, SZ(Me)TA30

^{1}H NMR (400 MHz) in $(CD_3)_2CO$

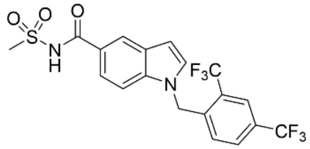

SZ(Me)TA30

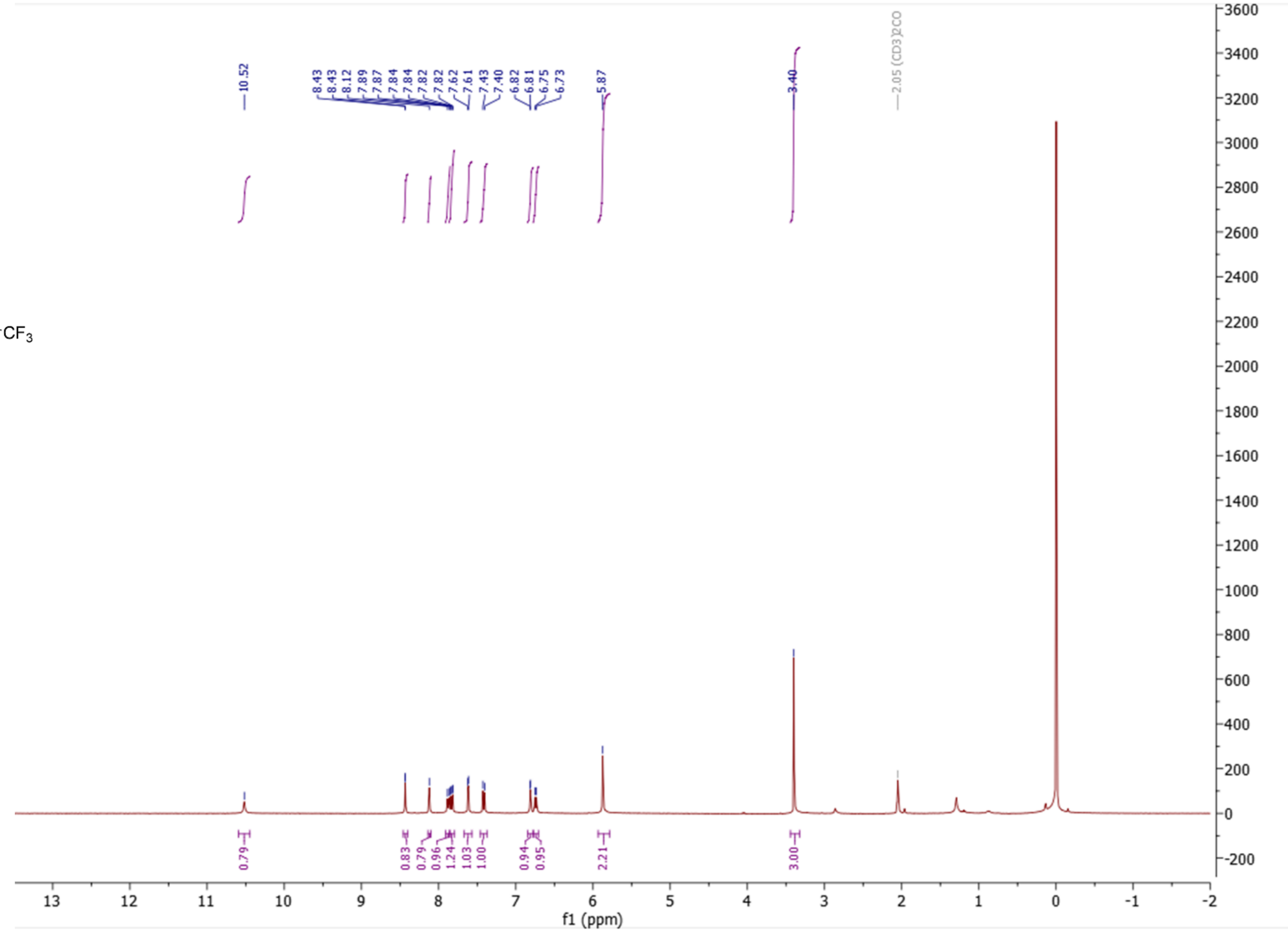

PAWS-A2, SZ(Me)TA21

^{1}H NMR (500 MHz) in $(CD_3)_2SO$

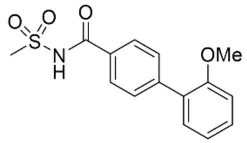

SZ(Me)TA21

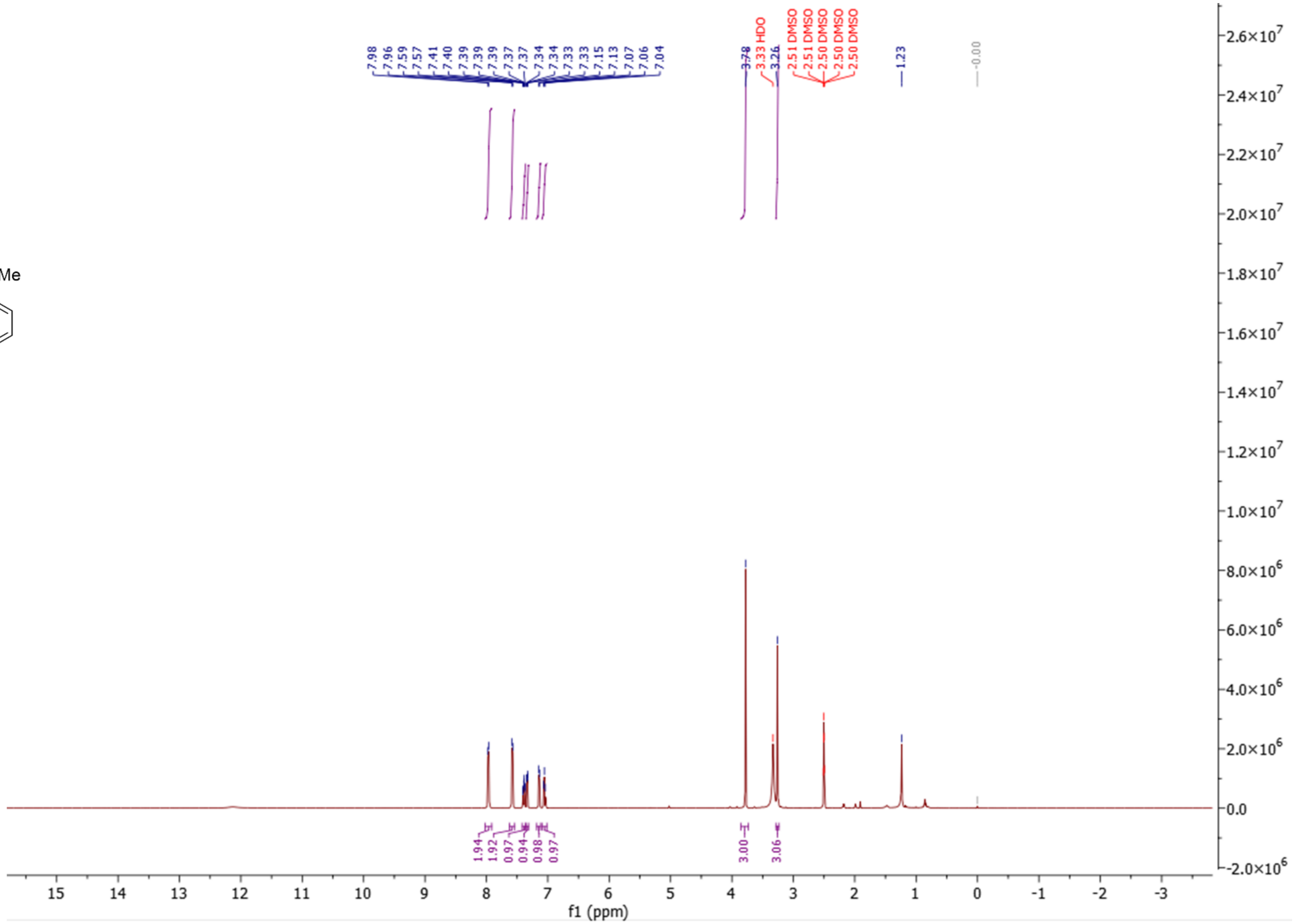

PAWS-A3, SZ(Me)TA45

^{1}H NMR (500 MHz) in $(CD_3)_2SO$

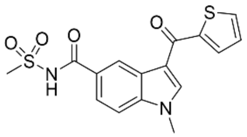

SZ(Me)TA45

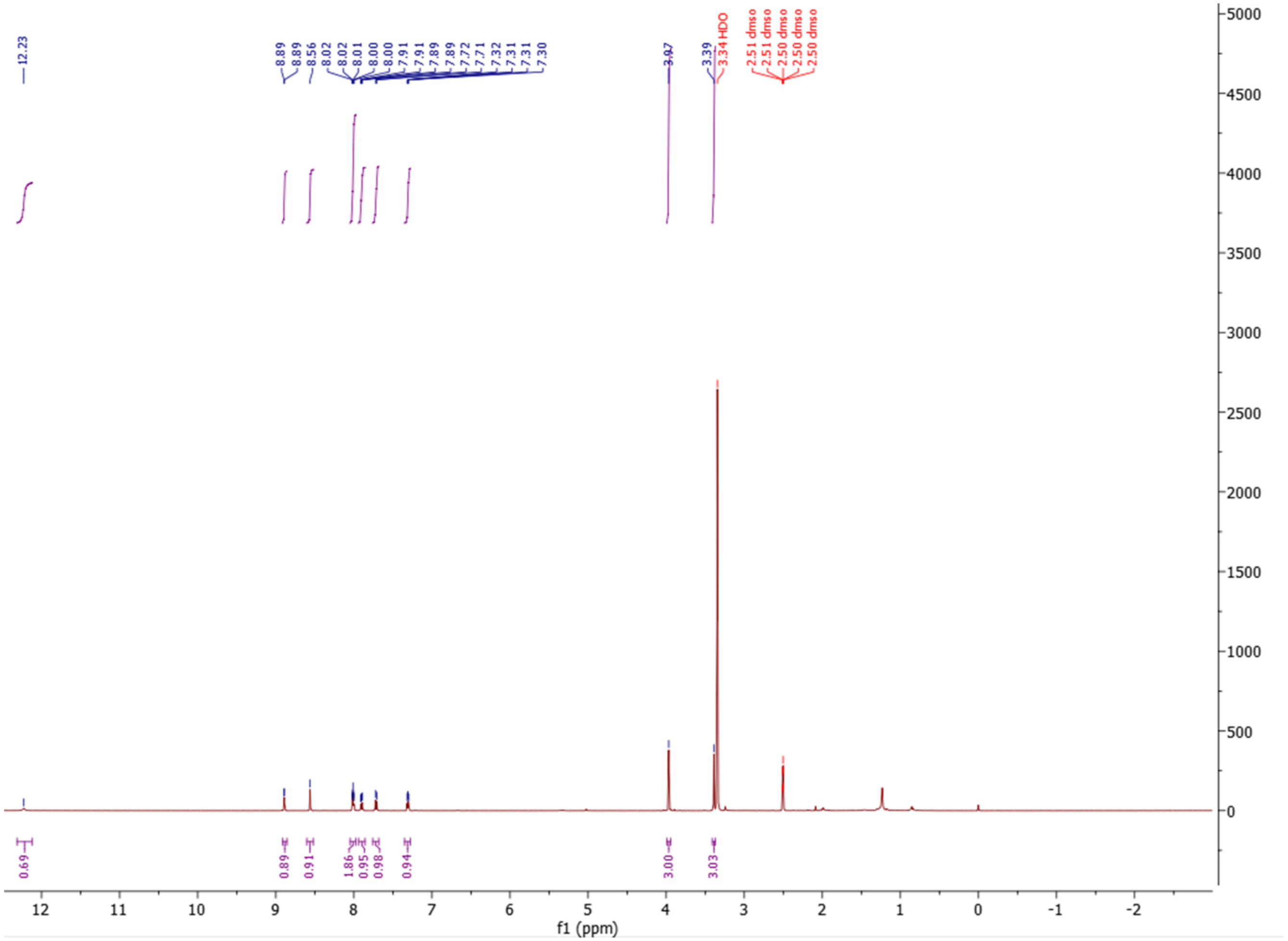

PAWS-A4, SZ(Me)TA41

^{1}H NMR (500 MHz) in $(CD_3)_2SO$

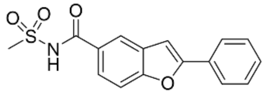

SZ(Me)TA41

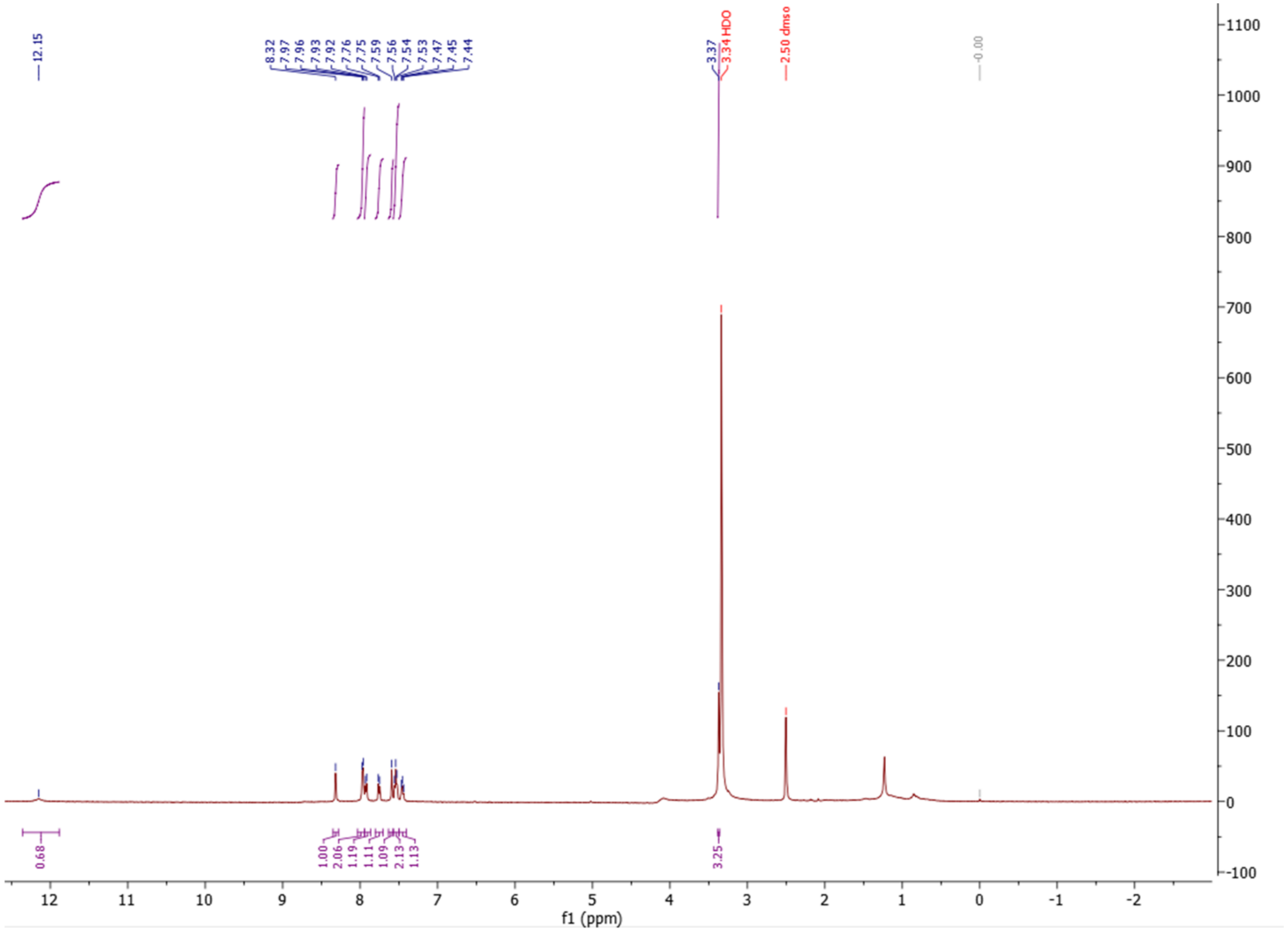

PAWS-A5, SZ(Me)TA5

^{1}H NMR (500 MHz) in $(CD_3)_2SO$

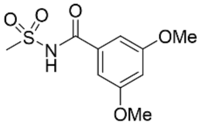

SZ(Me)TA5

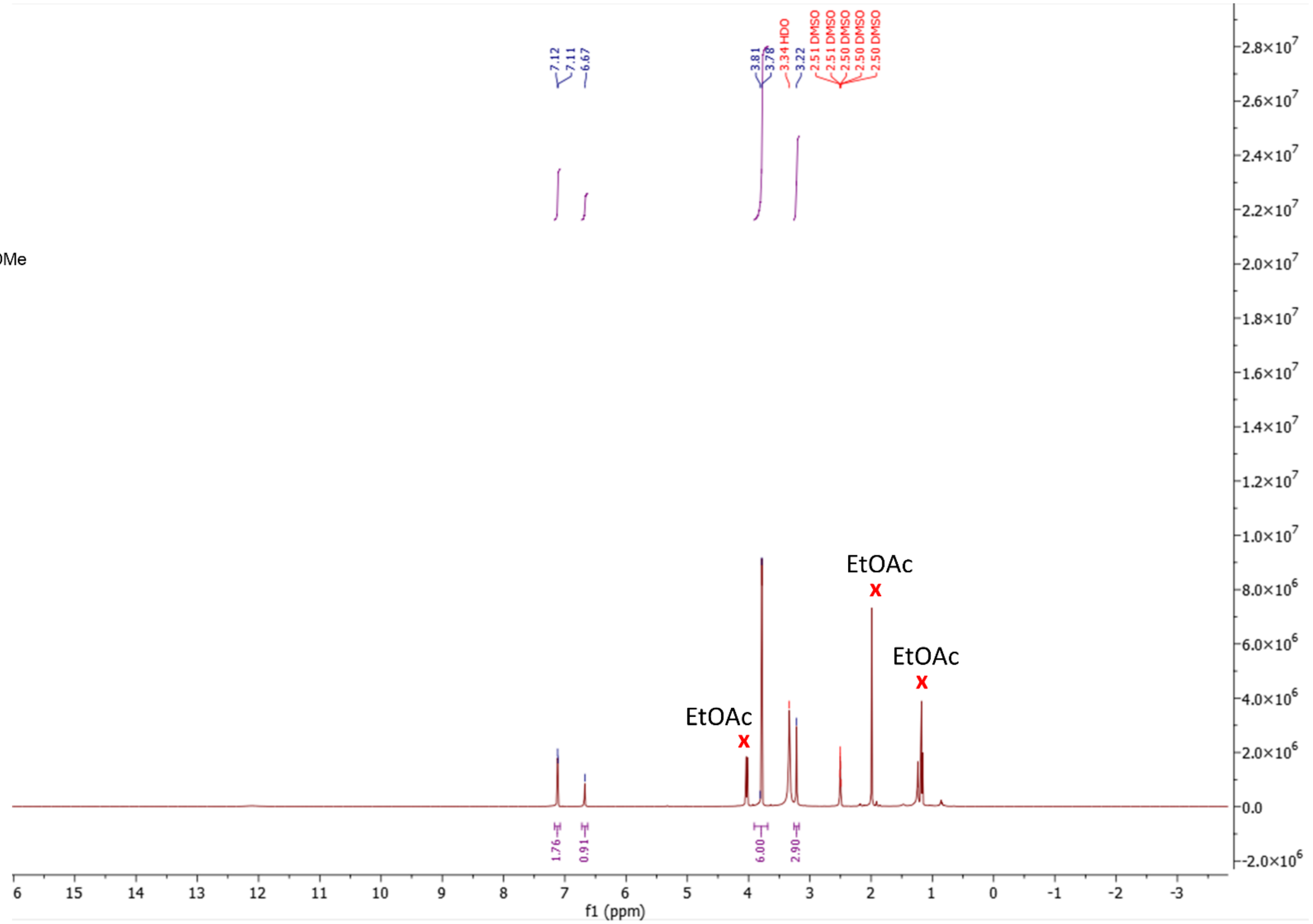

PAWS-A6, SZ33TA(Me)

^{1}H NMR (400 MHz) in $(CD_3)_2CO$

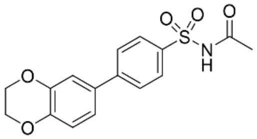

SZ33TA(Me)

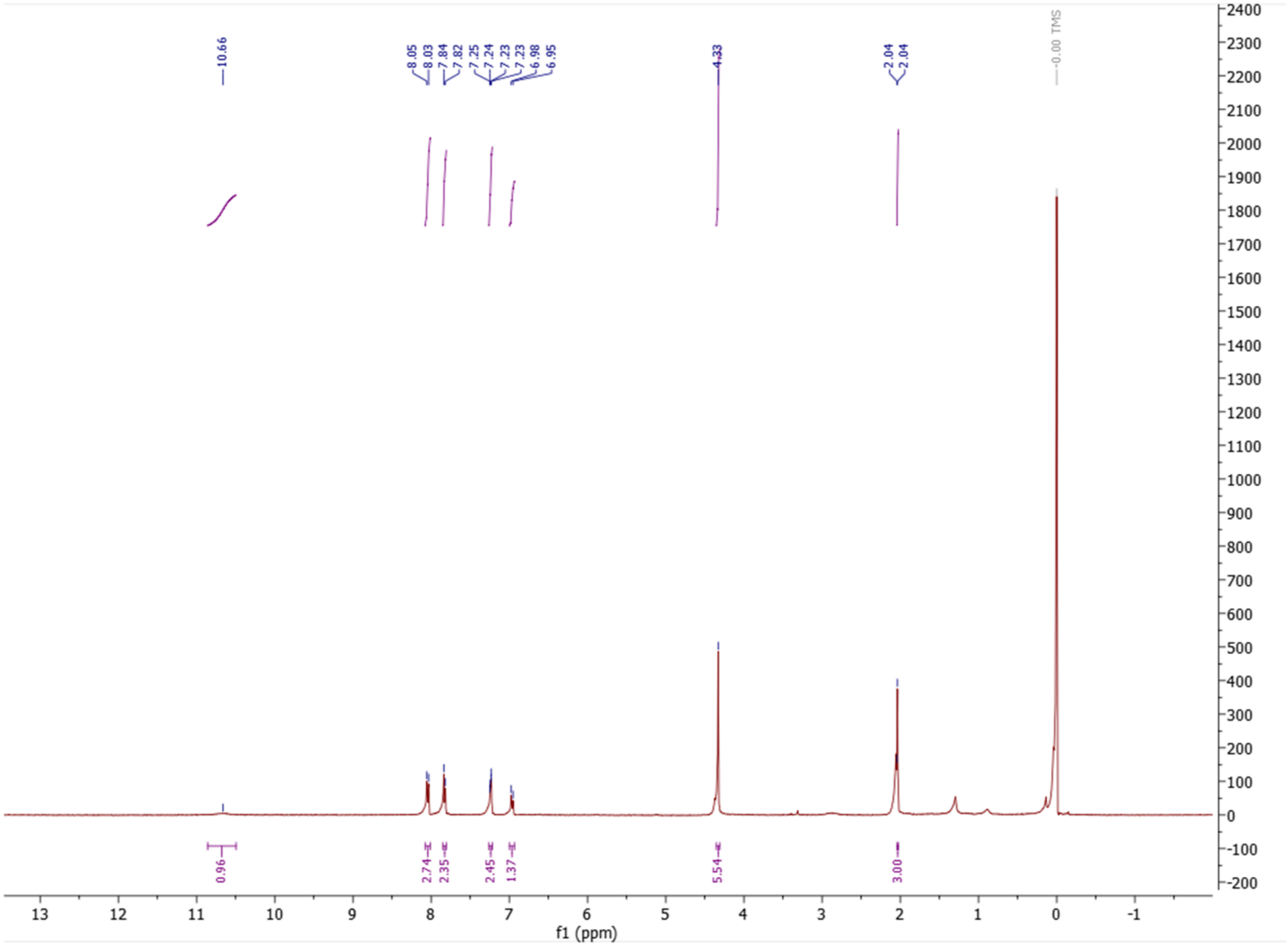

PAWS-A7, SZ35TA(Me)

^{1}H NMR (500 MHz) in $CDCl_3$

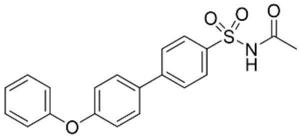

SZ35TA(Me)

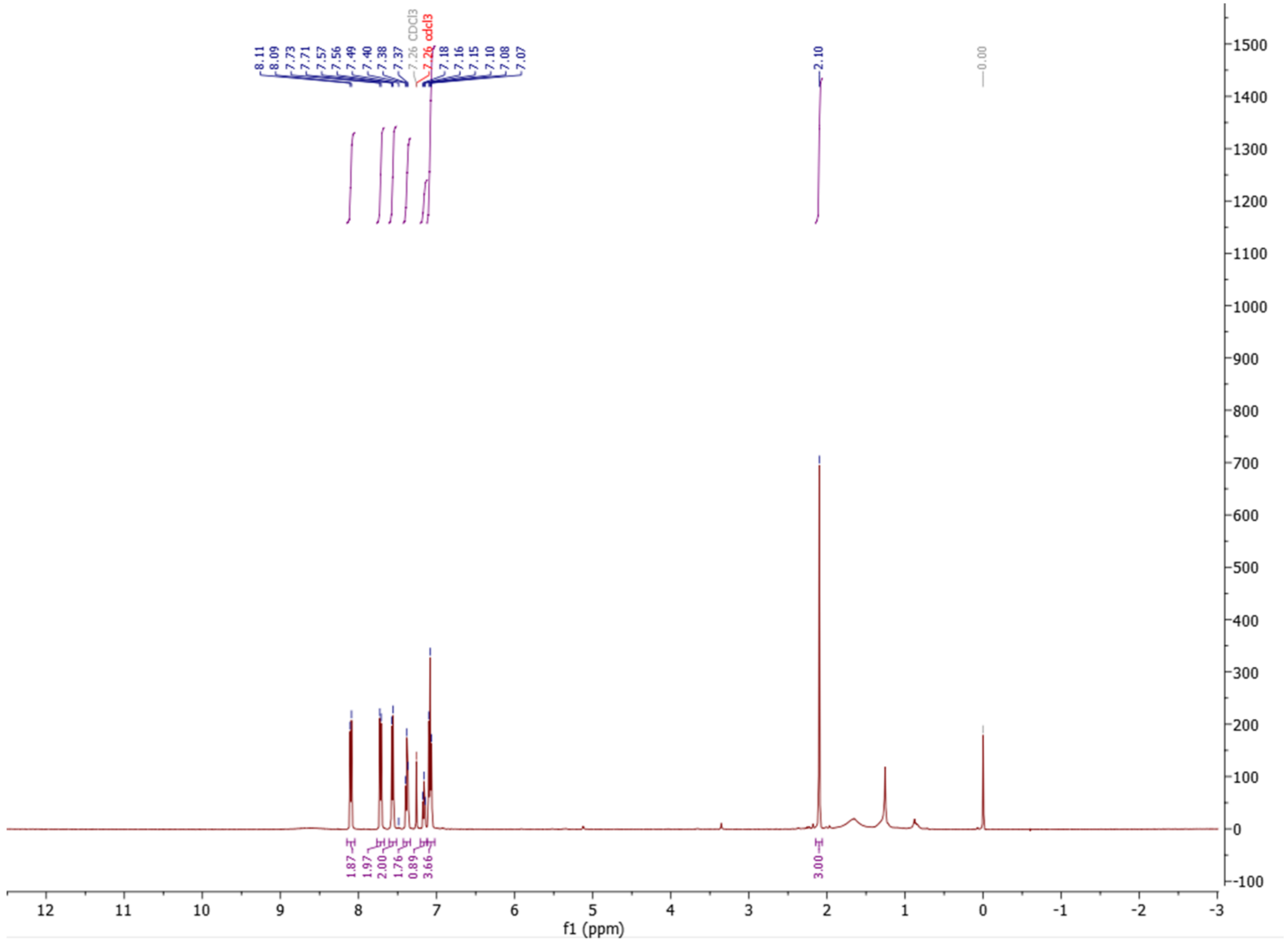

PAWS-A8, SZ37TA(Me)

^{1}H NMR (400 MHz) in $(CD_3)_2SO$

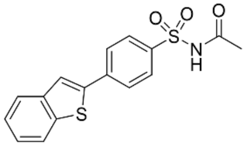

SZ37TA(Me)

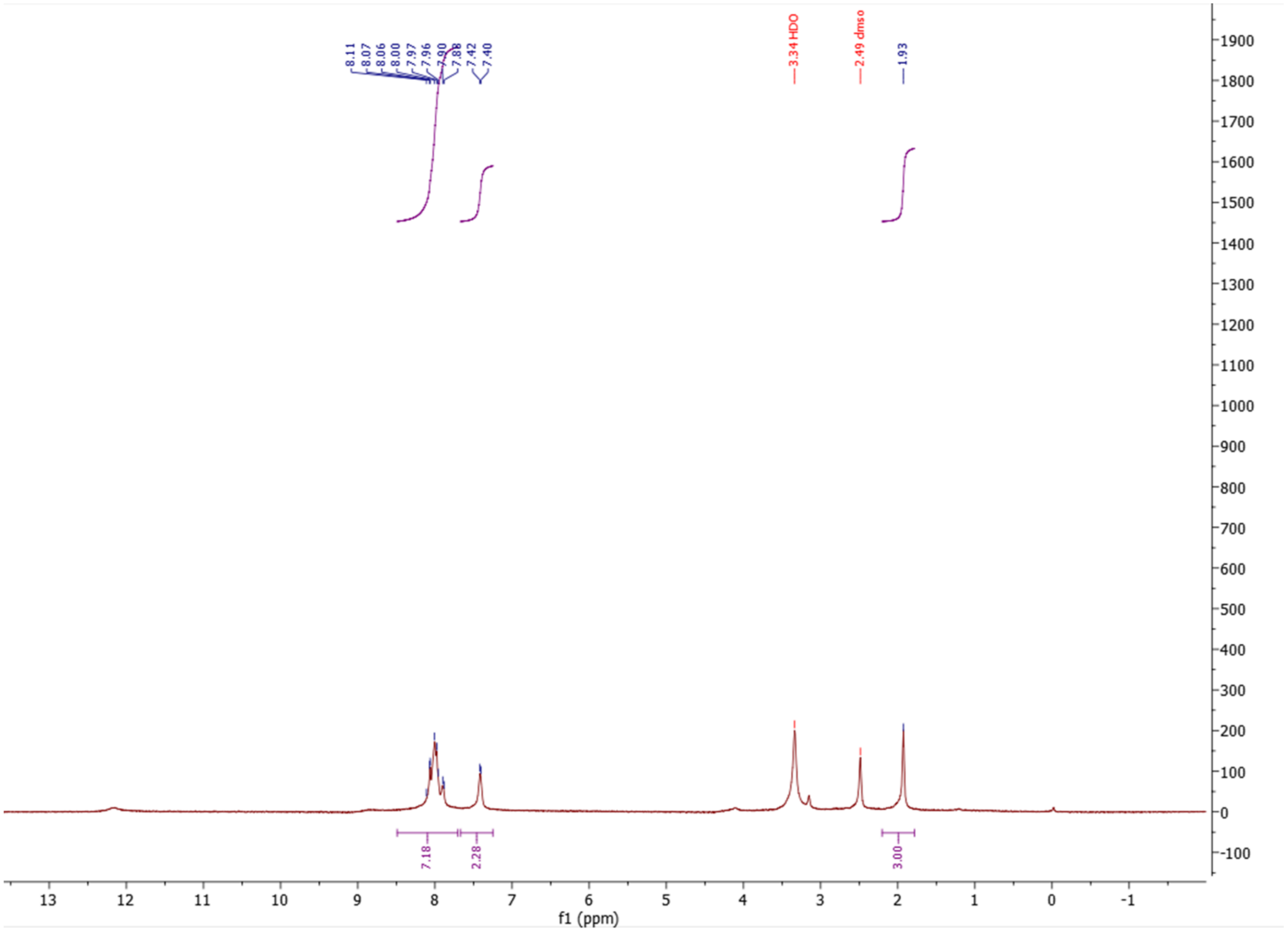

PAWS-A9, SZ16TA(Me)

^{1}H NMR (400 MHz) in $(CD_3)_2CO$

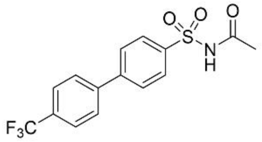

SZ16TA(Me)

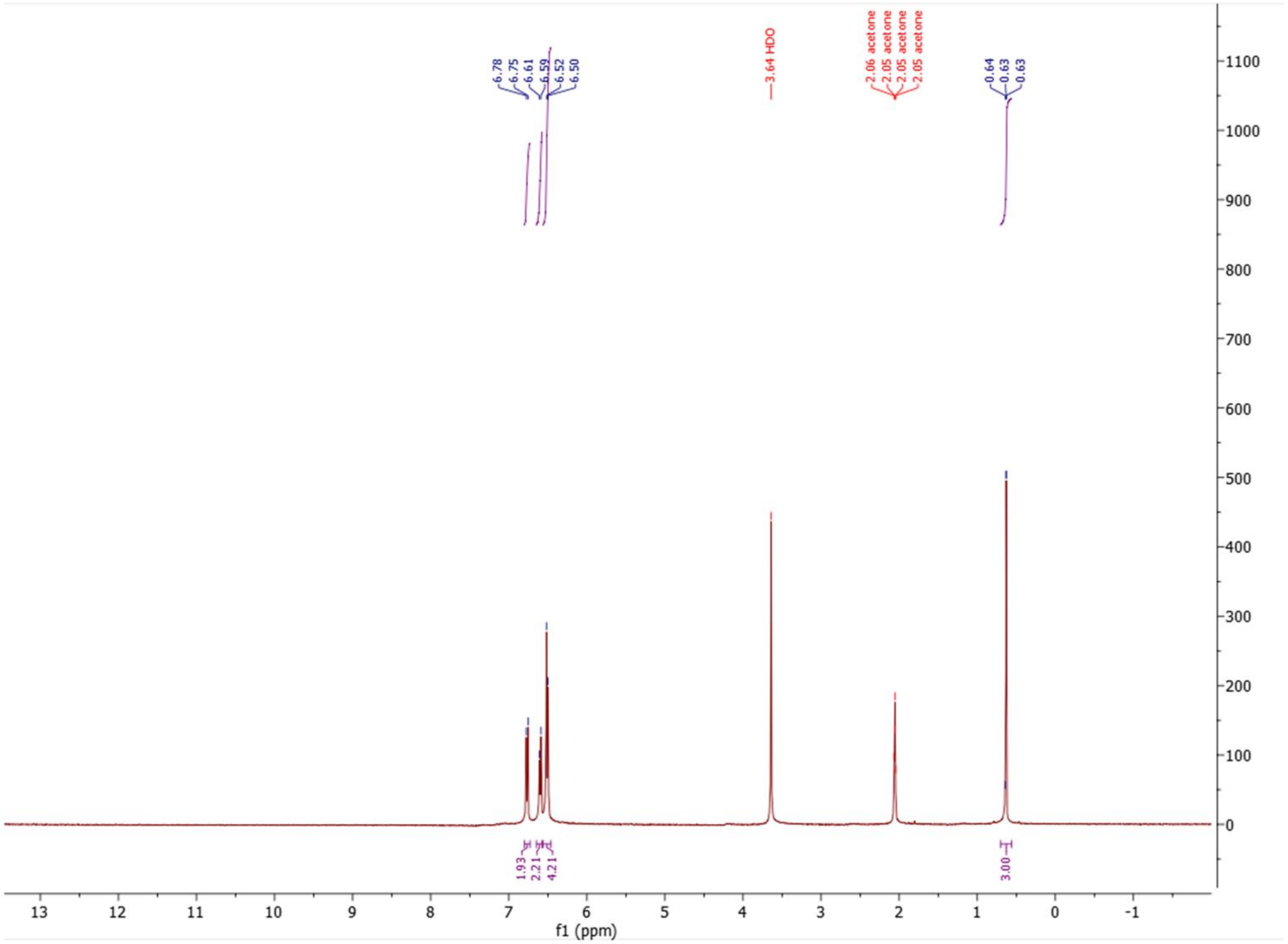

PAWS-A10, SZ13TA(Me)

^{1}H NMR (400 MHz) in $CDCl_3$

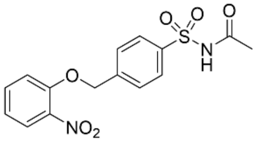

SZ13TA(Me)

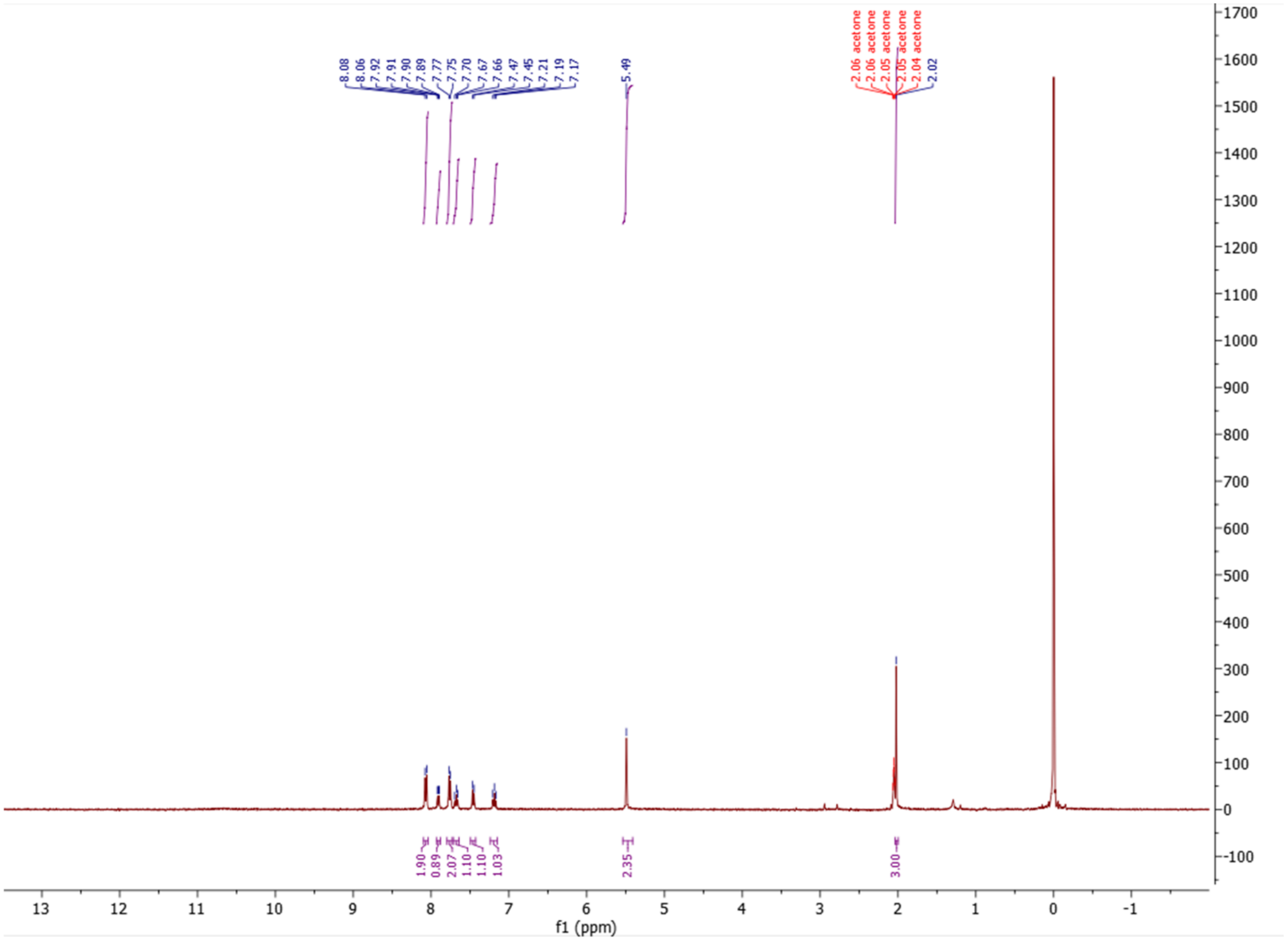

PAWS-A11, SZ1TA(Me)

^{1}H NMR (400 MHz) in $(CD_3)_2CO$

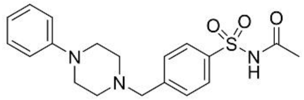

SZ1TA(Me)

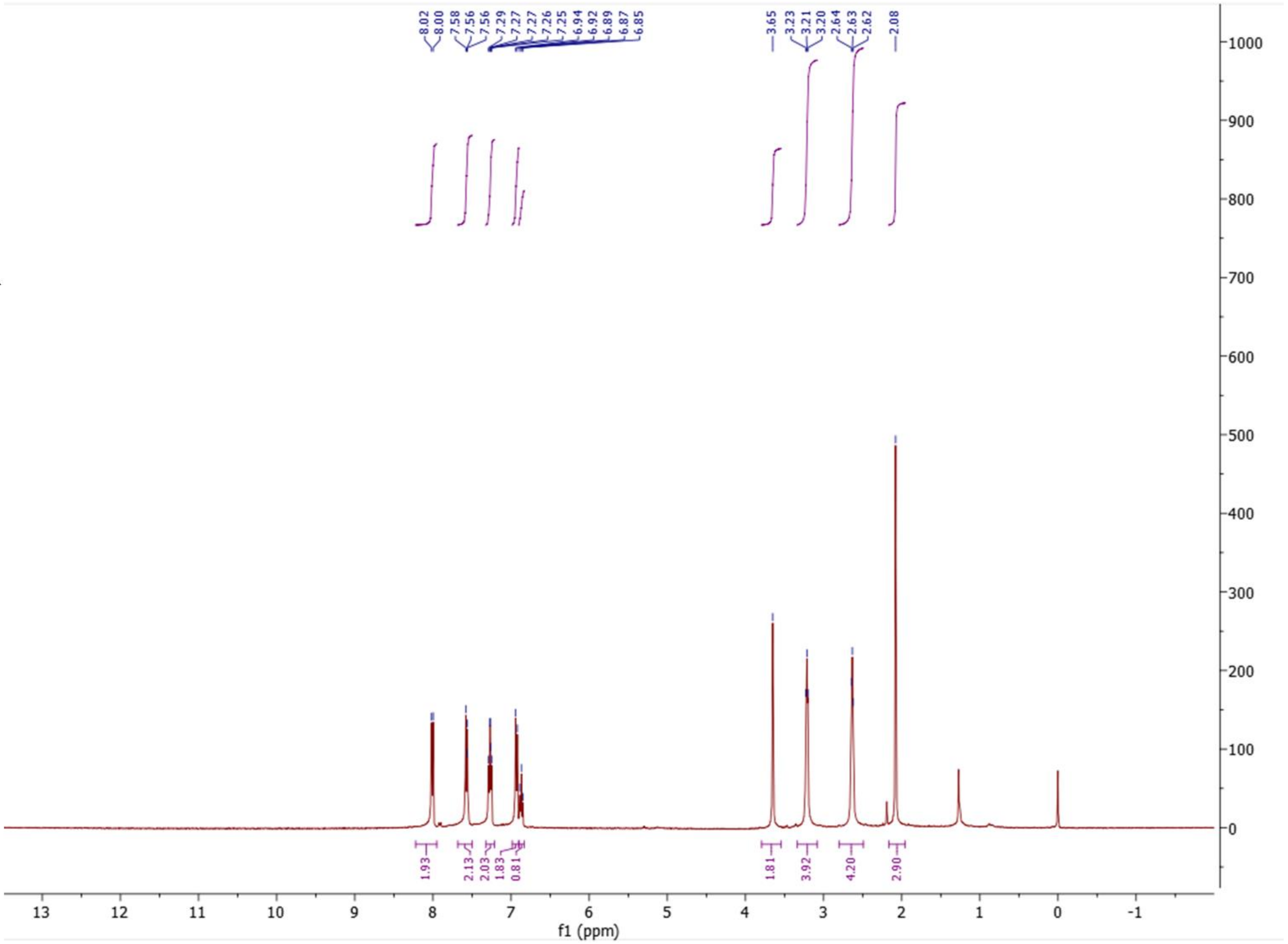

References

1. Baell, J. B.; Holloway, G. A., New Substructure Filters for Removal of Pan Assay Interference Compounds (PAINS) from Screening Libraries and for Their Exclusion in Bioassays. *J. Med. Chem.* **2010,** *53* (7), 2719-2740.
2. Wager, T. T.; Hou, X.; Verhoest, P. R.; Villalobos, A., Moving beyond rules: the development of a central nervous system multiparameter optimization (CNS MPO) approach to enable alignment of druglike properties. *ACS Chem. Neurosci.* **2010,** *1* (6), 435-49.
3. Oltersdorf, T.; Elmore, S. W.; Shoemaker, A. R.; Armstrong, R. C.; Augeri, D. J.; Belli, B. A.; Bruncko, M.; Deckwerth, T. L.; Dinges, J.; Hajduk, P. J.; Joseph, M. K.; Kitada, S.; Korsmeyer, S. J.; Kunzer, A. R.; Letai, A.; Li, C.; Mitten, M. J.; Nettesheim, D. G.; Ng, S.; Nimmer, P. M.; O'Connor, J. M.; Oleksijew, A.; Petros, A. M.; Reed, J. C.; Shen, W.; Tahir, S. K.; Thompson, C. B.; Tomaselli, K. J.; Wang, B.; Wendt, M. D.; Zhang, H.; Fesik, S. W.; Rosenberg, S. H., An inhibitor of Bcl-2 family proteins induces regression of solid tumours. *Nature.* **2005**, *435* (7042), 677-81.
4. Nacheva, K.; Kulkarni, S. S.; Kassu, M.; Flanigan, D.; Monastyrskyi, A.; Iyamu, I. D.; Doi, K.; Barber, M.; Namelikonda, N.; Tipton, J. D.; Parvatkar, P.; Wang, H. G.; Manetsch, R., Going beyond Binary: Rapid Identification of Protein-Protein Interaction Modulators Using a Multifragment Kinetic Target-Guided Synthesis Approach. *J. Med. Chem.* **2023**, *66* (4) 7, 5196 – 5207.
5. Wu, X.; Hu, L., Efficient amidation from carboxylic acids and azides via selenocarboxylates: application to the coupling of amino acids and peptides with azides. *J. Org. Chem.* **2007,** *72* (3), 765-74.
